# Comprehensive comparative metabolome study of a large collection of Corsican bryophytes

**DOI:** 10.3389/fpls.2024.1470307

**Published:** 2025-01-07

**Authors:** Anaïs Pannequin, Alain Muselli, Laurence Marcourt, Emerson Ferreira Queiroz, Luis-Manuel Quiros-Guerrero, Yoshinori Asakawa, Miwa Dounoue-Kubo, Jean-Luc Wolfender

**Affiliations:** ^1^ Université de Corse, Unité Mixte de Recherche du Centre national de la recherche scientifique (UMR CNRS) SPE 6134, Laboratoire Chimie des Produits Naturels, Corte, France; ^2^ Institute of Pharmaceutical Sciences of Western Switzerland, University of Geneva, Geneva, Switzerland; ^3^ School of Pharmaceutical Sciences, University of Geneva, Geneva, Switzerland; ^4^ Faculty of Pharmaceutical Sciences, Tokushima Bunri University, Tokushima, Japan

**Keywords:** bryophytes, liverworts, mosses, metabolomics, natural products, molecular networking, Corsican biodiversity

## Abstract

**Introduction:**

Bryophytes are non-vascular plants that appeared on Earth before vascular plants. More than 24,000 species are reported worldwide, and only a small proportion have been studied. However, part of their biosynthetic potential has been unveiled and more than 1,600 terpenoids have been detected and identified. The study of bryophytes faces challenges due to their small size, and sociology, making it difficult to collect large amounts of uncontaminated samples. Additionally, their chemical specificity and the scarcity of chemical data specific to this branch further complicate their study. Traditionally, research on bryophytes has focused only on specific species or classes of compounds.

**Methods:**

In contrast, our work proposes the first untargeted metabolite profiling investigation of a large collection of bryophytes (63 species) mainly issued from Corsican biodiversity. Metabolite profiling was performed by UHPLC-HRMS/MS and the data was extensively annotated using computational tools and molecular networking. This allowed us to describe in detail the chemical space covered by our collection and to establish comparisons between all the moss and liverwort species available. To validate some of the structural annotations, 3 liverworts (*Frullania tamarisci, Pellia epiphylla, Plagiochila porelloides*) and 2 mosses (*Antitrichia curtipendula* and *Dicranum scoparium*), available in larger quantities were fractionated using high-resolution semi-preparative HPLC, yielding 20 pure compounds. Five of them were newly discovered.

**Results and discussion:**

This study highlights the main compositional differences between mosses and liverworts at the chemical class level. By analyzing given molecular network clusters, specific biosynthetic features or compounds that are characteristic of certain species are highlighted and discussed in detail.

## 1 Introduction

Bryophytes are divided into three phyla: mosses (*Bryophyta*, 12,700 species), liverworts (*Marchantiophyta*, 9,000 species), and hornworts (*Anthocerotophyta*, 225 species). Taxonomically placed between green algae and vascular plants, bryophytes are the first land plants (Christenhusz and Byng, 2016). Despite having 24,000 species distributed worldwide, the number of studies on the chemistry of bryophytes is still limited. However, 2,200 compounds have been identified in such organisms, mainly volatile compounds from liverworts. A great proportion are new compounds, and many of them have proved to be biologically active (Asakawa et al., 2013a; Horn et al., 2021; Novaković et al., 2021).

Sampling represents one of the biggest challenges when working with bryophytes. They are small organisms that rarely grow in large quantities. They grow in complex communities. As a result, it is rare to find “clean” samples in the wild, i.e., free of other bryophytes, insects, and debris from vascular plants, etc. The quantity available per species is highly variable, and samples must be carefully cleaned (Asakawa and Ludwiczuk, 2013).

Among the bryophytes, the lipophilic terpenoids of liverworts have been the most extensively studied due to the existence of intracellular structures containing oil droplets, known as *oil bodies*, which are characteristic of the phylum. These specialized organelles contain a range of lipophilic molecules, mostly mono- and sesquiterpenoids. Liverworts have therefore been the subject of a great deal of research into their volatile terpenes in essential oils and lipophilic extracts. Liverworts are described as an abundant source of new natural sesquiterpenoids with a wide variety of carbon skeletons (Asakawa, 2012; Asakawa et al., 2013b).

Mosses, in contrast, have not been broadly investigated, despite being an abundant class of bryophytes. They are known to contain high levels of fatty acids, making the detection/isolation of secondary metabolites challenging. Flavonoids and some di- and triterpenoids are the most common other components detected in mosses (Asakawa et al., 2013b; Lu et al., 2019).

Bis-bibenzyls and bibenzyls are listed as remarkable and specific constituents of bryophytes because of their important therapeutic role. They are frequently found in dimeric or polymeric forms in polar solvent extracts of liverworts, such as marchantin and riccardin (Asakawa et al., 2021). Finally, nitrogenated compounds are very rare in bryophytes. Only nine compounds have been described in liverworts and none in mosses: skatole, two prenyl indole derivatives, isotachin A and B, two coriandrins, and two methyl tridentatol (von Reuß and König, 2005). Noteworthy, such data were summarized in three extensive reviews by Asakawa et al (Asakawa, 1982, 1995; Asakawa et al., 2013b).

Modern analytical approaches enable the high-throughput analysis of a large number of plant extracts from small quantities (Wolfender et al., 2019). These approaches mainly rely on the metabolite profiling of a crude extract with ultra-high-performance liquid chromatography coupled with high-resolution mass spectrometry (UHPLC–HRMS). On such a platform, data-dependent analysis enables high-resolution tandem mass spectrometry (HRMS/MS) fragmentation spectra to be recorded on most detected compounds with unprecedented sensitivity. Annotation is then carried out by spectral comparison with public libraries [Global Natural Products Social Molecular Networking (GNPS)] (Nothias et al., 2020) and/or by computational tools [SIRIUS, Taxonomically Informed Metabolite Annotation (TIMA), etc.] (Dührkop et al., 2019; Rutz et al., 2019), generating precise information on the chemical composition of the extracts. This makes it possible to study a large number of species simultaneously to define compositional traits and prioritize extracts for targeted isolation and full characterization of selected metabolites (Allard et al., 2023). Such metabolomic approaches are being used more often to study the metabolome of vascular plants (Shen et al., 2023). To date, only a few studies have been published using this kind of approach on a small collection to establish chemotaxonomic correlations (Peters et al., 2019, 2021).

The present study focuses mainly on the UHPLC–HRMS/MS metabolite profiling of a representative set of bryophytes from the Corsican biodiversity. In Corsica, over 574 species have been reported (Sotiaux et al., 2007, 2008). Only four species were chemically investigated, and this was limited to the profiling of the essential oils (Pannequin et al., 2017, 2020, 2023; Pannequin, 2019). For this, 60 species including liverworts and mosses were extracted with solvents of increasing polarity. All extracts were systemically profiled by UHPLC–HRMS/MS under the same conditions, and all the data were gathered in a massive molecular network. As only a few reference MS/MS spectra were available, targeted isolation was carried out on abundant species for the unambiguous identification of numerous metabolites, which were used to increase confidence in the annotation.

## 2 Results

### 2.1 Design and validation of molecular network

#### 2.1.1 Generation of an extract collection of bryophytes

A total of 60 Corsican species, including 14 liverworts and 46 mosses, were collected. Additionally, three Japanese liverworts, as part of a collaboration with the Tokushima Bunri University (Japan), were added to the collection. **Supplementary Table S1** summarizes the data related to the extract collection, including taxonomical information and yield (**Supplementary Figure S1**; **Supplementary Table S1**).

Bryophytes, in particular mosses, are known to produce many fatty acids. These primary metabolites are common to many vascular plants, and in this study, they were not profiled (Lu et al., 2019). They were depleted to obtain an enrichment of secondary metabolites, which are known to be more species-specific. To reduce their amounts and improve the detection of metabolites of medium polarity, the dry sample material was extracted successively with hexane (HEX), methylene chloride (DCM), and methanol (MeOH). Only the DCM and MeOH extracts were further analyzed. The means of extraction yields of liverworts and mosses are similar, respectively: 0.21% and 0.25% for HEX extracts, 0.58% and 0.53% for DCM extracts, and 1.93% and 2.01% for MeOH extracts. However, boxplots of extraction yields (**Supplementary Figure S2**) show high variability between species independently of the phylum. Several species appear as outliers: three liverworts (*Frullania tamarisci*, *Porella arboris-vitae*, and *Porella obtusata*) with extraction yields of 1.7%, 1.5%, and 3.4%, respectively, to DCM extracts and 2.9%, 4.7%, and 4.4%, respectively, to MeOH extracts and two mosses (*Bartramia pomiformis* and *Orthotrichum rupestre*) with 6.3% and 6.0%, respectively, to MeOH extract yields, three times more than the average.

The composition of the HEX extracts was evaluated by ^1^H NMR profiling. This revealed that they contained mainly fatty acids. The DCM and MeOH extracts were submitted to Solid Phase extraction (SPE) to reduce the amount of residual fatty acids and highly polar metabolites (this process is referred to as “sample clean-up” and continued in **Supplementary Figure S3**). After sample clean-up, on average, DCM moss extracts lost 83% of their mass, while liverwort extracts lost 60%, which highlights the presence of lipids even after hexane defatting of the dried plant material. MeOH moss extracts lost 61% of their mass after cleaning, while liverwort extracts lost only 45%. These last losses may be attributed to the presence of polar residues (mainly sugars based on ^1^H NMR profiling).

The comprehensive approach to secondary metabolite enrichment indicates that natural products of medium polarity (DCM + MeOH) comprise 12.9 mg per gram of dry liverworts and 7.8 mg per gram of dry mosses. This shows that mosses produce about half the amount of secondary metabolites compared with liverworts. In addition to the ends in the mean values, extraction yields were found to be very species-specific; for example, *P. arboris-vitae* yielded 35.9 mg per g of the dry plant (MeOH extract). For most details, refer to the boxplot figure in **Supplementary Figure S2**.

#### 2.1.2 Comprehensive UHPLC–HRMS/MS metabolite profiling of bryophyte extracts

All enriched DCM and MeOH extracts were systematically profiled by reversed-phase UHPLC–HRMS/MS in positive (PI) and negative ionization (NI) modes using a generic 9-min linear gradient. This yielded 252 chromatographic profiles: 126 in PI and 126 in NI.

Despite the sample clean-up procedure, evaluation of the metabolite profile of an initial set of species still revealed residual features associated with fatty acids after 6 min of elution and sugars between 0 and 1 min of elution. To focus on secondary metabolites, only features detected within a chromatographic window of 1 to 6 min were retained for further data exploration.

**Figure 1:**
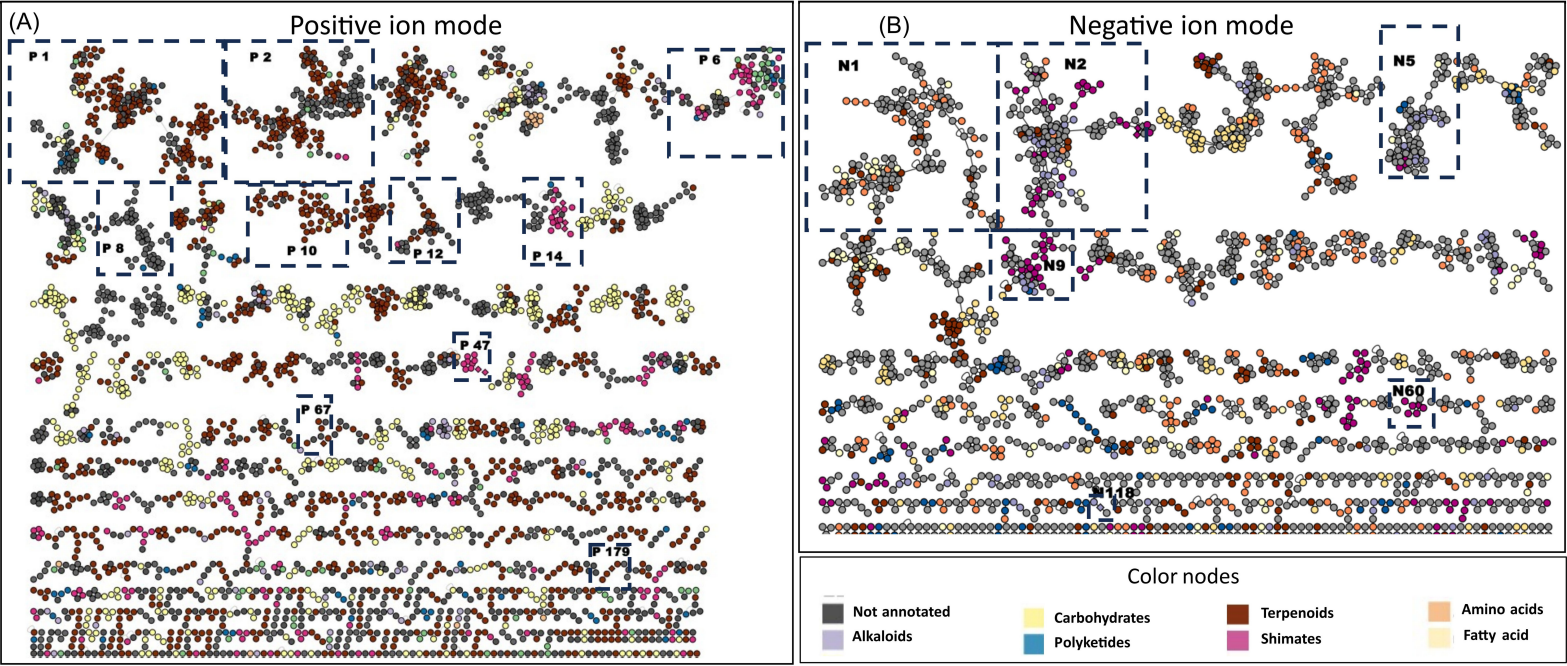
Ion identity molecular network of the DCM and MeOH extracts of bryophyte species **(A)** in PI and **(B)** in NI including clusters larger than two nodes. Nodes are colored based on the confident NPClassifier chemical pathway. Validated clusters are numbered and framed. DCM, methylene chloride; MeOH, methanol; PI, positive ionization; NI, negative ionization.

Both PI and NI data were gathered in two separate feature-based molecular networks (MNs): PI, 8,843 nodes including 5,252 grouped in 370 clusters; and NI, 4,572 nodes including 2,660 nodes grouped in 190 clusters (**Figure 1**). For ease of discussion, clusters have been numbered from largest to smallest (PI clusters are preceded by P and NI by N).

#### 2.1.3 Annotation of detected metabolite

All HRMS/MS spectra of the detected features were annotated using the SIRIUS software (Dührkop et al., 2015, 2021; Ludwig et al., 2020; Hoffmann et al., 2022) and, in particular, the CANOPUS module, which provides confident chemical class information using the deep neural network-based structural classification tool NPClassifier that provides pathways, superclasses, and class information (Dührkop et al., 2021).

To establish a list of individual candidate structures for all features detected with an MS^2^ spectrum, a workflow that initially performed a spectral matching against an *in-silico* MS/MS database of NP spectra was used. For this, a modified cosine score was calculated between experimental and simulated spectra (Allard et al., 2016). Candidate structures were then reweighted based on the taxonomy of the source organism and in the light of previously reported occurrences gathered by the LOTUS initiative (Rutz et al., 2022). This automated workflow is known as TIMA (Rutz et al., 2019). All these annotations are available in the MASSIVE repository of the data (see *Materials and methods*).

For all features of interest displayed in the figures, only the best structural candidates proposed by TIMA matching the NPClassifier superclass from SIRIUS were considered valid. In cases where TIMA was not giving coherent annotation proposals, the GNPS annotations automatically obtained during the molecular network construction were considered.

This information was mapped on the MN using color-coded nodes according to the SIRIUS pathway annotation, giving a quick overview of the chemical diversity of the extracts (**Figure 1**).

It is generally accepted that the annotations of pathways and superclass via SIRIUS have a high level of confidence (expressed as probability hereafter) (Dührkop et al., 2021). In this study, only pathway and superclass annotations with a probability greater than 0.8 in PI and 0.7 in NI were exploited. Using this protocol, 52% of all detected PI features could be attributed to a given NP pathway with good confidence (4,467 features in the dataset). Among them, 51% were annotated with structures derived from TIMA.

Over the whole dataset, significantly more PI than NI features were detected. While the PI mode provided detection of most compound types, for the fatty acids, shikimate, and phenylpropanoids (flavonoids, stilbenoids, etc.), better ionization and detection were obtained in NI. Thus, because of this complementary, both modes were kept in the data meaning to obtain a comprehensive overview of the metabolite repartition.

To summarize most of the annotation results obtained and to facilitate comparison across all samples, the number of features annotated in each superclass was gathered by species.

**Figure 2:**
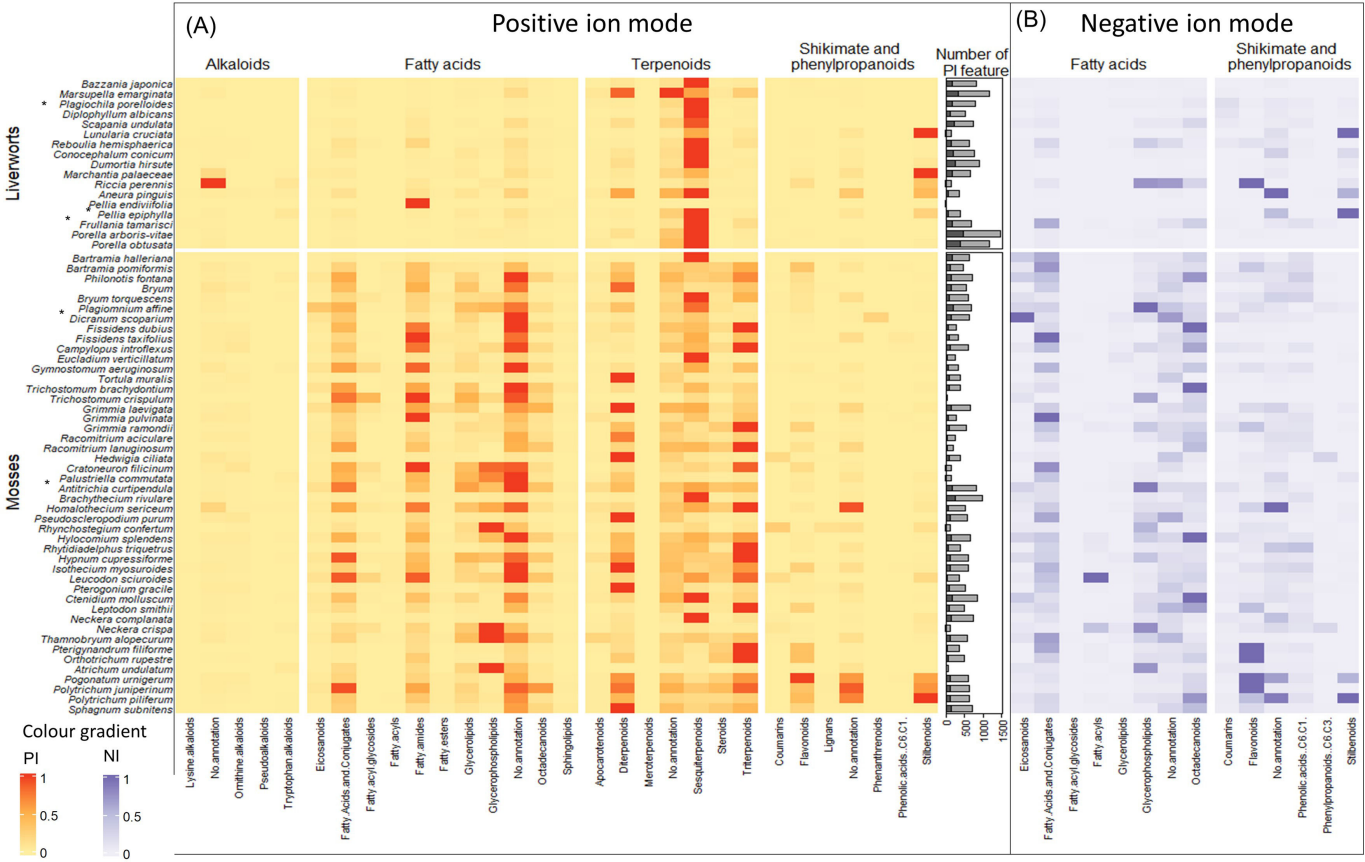
Heatmap illustrating the richness of chemical entities in the compound superclasses of bryophyte species in **(A)** PI and **(B)** NI. The color gradient corresponds to the proportion of annotated chemical superclasses in each species. Only superclasses that are the sum of the areas greater than 5E5 are represented. **(A)** The heatmap shows the richness of compound superclasses in PI. Only annotations with cosine score greater than 0.8 are shown. Compound classes with the highest abundance in the species are shown in red, and those with the lowest abundance are shown in yellow. The bar plot shows the number of features divided into annotated features in light gray and unannotated features in dark gray. **(B)** The heatmap shows the abundance of fatty acids, and shikimates and phenylpropanoids in the bryophyte species in NI. Only annotations with probability greater than 0.7 are shown. Dark blue represents the classes of compounds that are most abundant in the species and light blue those that are least abundant. PI, positive ionization; NI, negative ionization.

The 3,120 PI and 1,049 NI features were associated with 28 and 14 superclasses, respectively (all the 14 NI superclasses were also annotated in PI). For each sample, the feature intensities of each superclass were summed, assessing the occurrence of the superclass in each organism. To obtain a clearer understanding of the results, the proportion of chemical superclass annotations per species was displayed as a heatmap in the PI mode that was supplemented specifically for fatty acids, shikimates, and phenylpropanoids in the NI mode. For ease of reading, only superclasses whose sum area is greater than 5E5 were kept in the heatmap (**Figure 2**). In addition, a bar plot representing the number of annotated *vs.* unannotated features per species is displayed, indicating the richness of metabolites in each species and their annotation ratio.

The interpretation of these heatmap results and comparison across mosses and liverworts together with a detailed discussion of specific clusters of the network are provided in section 2.2.

#### 2.1.4 Isolation of metabolites used to validate annotations

Most of the results obtained are based on spectral comparisons using computational approaches. Before entering a more detailed discussion of the MN and the specific annotation of metabolites, targeted isolation of compounds was performed to obtain a maximum number of standards for unambiguous identification of features in the molecular networking (nodes).

The nodes corresponding to isolated standards were used as anchor points to check the consistency of annotations within MN clusters and for annotation propagation purposes (Nothias et al., 2020).

To this end, five species of interest were selected based on their MN, heatmap, and the availability of plant material, and therefore extract, available in the collection. Specifically, two liverworts, *F. tamarisci* and *Plagiochila porelloides*, known to produce terpenoids such as eudesmanolides (Pannequin et al., 2017) and plagiochilines, respectively (Pannequin et al., 2023), which are strongly represented in clusters P1 and P2 (**Figures 1**, **2**), were chosen for the targeted isolation of the sesquiterpenoids. *Pellia epiphylla* is another liverwort producing specific stilbenoids and was selected for the isolation of perrottetin-type bis-bibenzyls (Cullmann et al., 1997).

**Figure 3:**
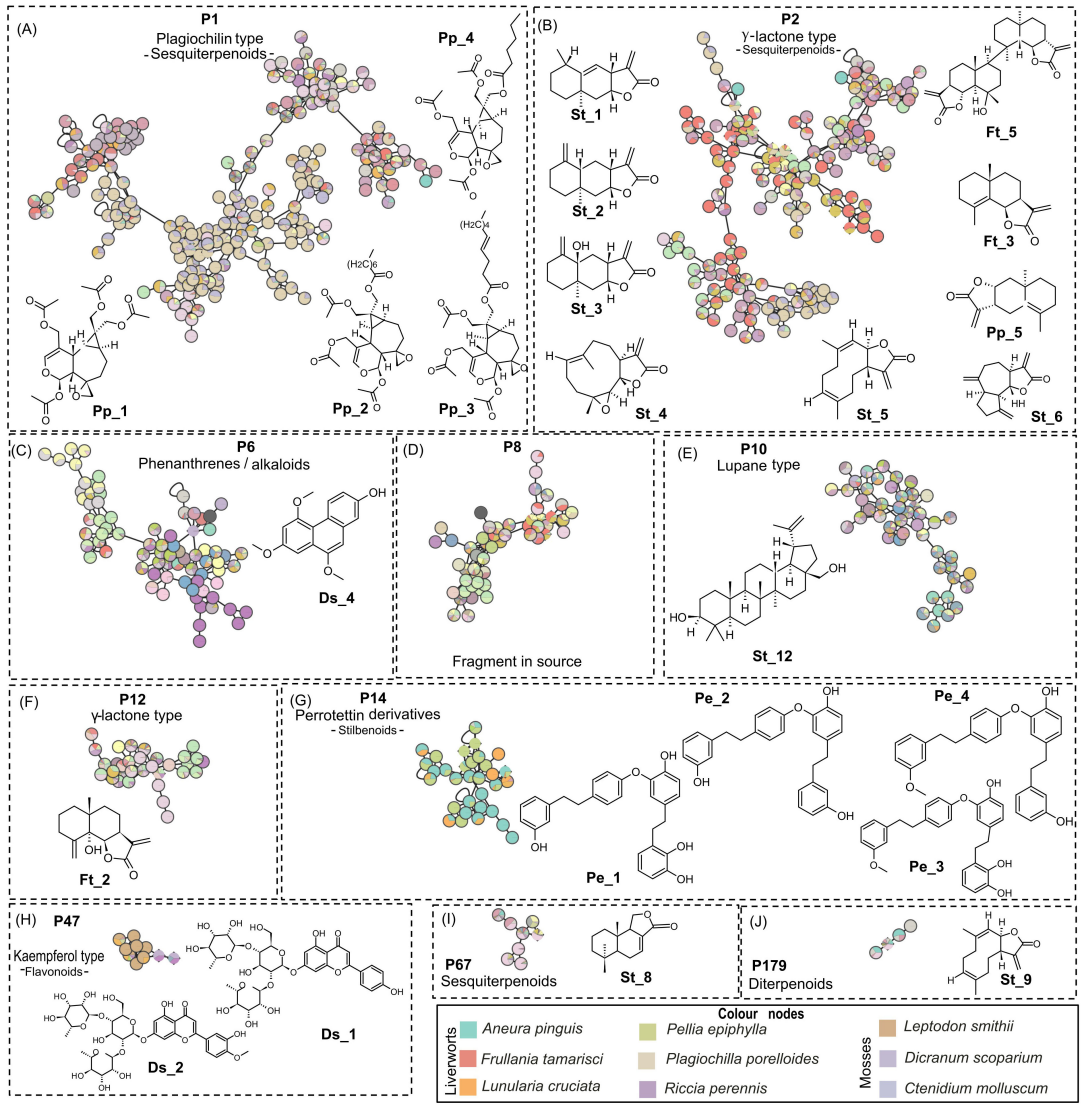
Selected PI clusters from **Figure 1**, validated by standard or isolated compounds: **(A)** P1, **(B)** P2, **(C)** P6, **(D)** P8, **(E)** P10, **(F)** P12, **(G)** P14, **(H)** P47, **(I)** P67, and **(J)** P179. The pie charts within node indicate the distribution of MS intensities of features across the species. Nodes with single color are characteristic of metabolites found only in a specific species. Nodes with multiple colors denote shared metabolites. Clusters shared by multiple species highlight either specific shared features and/or shared chemical classes. Species-specific clusters correspond to one color node. Color node codes are available in the **Supplementary Material**. PI, positive ionization.

Finally, as mosses have been little studied in the literature, two mosses, *Antitrichia curtipendula* and *Dicranum scoparium*, were selected to complete the selection. The metabolite profiling results indicated that they produce flavonoids and phenanthrenes.

To isolate the main constituents of these five bryophyte species, a generic isolation approach based on a one-step fractionation of the extracts using high-resolution semi-prep HR-HPLC was developed.

Before separation and to maximize separation efficiency, the extracts of the five selected species were subjected to a protocol allowing significant secondary metabolite enrichment. This “sample enrichment” protocol combined a two-step liquid–liquid extraction followed by SPE enrichment of the medium polarity partition (see **Supplementary Figure S3**). This protocol enables sugars and fatty acids to be removed efficiently on a gram scale from the crude extract.

For example, the “sample enrichment” protocol applied to 1 g of crude MeOH extract of *D. scoparium* (moss) yielded 40 mg of enriched extract (i.e., a loss of 96% of the initial mass) (**Supplementary Figure S3**). The enriched secondary metabolites represented less than 0.4 mg per g of dry plant material (0.04%) (for all extracts, see **Supplementary Table S1**).

The chromatographic conditions were first optimized on a reversed-phase C18 column on an HPLC–photodiode array (PDA)–evaporative light scattering detector (ELSD) instrument at the analytical scale. This optimized gradient was geometrically transferred to the semi-preparative HPLC scale (Guillarme et al., 2008). The enriched extracts were injected using a dry loading method to maintain high chromatographic resolution (Queiroz et al., 2019).

For ease of understanding, the code of each molecule consists of the acronym of the origins (Ac, *A. curtipendula*; Ds, *D. scoparium*; Ft, *F. tamarisci*; Pe, *P. epiphylla*; Pp, *P. porelloides*; and St, standard origin) followed by a number.

**Figure 4:**
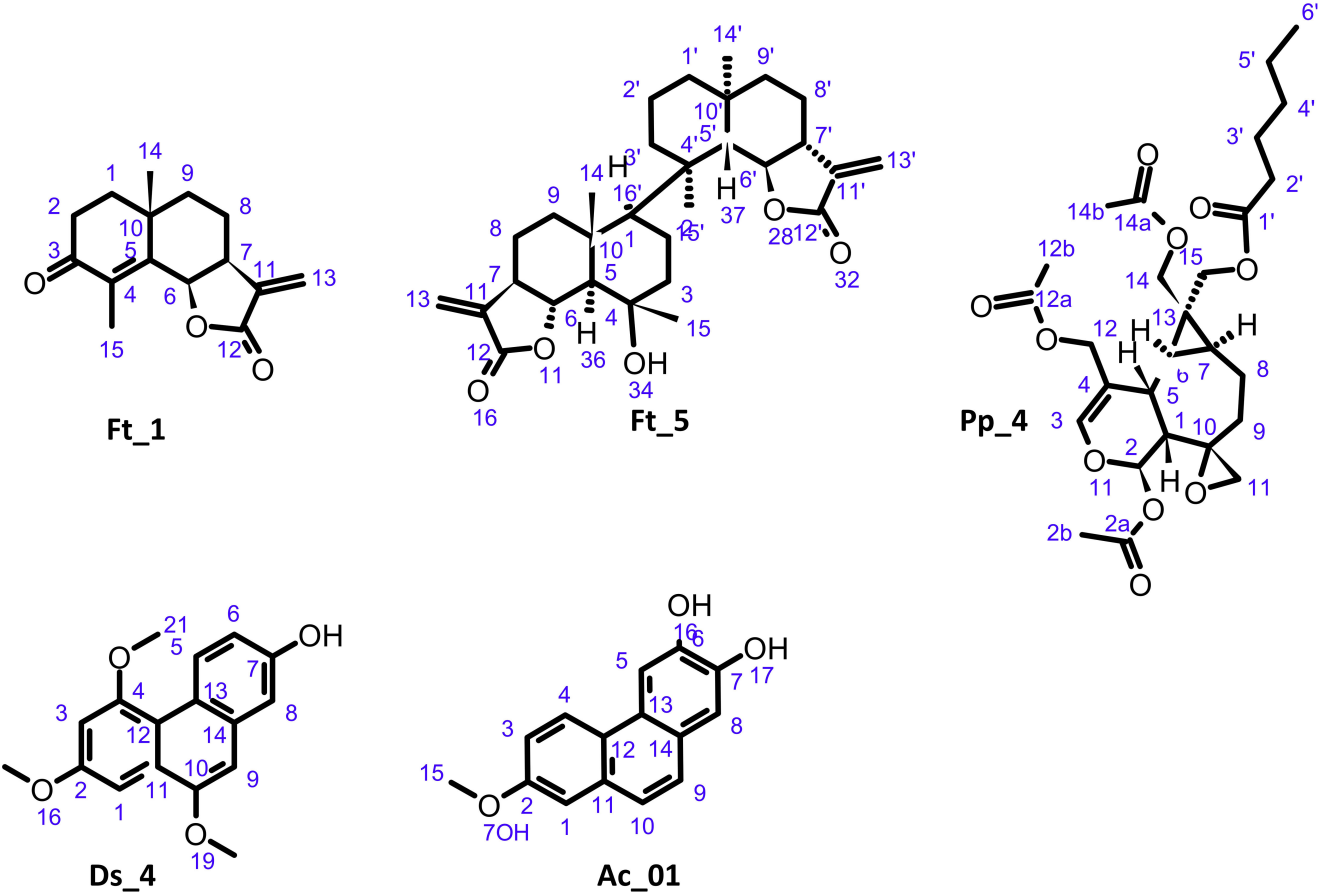
Structure of undescribed compounds: oxo-frullanolide Ft_1, tamariscolide Ft_5, plagiochiline *R*-15-yl hexanoate Pp_4, 7-hydroxy-2,4,10-trimethoxyphenanthrene Ds_4, and 6,7-dihydroxy-2-methoxyphenanthrene Ac_01.

In the case of *D. scoparium*, four compounds were isolated and fully characterized by HRMS, NMR, and UV. Among them, three flavanones previously reported in *D. scoparium* were identified: apigenin 7-*O*-[2,4-di-*O*-(α-l-rhamnopyranosyl)]-β-d-glucopyranoside (Becker et al., 1986), Ds_1; 7-[(*O*-6-Deoxy-α-l-mannopyranosyl-(1→2)-*O*-[6-deoxy-α-l-mannopyranosyl-(1→4)]-β-d-glucopyranosyl)oxy]-5-hydroxy-2-(3-hydroxy-4-methoxyphenyl)-4*H*-1-benzopyran-4-one (Osterdahl, 1978), Ds_2; and kaempferol-3-β-d-(6-*O*-*trans*-*p*-coumaroyl)glucopyranoside (Tsukamoto et al., 2004), Ds_3. A new phenanthrene was also isolated (Ds_4), and the ^1^H NMR spectrum showed the presence of three aromatic groups. A 1,3,4-trisubstituted benzene was identified from the aromatic protons at δ_H_ 6.93 (1H, dd, *J* = 9.2, 2.7 Hz, H-6), 7.12 (1H, d, *J* = 2.7 Hz, H-8), and 9.17 (1H, d, *J* = 9.2 Hz, H-5). A tetrasubstituted one was characterized by the two *meta*-coupled protons at δ_H_ 6.87 (1H, d, *J* = 2.6 Hz, H-3) and 7.29 (1H, d, *J* = 2.6 Hz, H-1). A singlet at δ_H_ 7.06 (1H, s, H-9) belongs to the third cycle. The Rotating-frame Overhauser Effect (ROE) correlations from the methoxy group at δ_H_ 3.89 to H-1 and H-3 positioned it in C-2; the one at δ_H_ 4.04 was placed in C-4 thanks to its correlation to H-3, while the third one at δ_H_ 4.02 was in C-10 due to its correlation with H-1 and H-9. The ROE spectroscopy (ROESY) from the hydroxyl at δ_H_ 9.54 to H-6 and H-8 located it at C-7. The heteronuclear multiple-bond correlations (HMBC) confirmed the identification of Ds_4 as 7-hydroxy-2,4,10-trimethoxyphenanthrene. The structure of Ds_4 with carbon numbering is shown in **Figure 4**, which illustrates all new structures.

The same method was applied to the other species. From *A. curtipendula*, the procedure enabled the isolation and identification of a previously undescribed phenanthrene. The ^1^H NMR spectrum showed the presence of a 1,3,4-trisubstituted benzene characterized by the three aromatic protons at δ_H_ 7.20 (1H, dd, *J* = 9.0, 2.8 Hz, H-3), 7.33 (1H, d, *J* = 2.8 Hz, H-1), and 8.33 (1H, d, *J* = 9.0 Hz, H-4); two *ortho*-coupled protons (*J* = 8.8 Hz) at δ_H_ 7.49 and 7.59, which belong to the second ring; and two singlets at δ_H_ 7.18 and 7.89 positioned therefore in *para* form the third ring. The HMBCs from the two singlets, H-5 and H-8, to the carbons at δ_C_ 145.9 (C-7) and 147.0 (C-6) indicated the presence of hydroxy groups in these positions. The correlations from H-4 and the methoxy group at δ_H_ 3.88 to the carbon at δ_C_ 156.9 (C-2) placed the methoxyl in C-2. The ROESY correlations from H-1 to H-10, H-9 to H-8, and H-5 to H4 and from the methoxyl to H-1 and H-3 confirmed that Ac_1 was a 6,7-dihydroxy-2-methoxyphenanthrene.

Six compounds have been isolated from the liverwort *F. tamarisci*, five of which correspond to eudesmanolide sesquiterpenes and one to the known triterpene ursolic acid Ft_6. Three eudesmanolide derivatives were known in the *Frullania* genus: oxy-frullanolide Ft_2 was previously isolated in *Frullania dilatata* (Asakawa et al., 1981), and frullanolide Ft_3 and γ-cyclocostunolide Ft_4 were commonly reported as *F. tamarisci* components (Pannequin et al., 2017).

A new *cis*-fused isomer of oxo-cyclocostunolide Ft_1 was characterized. The *cis* configuration of the lactone was in agreement with the ^3^ *J*_H6-H7_ coupling constant of 6 Hz, whereas it was 10 Hz in the *trans*-fused lactone-like oxo-cyclocostunolide (Nadgouda et al., 1978). The ^13^C chemical shift values of CH-6 and CH-7 were also a good indicative of the *cis* or *trans* configuration: they were reported in *trans*-eudesmanolides like arbusculin A (Fan et al., 2016) at δ_C_ 82.2 and 51.4, respectively, and in *cis* series like frullanolide (Ft_3) at δ_C_ 76.1 and 41.4, respectively. In Ft_1, the CH-6 and CH-7 carbons were observed at δ_C_ 75.6 and 40.6, respectively, and confirmed the identification as oxo-frullanolide.

Interestingly, a new eudesmanolide dimer Ft_5 with close similarities to muscicolide A previously described in *Frullania musciola* (Kraut et al., 1994) was also identified. In HRMS, the spectrum of Ft_5 did not show [M+H]^+^, but the ions at *m/z* 465.3061 and *m/z* 500.3391 corresponded to [M–H_2_O+H]^+^ and [M+NH_4_]^+^, respectively. An ion at *m/z* 233.1542 appears in the HRMS spectrum and MS/MS spectra. This can be corresponded to monomeric fragments of Ft_5. Due to the high overlap of NMR signals of each monomer, the relative configuration of Ft_5 was difficult to assign. However, the NOE correlations from H-6 at δ_H_ 4.05 to H_3_-14 at δ_H_ 1.09 and H_3_-15 at δ_H_ 1.30 indicated the axial position of these protons and that they were on the same side. For the same reasons that explained those previously for oxo-frullanolide Ft_1, the ^13^C chemical shift values of CH-6 and CH-7 (and CH-6′ and CH-7′) at δ_C_ 82.2 and 49.6 (and 82.2 and 51.4), respectively, indicated that H-6 and H-7 (and H-6′ and H-7′) were in a *trans* configuration. The ^13^C NMR chemical shift values of CH-5 and CH_3_-14 were also a good indicative of their relative configuration since they were observed in muscicolide A at δ_C_ 55.2 and 19.9 for CH-5 and CH_3_-14, respectively (H-5 and H_3_-14 being *trans*), and 49.3 and 21.2 for CH-5′ and CH_3_-14′, respectively (H-5′ and H_3_-14′ being *trans*), and in muscicolide B at δ_C_ 55.4 and 18.3 for CH-5 and CH_3_-14, respectively (H-5 and H_3_-14 being *trans*), and 45.5 and 32.0 for CH-5′ and CH_3_-14′, respectively (H-5′ and H_3_-14′ being *cis*) (Kraut et al., 1994). In Ft_5, the CH-5 and CH_3_-14 (CH-5′ and CH_3_-14′) were observed at δ_C_ 59.8 and 19.2 (53.4 and 21.6), respectively, indicating a *trans* configuration of H-5 and H_3_-14 as well as H-5′ and H_3_-14′. The ^13^C chemical shift values of CH_3_-15 (δ_C_ 24.7) and CH_3_-15′ (δ_C_ 24.1) indicated that they were both in an axial configuration. Indeed, in 4-(6-hydroxy-12-oxo-11(13)-eudesmen-4-yloxy)-11(13)-eudesmen-12,6-olide, a 4-*O*-4′ dimer between 4-*epi*-arbusculin A and its open form at the lactone, isolated from the liverwort *F. tamarisci* (Toyota et al., 1998), the axial methyl CH_3_-15 was observed at δ_C_ 22.7 and the equatorial CH_3_-15′ at δ_C_ 31.7. Finally, the multiplicity of H-1 (d, *J* = 10.4 Hz) has determined its axial position. Ft_5 was a new eudesmanolide dimer that was consequently named tamariscolide A.

This targeted isolation procedure was applied to the liverwort *P. porelloides* to yield various sesquiterpenes. Plagiochiline derivatives were isolated, which are characteristic compounds of the genus. Among them, plagiochiline D Pp_1 (Nagashima et al., 1994), plagiochiline *R*-15-yl octanoate Pp_2, and plagiochiline *R*-15-yl dec-4-enoate Pp_3 (Toyota et al., 1994) were previously described in this species, and plagiochiline *R*-15-yl hexanoate Pp_4 has not yet been described. Complete structural elucidation of Pp_4 was carried out by extensive NMR analysis; it should be noted that the NMR data of Pp_4 were similar to those of Pp_2 except for the lateral carbon chain. Complementarily, the eudesmanolide, diplophyllin Pp_5, previously described in the genus *Plagiochila* (Spörle et al., 1991), was also isolated.

Finally, the same chromatographic procedure applied to the liverwort, *P. epiphylla*, allowed the isolation of four perrottetin-type bis-bibenzyls already known in the species: 10′-hydroxyperrottetin E Pe_1, perrottetin E Pe_2, 10′-hydroxy-11-methoxy-perrottetin E Pe_3, and 11-methoxy-perrottetin E Pe_4 (Cullmann et al., 1997).

These 20 isolated fully characterized compounds, as well as 12 commercial standards and three compounds previously isolated from Japanese liverworts, were analyzed under the same conditions used for extract metabolomic profiling.

These 35 standards (25 terpenoids, including 18 sesquiterpenoids, three diterpenoids, and four triterpenoids, as well as five bis-bibenzyl stilbenoids, three flavonoids, and two new phenanthrenes) enabled a formal identification of the corresponding nodes and propagation of the annotation in 11 clusters in PI and three in NI. These structures, related to MS and NMR data, are summarized in **Supplementary Table S2** and **Supplementary S6**.

### 2.2 Chemical diversity in bryophytes

#### 2.2.1 Overview of distribution of chemical classes in mosses and liverworts

To obtain a good overview of the distribution of chemical superclasses in the different samples, the data were summarized in the form of a heatmap. The heatmap displays the sum intensities of all features corresponding to a given superclass (**Figure 2**, see Section 2.1.3).

The chemical superclasses were divided according to their pathway (alkaloids, fatty acids, terpenoids and shikimates, and phenylpropanoids). They are represented vertically and the species concerned horizontally according to their taxonomic proximity within two sections: liverworts in the upper part and mosses in the bottom part. A color gradient, from red to yellow in PI and dark blue to light blue in NI, represents the superclass according to their abundance.

The number of features detected by species, which is a measure of the extent of the chemodiversity, varies greatly from one species to another, independently of their phylum. These variations for the PI mode are represented in a bar plot in **Figure 2**. The species with the most features detected are the liverworts *P. arboris-vitae*, *Marsupella emarginata*, and *P. obtusata* with more than 1,461, 1,182, and 1,167 features, respectively, and they are followed by one moss, *Brachythecium rivulare*, with 990 features. Species with fewer features detected are *Pellia endiviifolia* and *Trichostomum crispulum* with only 18 and 43 features, respectively. This variation in detected features indicates an important variability among species and no real trend between phyla. Overall, SIRIUS was able to annotate more than 80% of the features (in light gray) with at least one chemical superclass.

The heatmap in the PI mode (**Figure 2**) differentiates between liverworts (upper panel) and mosses (lower panel). Liverworts are mainly characterized by the presence of abundant sesquiterpenoids. Within liverwort, however, most of the thallus liverworts displayed stilbenoids principally detected in the NI mode and, in general, fewer sesquiterpenoids.

The mosses are marked by the presence of fatty acids, which were also evidenced by the extraction yield during the cleaning process. Concerning terpenoids, and in comparison with liverworts, di- and tri-terpenoids were often detected. Sesquiterpenoids were also present but were, in general, less abundant. Mosses were also found to contain flavonoids mainly highlighted in the NI mode.

In general, compounds from the shikimate and phenylpropanoid pathway are poorly represented, with the exception of stilbenoids. Those are found in liverworts but were also surprisingly detected in three moss species of the family Polytrichaceae.

Remarkably, of all the samples, only one bryophyte, the liverwort *Riccia perrenis*, produced alkaloids and, at the same time, biosynthesized only a few terpenes.

More detailed information could be retrieved from the MN. In PI, the MN generated 370 clusters of more than two nodes and 4,368 single nodes (**Figure 1A**). Particular attention was paid to the main cluster, which generally groups together compounds of the same structural and indicates the main trend in chemical composition. The SIRIUS annotation shows that the first 16 clusters (first two lines of the MN) correspond to terpenoids (eight clusters: P1, P2, P3, P5, P9, P10, P11, and P12), fatty acids (three clusters: P4, P7, and P15), and stilbenoids (two clusters: P6 and P13). As suggested by the heatmap, the MN analysis shows that the most important clusters common to mosses and liverworts are annotated by SIRIUS as terpenoids (e.g., P1 and P9) and fatty acids (e.g., P4). In NI, the MN is made up of 4,604 nodes grouped into 190 clusters of more than two nodes and 2,245 single nodes (**Figure 1B**). In this mode, the annotations were less reliable, except for stilbenoids and flavonoids, which ionize better. MN reveals a few homogeneous clusters from the shikimate and phenylpropanoid pathway (e.g., N60) and some flavonoids (e.g., N9). The annotations for the other clusters were more heterogeneous and therefore difficult to use at this level.

#### 2.2.2 Investigation of selective chemical classes and taxonomical relationships

To obtain a more detailed view of the metabolite composition of the bryophyte species in the collection, the annotations will be analyzed by compound class, starting with those with the greatest number of features.These data will be presented in relation to the taxonomic relationships that exist between species, particularly between the two phyla, and will be discussed in greater detail in light of the published data in the Discussion section.

##### 2.2.2.1 Terpenoids

In the MN, analysis in the PI mode made it possible to associate 3,631 nodes with terpenoids (1,561 sesquiterpenoids, 643 diterpenoids, and 604 triterpenoids). Sesquiterpenoids are the largest group in liverworts, while di- and triterpenoids are the most represented groups in mosses. This can be seen on the heatmap in terms of intensity but also in terms of frequency of annotation in the MN.

Volatile compounds such as monoterpenoids are not discussed in this work.

###### 2.2.2.1.1 Sesquiterpenoids

Sesquiterpenes were associated with 1,561 nodes, which were mainly detected in liverworts (with 503 specific nodes), while only 70 were specific to mosses.

As shown in **Figure 3A**, the MN cluster P1 is the largest (211 nodes) and is mainly specific to the liverwort *P. porelloides*. The confidence of annotations in P1 was improved by an unambiguous identification of all nodes corresponding to the plagiochiline derivatives isolated from *P. porelloides*: plagiochiline D Pp_1, plagiochiline *R*-15-yl octanoate Pp_2, plagiochiline *R*-15-yl(4*Z*)-dec-4-enoate Pp_3 and a new derivative with a hexanoyl chain in C-15, plagiochiline *R*-15-yl hexanoate Pp_4.

While most nodes were only found in *P. porelloides*, two distant branches of P1 were shared by several species of moss and liverwort. It should be noted that some of these nodes are specific to the species *Thamnobryum alopecurum*, a moss for which no phytochemical studies are available.

Cluster P2 is composed of 168 nodes that were also mostly annotated as sesquiterpenoids, with the eudesmane, caryophyllene, and guaiane skeletons (**Figure 3B**). The annotations of this cluster were consolidated by HPLC–MS/MS data obtained on eight isolated components or standards: parthenolide St_4, costunolide St_5, dehydrocostus lactone St_6, diplophyllin Pp_5, telekin St_3, a new dimeric eudesmane sesquiterpene lactone (an isomer of muscicolide), and tamariscolide Ft_5 as well as frullanolide Ft_3, alantolactone St_1, and isoalantolactone St_2. These last three isomeric compounds however were coeluted and grouped under one node. This cluster was associated with sesquiterpenes of the γ-lactone type existing in the form of monomer or dimer as is the case for Ft_5, a dimer of γ-cyclocostunolide Ft_3. Among P2, 122 nodes are common to both phyla, and 40 are specific to liverworts while six to mosses. It is noticeable that the P8 cluster, which includes 51 nodes, brings together ions resulting from the in-source fragmentations of sesquiterpenes such as alantolactone St_1, frullanolide Ft_3, γ-cyclocostunolide Ft_4, and dehydrocostus lactone St_6. Among the sesquiterpene γ-lactones, the oxy-frullanolide Ft_2 was associated with cluster P12, holding 33 nodes annotated as sesquiterpenoids. It is noticeable that two other eudesmanolides, oxo-frullanolide Ft_1 and γ-cyclocostunolide Ft_4, were not linked to any cluster and appeared as single nodes when the precursor ion of the MS/MS was the [M+H]^+^. As mentioned, however, their in-source fragments are clustered in P8 (**Figure 3D**).

Cinnamolide St_8 was found in the P67 cluster, which holds several drimane-type sesquiterpene lactones. These compounds were identified in *P. arboris-vitae* and *P. obtusata*. However, cinnamolide St_8 and its derivatives were detected for the first time in mosses of the genus: *Dicranale*, *Hedwigiale*, *Bryale*, *Polytrichale*, *Hypnale*, *Grimmiale Bartramiale* and *Polytrichale* and in liverworts of order: *Marchantiale* and *Jungermanniale* (**Figure 3I**).

###### 2.2.2.1.2 Di- and triterpenoids

Several clusters (P9, P33, P37, etc.) were associated with diterpenoids. Only P179, a small cluster including only four nodes that were shared by both mosses and liverworts, was confirmed by a standard clerod-3,13(16),14-trien-17-oic acid St_9 (**Figure 3J**).

Despite the high similarity of betulin St_12 and its acid St_13, two lupane derivatives, only betulin St_12 was associated with cluster P10. This cluster comprises 47 nodes mainly annotated as lupane-type triterpenoids (**Figure 3E**). In PI, St_13 appears in a single node while in a very small cluster (two nodes) in NI with ursolic acid Ft_6, another triterpenoid. The MN showed that P10 was common with mosses and liverworts, while betulin has been previously described only in the liverwort, *P. epiphylla*, and betulinic acid in two mosses, *Heteroscyphus coalitus* and *Ptilidium pulcherrimum*.

Tamariscolide Ft_5 isolated from *F. tamarisci* is mislabeled as a triterpenoid on the basis of its crude formula and was finally identified as a dimer of γ-cyclocostunolide, a lactone sesquiterpene. Ft_5 shares the P2 cluster associated with monomeric or dimeric sesquiterpene lactones.

##### 2.2.2.2 Shikimate and phenylpropanoids

###### 2.2.2.2.1 Stilbenoids

**Figure 5:**
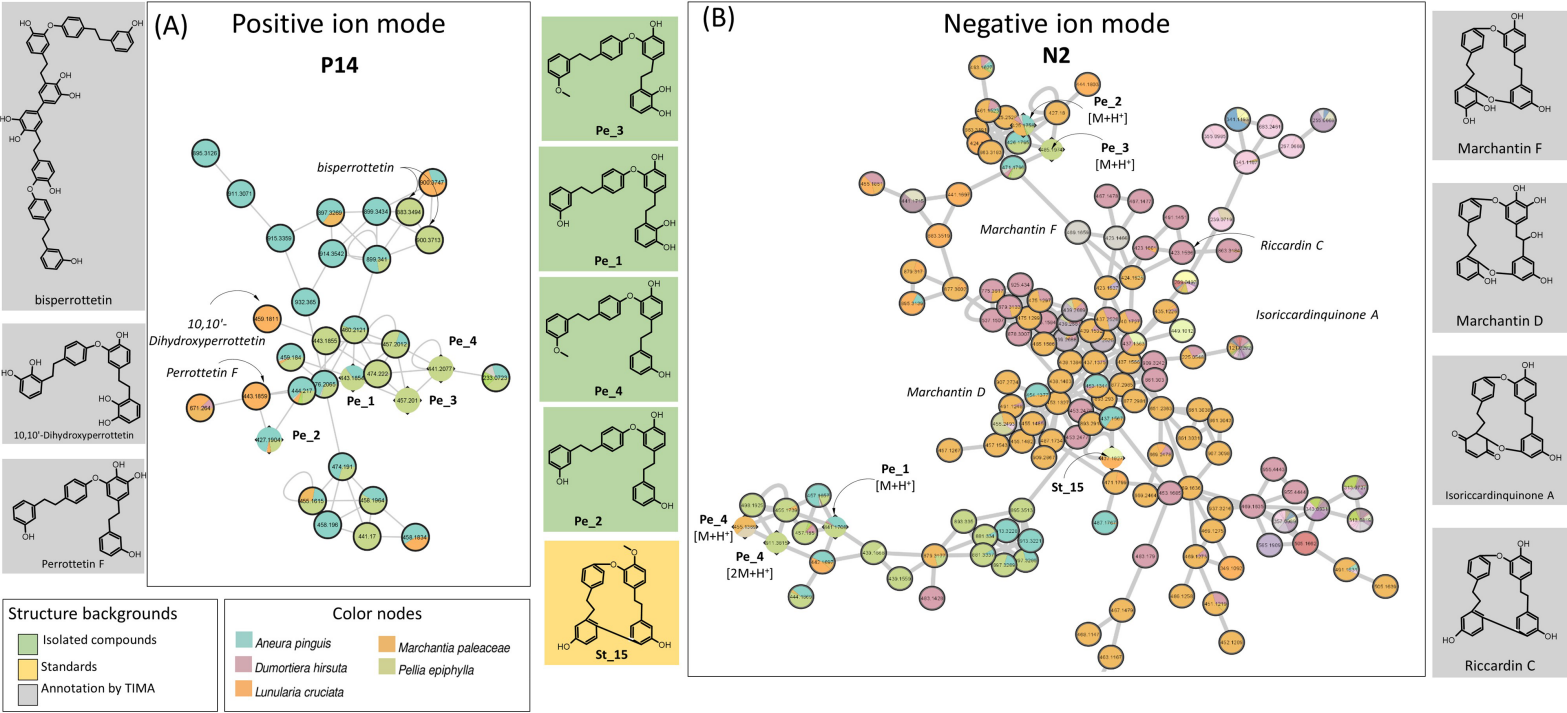
Confirmed cluster of perrottetin derivatives **(A)** in PI (P14) and **(B)** NI (N2). Confirmed annotations appear on a green background (isolated compounds) or an orange background (standard compounds), and selected annotations from TIMA tools appear on gray background. PI, positive ionization; NI, negative ionization; TIMA, Taxonomically Informed Metabolite Annotation.

In PI, the MN shows 800 nodes annotated to shikimates and phenylpropanoids, and among them, 113 were stilbenoids. The annotations of cluster P14 were ensured by four bis(bibenzyl)-type perrottetin derivatives (Pe_1, Pe_2, Pe_3, and Pe_4) isolated from *P. epiphylla* (**Figures 3G**, **5A**). This cluster included mainly nodes with molecular weights between 427.19 and 476.20 corresponding to perrottetin derivatives but also other nodes with molecular weights of 800 or higher, suggesting perrottetin dimers. It is noticeable that cluster P14 was mainly shared by four thalloid liverworts: *P. epiphylla*, *Lunularia cruciata*, *Marchantia paleacea*, and *Aneura pinguis*. The perrottetin derivatives have been previously described in these species, except for *A. pinguis*, for which no data were available in the literature.

The MS signal in NI of stilbenoids was better than in PI, as clearly seen on the heatmaps and MN (**Figures 1B**, **2B**). A large N2 cluster grouped perrottetin derivatives from *P. epiphylla* (Pe_1, Pe_2, Pe_3, and Pe_4) and riccardin G St_15, which appears as a single node in PI (**Figure 5B**). Suggesting marchantin-type compounds, such as marchantin A, C, G, H, and M, marchantinquinone, and riccardin C and F, the TIMA annotations were highly relevant in this case. In addition, analysis of the UV spectra of these compounds showed absorbance between 220 and 307 nm, confirming the presence of molecules with similar frameworks.

Cluster N5 was annotated as alkaloids, and shikimates and phenylpropanoids, with annotation probabilities between 0.8 and 0.5. N5 displayed 63 nodes mainly specific to *Bazzania japonica*. The SIRIUS annotations were not reliable, while TIMA proposed several chlorinated macrocyclic bis-bibenzyls belonging to bazzanin with a good final score. TIMA annotations appear to be reliable because these compounds were specific to the rare and little-studied *Bazzania* genus (Martini et al., 1998; Scher et al., 2003). In addition, analysis of the isotopic pattern confirms the presence of one or more chlorines, depending on the molecule.

###### 2.2.2.2.2 Flavonoids

**Figure 6:**
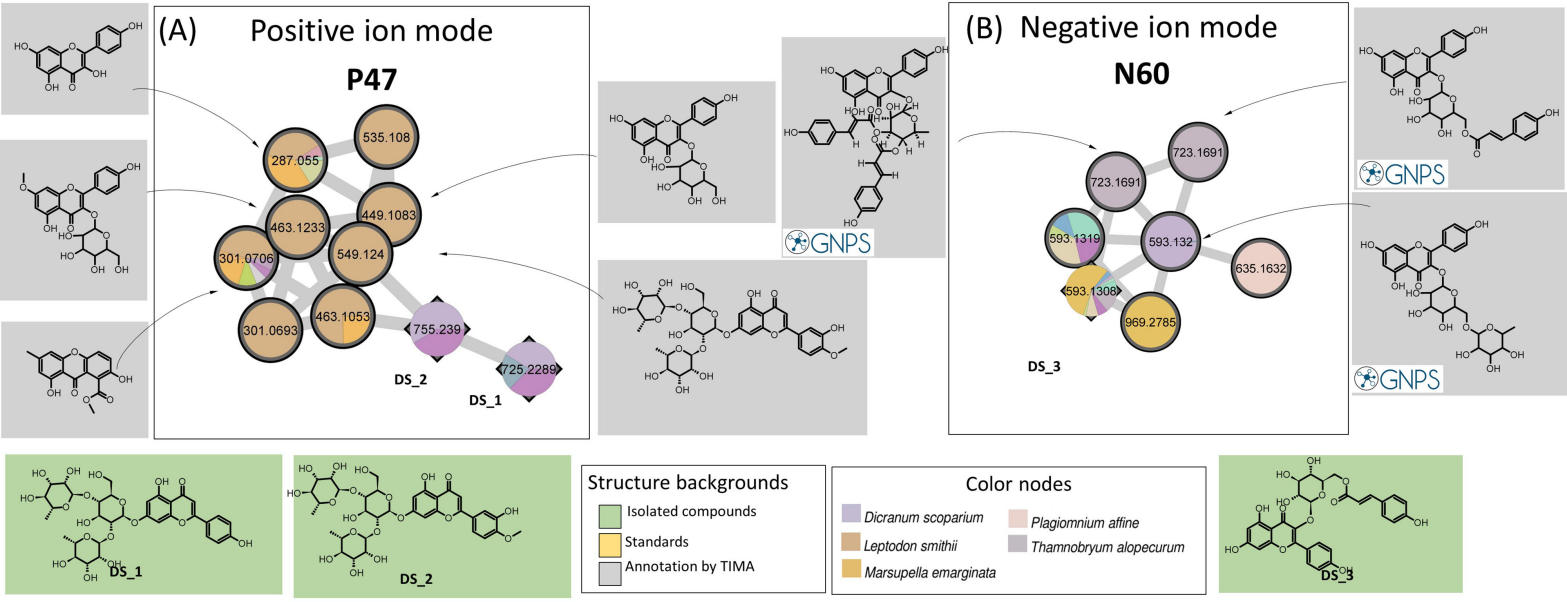
Confirmed cluster of flavonoid derivatives **(A)** in PI (P47) and **(B)** NI (N60). Confirmed annotations appear on a green background (isolated compounds) or an orange background (standard compounds), and selected annotations from TIMA tools appear on gray background. PI, positive ionization; NI, negative ionization; TIMA, Taxonomically Informed Metabolite Annotation.

The isolation procedure carried out in the present study enabled us to purify three flavanones: Ds_1, Ds_2, and Ds_3. In positive MN, Ds_1 and Ds_2 belong to a small P47 cluster (10 nodes). The TIMA tools provide seven annotations, including five glycosylated kaempferols (**Figure 6A**). The two triglycosylated apigenins, Ds_1 and Ds_2, were the most intense in *D. scoparium*, while the other nodes were detected in the moss *Leptodon smithii* and mainly annotated to kaempferol derivatives. As noted, the chemical composition of the moss *L. smithii* has never been studied. Ds_3, a kaempferol derivative, has a very weak signal in PI but belongs to a small N60 cluster (seven nodes) in NI (**Figure 6B**). For N60, GNPS offers annotations corresponding to three kaempferol derivatives, which were ensured by absorptions at 254–280-nm and 340–360-nm characteristics to flavones and flavonols in the UV spectra. Among these nodes, two are specific to *T. alopecurum*, one to *Plagiomnium affine*, and one to *M. emarginata*. For these three species, flavonoids have never been reported in the literature, and, in particular, no data are available for *T. alopecurum*. However, as reported in the literature (Asakawa et al., 2013a), triterpenoid compounds from *P. affine* and sesquiterpenoids from *M. emarginata* were detected in our study, but only in weak ions.

###### 2.2.2.2.3 Phenanthrenes

**Figure 7:**
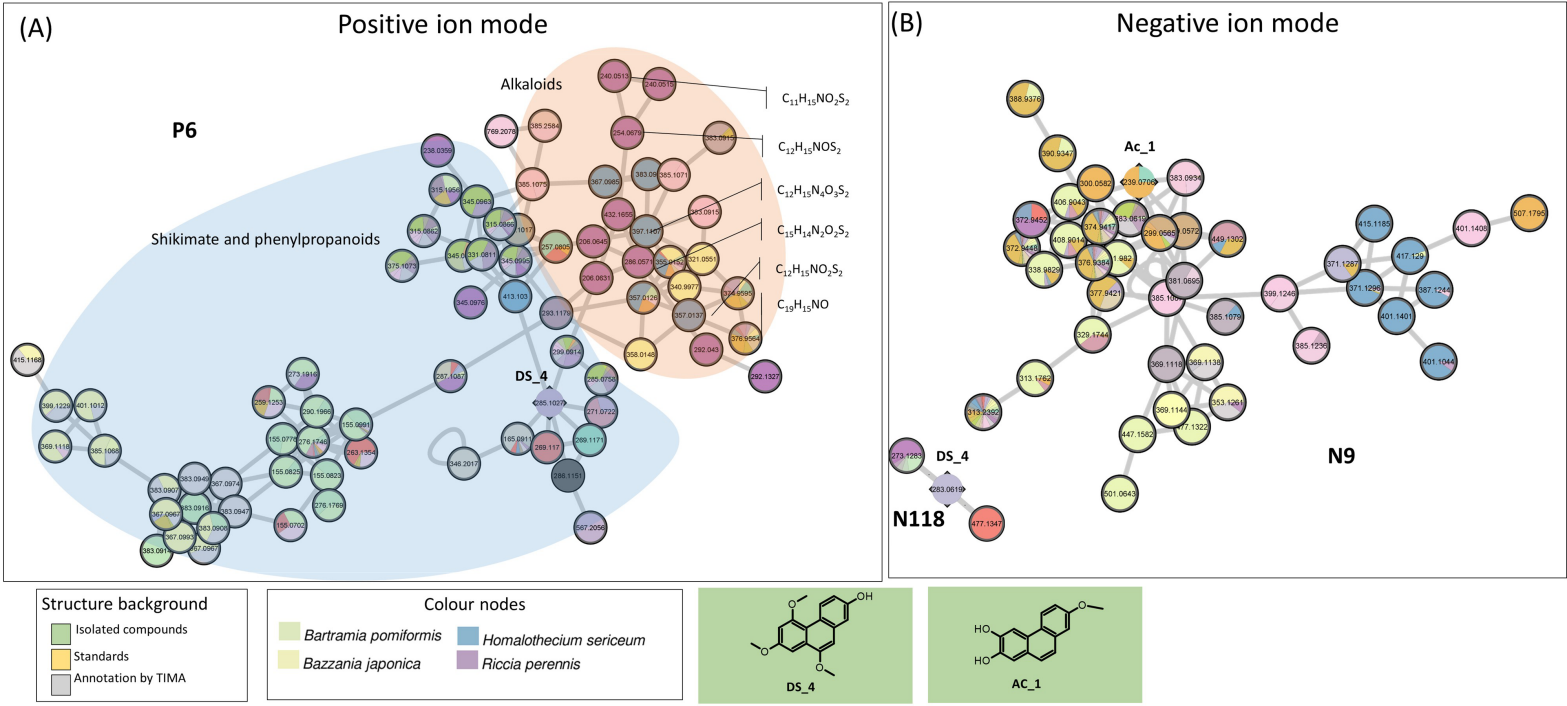
**(A)** Cluster sharing of phenanthrene and alkaloids in PI (P6). Nodes annotated with shikimates and phenylpropanoids by NPClassifier on blue background and with alkaloids on orange background. **(B)** Validated cluster of phenanthrene derivatives in NI (N118 and N9). Confirmed annotations appear on a green background (isolated compounds). PI, positive ionization; NI, negative ionization.

In PI, the large P6 cluster is partly annotated as phenanthrenes. One of these nodes corresponds to Ds_4, a new phenanthrene derivative isolated from *D. scoparium* (**Figures 3C**, **7A**). In negative mode, Ds_4 validates the little N118 cluster (three nodes), which includes three specific nodes to the mosses *T. alopecurum*, *P. affine*, and *D. scoparium* and one to the liverwort *M. emarginata*. The undescribed phenanthrene Ac_1 isolated from *A. curtipendula* displayed cluster N9 (43 nodes), which indiscriminately represents liverworts and mosses. Within cluster N9, most nodes were assigned to several species, but a few nodes were specific to *T. alopecurum*, *M. paleacea*, and *B. japonica*. For the chemical class phenanthrenes, the SIRIUS and TIMA annotations proved to be unreliable (**Figure 7B**).

##### 2.2.2.3 Alkaloids

Nitrogenated metabolites are rare in bryophytes. Our metabolomic approach shows that 2.5% of 8,443 nodes (217) were annotated as alkaloids with a higher probability (confidence score of 0.8). Among them, 59 and 99 were specific to mosses and liverworts, respectively. Alkaloids shared the P6 cluster with phenanthrenes, so they do not form a specific cluster; however, with 13 nodes under 77, they represented a homogenous part of P6 (**Figure 7A**). Among those, nine nodes are specific to the liverwort *Riccia perennis*; they correspond to the major peaks observed in Charged Aerosol Detector (CAD). This trend was confirmed in the heatmap (**Figure 3**). It is noteworthy that five nodes of the P6 cluster displayed a molecular formula containing one nitrogen and one or two sulfurs like the compounds identified from the liverwort *Corsinia coriandrina* (von Reuß and König, 2004, 2005). Inspection of the isotopic pattern of these nodes confirms the presence of one or more sulfur atoms, depending on the case. These compounds also show a base peak at *m/z* 206.06 reported in the *O*-methyltridentatols of *C. coriandrina*. However, further analysis is required to confirm compound annotations of the cluster.

## 3 Discussion

The study is the first to investigate a large collection of bryophyte extracts under the same analytical conditions. These samples represent 60 species, 15 orders, and 41 families, i.e., 1/3 of French bryophyte orders and 10% of all Corsican bryophyte species. The systematic study of the metabolomes of this representative collection has made it possible, for the first time, to determine the full range of chemotaxonomic relationships within the mosses and liverworts and between these two phyla.

Mosses are understudied in the literature because they are mainly composed of fatty acids (Asakawa et al., 2013a).

The secondary metabolite enrichment procedure developed in this study proved to be an effective solution for overcoming this problem and improving the detection of target compounds.

The loss of mass of raw moss extracts is 72% on average, whereas it is only 52.5% for liverwort extracts.

Although fatty acids remained dominant in the moss extracts, di- and triterpenoids were detected as an important chemical superclass, except for three mosses belonging to the family Polytrichaceae (*Pogonatum urnigerum*, *Polytrichum juniperium*, and *Polytrichum piliferum*), which produce flavonoids and stilbenoids. In the literature, mosses produce small quantities of flavonoids and very few stilbenoids, and the presence of large quantities of flavonoids and stilbenoids in this family makes it unique.

According to the literature (Asakawa et al., 2013a), liverworts produce many sesquiterpenoids. Our data confirm the high proportion of sesquiterpenes in this phylum. They distinguish thallus liverworts, characterized by a high proportion of stilbenoids, from leaf liverworts, which tend to produce more sesquiterpenoids. The sesquiterpenoids have formed homogeneous and specific clusters in the MN.

Among them, a large P1 cluster was found to be almost unique to the hepatic *P. porelloides* and encompasses numerous plagiochiline derivatives for which four (Pp_1, Pp_2, Pp_3, and Pp_4) were unambiguously identified after isolation.

Plagiochiline derivatives have recently been reported for their cytotoxic activities (Vergoten and Bailly, 2023). The richness of P1 indicates a possible discovery of additional bioactive plagiochilines. As more than 1,600 liverworts of the genus have been reported, they can be considered a good source for such compounds.

A large homogeneous cluster P2 was assigned to the γ-sesquiterpene lactones, which included many species of liverworts and mosses. Sesquiterpene lactones are known for their diverse biological activities, as potent herbicides, insecticides, anticancer agents, and antifoulants, and for their wide applicability in the food and perfume industries (Sartori et al., 2021). The literature describes the presence of active γ-lactones preferentially in liverworts. Our MN analysis showed clusters of γ-lactones associated with both phyla, opening new avenues of investigation.

In the thallus liverworts, the main chemical superclass was stilbenoid, in good agreement with the literature (Asakawa et al., 2021). In our data, clusters P14 and N2 were validated with five isolated compounds corresponding to the bis-bibenzyl stilbenoids, which are known to have significant biological activities. They were both homogenous to the thallus liverworts. Among the species, the occurrence of bis-bibenzyl stilbenoids in *L. cruciata* and *M. paleacea* is well known; however, *A. pinguis* appears to be a high potential species for finding novel bis-bibenzyl stilbenoids (numerous specific nodes).

Finally, the liverwort *R. perennis* appears as a very original species because of the presence of alkaloids. This result is remarkable given the scarcity of nitrogen compounds described in bryophytes. Only nine alkaloids were reported in bryophytes. Their presence in *R. perennis* is described here for the first time. Several of our annotations corresponded to alkaloids that also contain sulfur, in which the proposed molecular formula does not correspond to any nitrogen–sulfur compounds already described in bryophytes. Sulfur alkaloids are common in marine organisms and have shown a wide variety of biological activities: cytotoxicity, antiviral, anti-inflammatory, and antioxidant (Zhang et al., 2023). In the bryophytes, only isotachin A, isotachin B, coriandrins, and methyl tridentatols, four sulfur-containing alkaloids, are known in very rare thallus liverworts (von Reuß and König, 2004, 2005). Consequently, the *Riccia* genus, which includes 19 species in Corsica, appears as a promising reserve of sulfur-containing alkaloids.

The present article shows an initial chemical mapping of the library of the bryoflora extracts, supported by important clusters validated with pure compounds and focusing on species with high interest. Our metabolomics innovative approach has produced a large dataset that could be the subject of more detailed work, as well as an approach targeting biological active ingredients.

A great deal of information is still unexploited and will be the subject of future work. All these data are available on the GNPS platform so that everyone can approach the chemistry of bryophytes in a new way and advance the knowledge of these plants whose full potential is not yet known.

## 4 Materials and methods

### 4.1 Plant materials

#### 4.1.1 Harvesting

Samples of the 60 bryophytes were collected at random locations in Corsica. Sampling was performed in 2021. Botanical determination was performed according to the botanical determination keys summarized in Bryophyte Flora (Augier, 1966) by Achille Pioli, a specialist in mosses, and voucher specimens were deposited in the herbarium of the University of Corsica, Corte (France). Three Japanese liverworts collected as part of a collaboration with the Tokushima Bunri University, Japan, were added to the collection in Tokushima. The sample numbers, the geographical origin of the different samples, and the voucher codes for each specimen analyzed are listed in **Supplementary Table S1**. All samples were harvested in the wet season of 2021 (January–April and September–December 2021).

#### 4.1.2 Extractions

After 15 days of drying at room temperature, plant material was powdered by cryo-grinding and successively extracted with hexane, methylene chloride, and methanol for 24 h each. Solvent volume corresponded to v = mass of sample × 100. The extracts were dried using a rotary evaporator or lyophilization. The masses and the yields obtained are summarized in **Supplementary Table S1**.

### 4.2 Cleaning of extracts

To reduce the amount of apolar compounds, such as fatty acids, and polar compounds such as saccharides, the extracts were cleaned by sample clean-up before HRMS[electrospray ionization (ESI)]–MS analysis and sample enrichment before the isolation step. This protocol corresponds to **Supplementary Figure S2**.

#### 4.2.1 Sample clean-up

The solid-phase extraction procedure used C18 cartridges (50 µm, 12 mL, 1,000 mg; Finisterre, Teknokroma, Spain) in conjunction with a Teknokroma extraction manifold system (12-position manifold with a 13 × 75 mm test tube rack). The vacuum pressure on the manifold was maintained at ≤5 inches (12.7 mmHg) throughout the duration of the SPE protocol. The cartridges were conditioned using 10 mL of methanol–water (50:50) and 10 mL of pure methanol.

A sample with a mass of 50 mg was dissolved in 5 mL of mixture MeOH:H_2_O 95:5. Analytes were eluted with 10 mL methanol–water (95:5). Eluates were evaporated using Genevac. The samples were reconstituted in methanol at 5 mg·mL^−1^, and they were transferred into a microplate for LC–MS/MS analysis.

#### 4.2.2 Sample enrichment

The selected extracts were dissolved in a mixture of MeOH/H_2_O (7:3) at 5 mg·mL^−1^. They were extracted by liquid–liquid extraction (LLE) with hexane to equal volume. The two phases were separated, dried, and evaporated. The extract obtained from the organic phase (MeOH/H_2_O) was dissolved in butanol at 5 mg·mL^−1^ and extracted by LLE with water to equal volume. After drying and evaporation, the extract obtained from the organic phase was cleaned by SPE.

SPE C18-cartridges (50 µm, 12 mL, 1,000 mg; Finisterre, Teknokroma, Spain) were conditioned with a 10-mL mixture of methanol–water (50:50) and 10 mL water pure. Samples were mixed with deactivated silica and disposed of on the head of the SPE cartridge after conditioning. Samples passed into the cartridges at a flow rate of approximately 5 mL/min under vacuum. Analytes were eluted successively with 10 mL of methanol–water (90:10), 10 mL of water, and 10 mL of ethyl acetate. Eluates were evaporated using Genevac.

### 4.3 Mass spectrometry analysis

#### 4.3.1 UHPLC–PDA–ELSD–(Q)MS metabolite profiling

Analysis and chromatographic data were obtained on an ultra-high-performance liquid chromatography system equipped with a photodiode array, an evaporative light-scattering detector, and a single quadrupole detector using heated electrospray ionization (UHPLC–PDA–ELSD–QDA) (Waters, Milford, MA, USA). The ESI parameters were as follows: capillary voltage 800 V, cone voltage 15 V, source temperature 120°C, and probe temperature 600°C. The acquisition was performed in positive ionization mode with an *m/z* range of 150–1,000 Da. The chromatographic separation was performed on an Acquity UPLC BEH C18 column (50 × 2.1 mm i.d., 1.7 μm; Waters) at 0.6 mL/min, 40°C with H_2_O (A) and MeCN (B), both containing 0.1% formic acid as solvents. The gradient was carried out as follows: 5%–100% B in 7 min, 1 min at 100% B, and a re-equilibration step at 5% B for 2 min. The ELSD temperature was fixed at 45°C, with a gain of 9. The PDA data were acquired from 190 to 500 nm, with a resolution of 1.2 nm. The sampling rate was set at 20 points/s.

#### 4.3.2 UHPLC–HRMS/MS metabolite profiling

Analysis, data processing, and feature-based molecular network generation chromatographic data with high-resolution MS were obtained on a Waters Acquity UHPLC system equipped with a Q-Exactive Focus mass spectrometer (Thermo Scientific, Bremen, Germany), using heated electrospray ionization source (HESI-II). The chromatographic separation was carried out on an Acquity UPLC BEH C18 column (50 × 2.1 mm i.d., 1.7 μm; Waters) at 0.6 mL/min, 40°C with H_2_O (A) and MeCN (B), both containing 0.1% formic acid as solvents. The gradient was carried out as follows: 5%–100% B in 7 min, 1 min at 100% B, and a re-equilibration step at 5% B in 2 min. The ionization parameters were the same as those used in Rutz et al. (2019).

#### 4.3.3 UHPLC–HRMS/MS data processing

The raw UHPLC–HRMS/MS files were converted into mzXML files using the MSConvert software. The mzXML files were then processed using the open software MZmine (3.4.16) (Schmid et al., 2023). The mass detection step was performed using a centroid mass detector with a noise level set at 1E^6^ for MS1 and 1E^4^ for MS^2^ in PI and 1E^4^ for MS1 and 1E^4^ in MS^2^ in NI. The ADAP chromatogram builder was employed with a minimum group size of scans of 4, a minimum group intensity threshold of 1E^6^, a minimum highest intensity of 1E^6^ in PI and 5E^4^ in NI, and an *m/z* tolerance of 10 ppm. The deconvolution was carried out with the ADAP (Wavelets) algorithm, using a signal-to-noise threshold of 50, a minimum feature height of 1E^6^, a coefficient/area threshold of 100, a peak duration range of 0.01–0.9 min, and a wavelet range between 0.01 and 0.08 min. The *m/z* and retention time (RT) for MS^2^ scan pairing were, respectively, set to 0.005 Da and 0.1 min. The isotopes were grouped using the isotope peak grouper algorithm with an *m/z* tolerance of 3 ppm, an RT tolerance of 0.05 min, and a maximum charge of 2, using the most intense isotope as the representative one. The alignment was carried out with the join aligner with an *m/z* tolerance of 15 ppm, an RT tolerance of 0.1 min, and a weight tolerance for *m/z* and RT of 10 each. Ion identity networking parameters were set to *m/z* tolerance, 0.002 *m/z* or 5 ppm; check, one feature; min height, 1E^3^ with ion identity library parameters set to MS mode, positive; maximum charge, 2; maximum molecules/cluster, 2; adducts, M+H, M+Na, M+K; modifications, M–H_2_O, M–NH_3_.

#### 4.3.4 Molecular network generation

The MZmine aligned table was exported in MGF format for the processing of the Feature-based Molecular Networking (FBMN). The spectral data were uploaded on the GNPS platform (Nothias et al., 2020). A network was generated with a minimum cosine score of 0.85 and a minimum of five matching peaks. The experimental spectra were searched against GNPS’s spectral libraries. The obtained network was visualized in the software Cytoscape (3.9.1, Institute for Systems Biology, Seattle, WA, USA) (Smoot et al., 2011).

The mass spectrometry data were deposited on the MassIVE public repository nos. MSV000093186 (PI) and MSV000093188 (NI) with different GNPS job parameters, and resulting data are available at the following addresses:

PI network: ID=895c9f23e6df4c30a42d8774af4121c1.

NI network: ID=5e783c91e5e74ed9aee07cc59fc12830.

#### 4.3.5 Class annotation for the dataset

Filtered features detected in PI and NI in the general dataset were annotated using a computational approach integrating SIRIUS (molecular formula) (Ludwig et al., 2020), CSI:fingerID (probabilistic molecular fingerprint by machine learning substructure prediction and *in silico* annotation) (Dührkop et al., 2015), and CANOPUS (systematic class annotation) (Dührkop et al., 2019, 2021). In the second step, the spectral file and attribute metadata obtained after the MN step were annotated by matching the MS1 and MS^2^ spectra data with the LOTUS-ISDB [in-house database containing the *in silico* fragmentation spectra of all the compounds present in the Dictionary of Natural Products (DNP) and LOTUS databases] complemented with structure–organism pairs coming from the DNP (Allard et al., 2016; Rutz et al., 2019). The following parameters were used: spectral match parameters: parent mass tolerance 0.01 Da, MS/MS tolerance 0.01 Da, minimum cosine score 0.2, and minimum peaks 6. Spectral match of MS/MS spectra against the database provided a list of 50 chemical structure candidates for every feature. The candidates were re-ranked by taxonomic reweighting after ponderation of their spectral score inversely proportional to the taxonomic distance between the biological source of the candidate and that of the one-off analyzed sample(s) in which the feature is detected.

#### 4.3.6 Chromatographic optimization and semi-preparative HPLC–UV isolation

The separation conditions of the DCM or MeOH extracts were optimized on an HP 1260 Agilent high-performance liquid chromatography equipped with a photodiode array detector and an ELSD detector (HPLC–PDA–ELSD) (Agilent Technologies, Santa Clara, CA, USA). The chromatographic separation was performed on an XBridge C18 column (250 × 4.6 mm i.d., 5 μm; Waters) equipped with a C18 pre-column at 1 mL/min, with H_2_O (A) and MeCN (B), both containing 0.1% formic acid as solvents. The UV absorbance was measured at 280 and 360 nm, and UV–Vis spectra were recorded between 190 and 600 nm (step 2 nm). The optimized gradient used for the *F. tamarisci* DCM extracts and *P. porelloides* MeOH extracts was as follows: 5 mn at 30% B, 30%–100% B in 45 min, and 10 min at 100% B. The optimized gradient *P. epiphylla* DCM extract used for the was as follows: 5 mn at 35% B, 35%–100% B in 45 min, and 10 min at 100% B. The optimized gradient *A. curtipendula* MeOH extract used for the was as follows: 5 mn at 15% B, 15%–75% B in 45 min, and 10 min at 100% B. The optimized gradient *D. scoparium* MeOH extract used for the was as follows: 5 mn at 15% B, 15%–75% B in 45 min, and 10 min at 100% B.

These chromatographic methods were geometrically transferred (Guillarme et al., 2008) to the semi-preparative scale on a Shimadzu system equipped with an LC-20, module pumps, an SPD-20 A UV/VIS, a 7725I Rheodyne^®^ valve, and an FRC-40 fraction collector (Shimadzu, Kyoto, Japan). The separation was performed on an XBridge C18 column (250 mm × 19 mm i.d., 5 μm; Waters) equipped with a C18 pre-column cartridge holder (10 mm × 19 mm i.d., 5 μm; Waters) at 17 mL/min, with H_2_O (A) and MeCN (B) both containing 0.1% formic acid as solvents. The UV detection was set at 280 and 360 nm. The mixtures were injected into the semi-preparative HPLC column using a dry-load methodology developed in our laboratory (Queiroz et al., 2019). Masses injected and fractionation details are summarized in **Supplementary Figure S5**.

#### 4.3.7 NMR conditions

A Bruker Avance Neo 600 MHz NMR spectrometer equipped with a QCI 5 mm Cryoprobe and a SampleJet automated sample changer (Bruker BioSpin, Rheinstetten, Germany) was employed for 1D-NMR (^1^H and ^13^C-NMR) and 2D-NMR [correlation spectroscopy (COSY), multiplicity editing heteronuclear single-quantum correlation (edited-HSQC), HMBC, ROESY, and total correlation spectroscopy (TOCSY)] spectroscopy. Chemical shifts are reported in parts per million (δ) using the residual solvent signal at δ_H_ 7.26; δ_C_ 77.2 for CDCl_3_, δ_H_ 3.31; δ_C_ 49.0 for CD_3_OD and δ_H_ 2.50; and δ_C_ 39.5 for DMSO-*d*_6_. Chemical shifts (*J*) are reported in Hz.

#### 4.3.8 Standard compounds

Metabolite standards were purchased from Biopurify (Chengdu, China): alantolactone St_1, isoalantolactone St_2, parthenolide St_4, costunolide St_5, dehydrocostus lactone St_6, artemisinin St_7, cinnamolide St_8, tanshinone I St_10 and IV St_11, betulin St_12, betulinic acid St_13, and celastrol St_14. Telekin St_3, clerod-3,13(16),14-trien-17-oic acid St_9, and riccardin G St_15 were isolated by Prof. Nagashima and Prof. Asakawa from Japan liverworts.

#### 4.3.9 Description of the isolated compounds

The NMR descriptions of known compounds (Ft_2, Ft_3, Ft_4, Ft_6, Pp_1, Pp_2, Pp_3, Pp_5, Pe_1, Pe_2, Pe_3, Pe_5, Ds_1, Ds_2, and Ds_3) are summarized in the **Supplementary Material**. All spectra are according to the literature. The NMR spectra of unknown compounds (Ft_1, Ft_5, Pp_4, Ds_4, and Ac_1) are summarized in the **Supplementary Material** (**Supplementary Figures S7**-**S33**).

Six compounds were obtained from *F. tamarisci* (the yields for each compound are the sum of the compound isolated from the two extracts) in DCM extract from 20 mg [Ft_1 (1 mg)] and in MeOH Extracts Ft_2 (2.8 mg), Ft_3 (6.1 mg), Ft_4 (6.2 mg), Ft_5 (3.9 mg), and Ft_6 (3 mg)].

Ft_1: oxo-frullanolide ^1^H NMR (CDCl_3_, 600 MHz) δ 1.28 (3H, s, H_3_-14), 1.42 (1H, overlapped, H-9ax), 1.60 (1H, overlapped, H-9eq), 1.68 (1H, overlapped, H-8ax), 1.70 (1H, overlapped, H-1eq), 1.80 (1H, overlapped, H-8eq), 1.90 (3H, s, H-15), 1.97 (1H, td, *J* = 14.2, 13.7, 5.1 Hz, H-1ax), 2.52 (1H, dt, *J* = 12.7, 5.1 Hz, H-2eq), 2.73 (1H, m, H-2ax), 3.17 (1H, td, *J* = 7.2, 6.4 Hz, H-7), 5.37 (1H, d, *J* = 6.4 Hz, H-6), 5.71 (1H, s, H-13″), 6.30 (1H, s, H-13′); ^13^C NMR (CDCl_3_, 151 MHz) δ 11.1 (CH_3_-15), 24.7 (CH_3_-14), 25.4 (CH_2_-8), 34.2 (CH_2_-2), 34.5 (C-10), 36.5 (CH_2_-9), 37.5 (CH_2_-1), 40.6 (CH-7), 75.6 (CH-6), 122.6 (CH_2_-13), 136.8 (C-4), 140.5 (C-11), 151.7 (C-5), 170.3 (C-12), 198.8 (C-3); HR-ESI/MS, see **Supplementary Table S2**.

Ft_2: oxy-frullanolide (Sangsopha et al., 2016) HR-ESI/MS, see **Supplementary Table S2**; ^1^H and ^13^C NMR data, see **Supplementary Material S1**.

Ft_3: frullanolide (Chou and Liao, 2013) HR-ESI/MS, see **Supplementary Table S2**; ^1^H and ^13^C NMR data, see **Supplementary Material S1**.

Ft_4: γ-cyclocostunolide (Kraut et al., 1994) HR-ESI/MS, see **Supplementary Table S2**; ^1^H and ^13^C NMR data, see **Supplementary Material S1**.

Ft_5:tamariscolide ^1^H NMR (CDCl_3_, 600 MHz) δ 1.09 (3H, s, H_3_-14), 1.10 (3H, s, H_3_-14′), 1.12 (1H, overlapped, H-1′b), 1.30 (3H, s, H_3_-15), 1.31 (3H, s, H_3_-15′), 1.37 (1H, m, H1′-a), 1.41 (2H, m, H_2_-9′), 1.47 (4H, m, H-2′b, H-2b, H-3′a, H-8b), 1.53 (1H, m, H-3′b), 1.59 (1H, m, H-2′a), 1.63 (2H, m, H-3b, H-8′b), 1.70 (1H, m, H-9b), 1.77 (1H, m, H-2a), 1.80 (1H, m, H-3a), 1.94 (1H, d, *J* = 10.5 Hz, H-5′), 1.98 (1H, m, H-8′a), 2.00 (1H, m, H-5), 2.05 (1H, d, *J* = 13.4 Hz, H-8a), 2.12 (1H, d, *J* = 10.4 Hz, H-1), 2.27 (1H, d, *J* = 12.2 Hz, H-9a), 2.64 (2H, brs, H-7, H-7′), 4.05 (2H, m, H-6, H-6′), 5.38 (1H, s, H-13′b), 5.43 (1H, s, H-13b), 6.08 (1H, s, H-13′a), 6.10 (1H, s, H-13a); ^13^C NMR (CDCl_3_, 151 MHz) δ 18.3 (CH_2_-2′), 19.2 (CH_3_-14), 21.6 (CH_3_-14′), 22.5 (CH_2_-8′), 22.7 (CH_2_-8), 23.1 (CH_2_-2), 24.1 (CH_3_-15′), 24.7 (CH_3_-15), 35.0 (CH_2_-3′), 38.3 (C-10′), 40.8 (CH_2_-3), 41.0 (CH_2_-1′), 42.0 (CH_2_-9), 42.5 (C-4′), 45.2 (CH_2_-9′), 45.4 (C-10), 49.6 (CH-7), 51.4 (CH-7′), 53.4 (CH-5′), 55.8 (CH-1), 59.8 (CH-5), 71.8 (C-4), 82.2 (CH-6′), 82.4 (CH-6), 117.3 (CH_2_-13′), 118.1 (CH_2_-13), 138.7 (C-11), 139.7 (C-11′), 169.8 (C-12), 170.3 (C-12′); HR-ESI/MS, see **Supplementary Table S2**.

Ft_6:ursolic acid (Acebey-Castellon et al., 2011): HR-ESI/MS, see **Supplementary Table S2**; ^1^H and ^13^C NMR data, see **Supplementary Material S1**.

Five compounds were obtained from *P. porelloides*: (the yields for each compound are the sum of the compound isolated from the two extracts) in MeOH extract from 100 mg: Pp_1 (9.2 mg), Pp_2 (3.4 mg), Pp_3 (3.6 mg), Pp_4 (3.6 mg), and Pp_5 (1.3 mg).

Pp_1: plagiochiline D (Asakawa et al., 1979): HR-ESI/MS see **Supplementary Table S2**; ^1^H and ^13^C NMR data, see **Supplementary Material S1**.

Pp_2: plagiochiline *R*-15-yl octanoate (Toyota et al., 1994; Ramírez et al., 2017): HR-ESI/MS, see **Supplementary Table S2**; ^1^H and ^13^C NMR data, see **Supplementary Material S1**.

Pp_3: plagiochiline *R*-15-yl dec-4-enoate (Toyota et al., 1994; Ramírez et al., 2017): HR-ESI/MS, see **Supplementary Table S2**; ^1^H and ^13^C NMR data, see **Supplementary Material S1**.

Pp_4: plagiochiline *R*-15-yl hexanoate ^1^H NMR (DMSO-*d*_6_, 600 MHz) δ 0.85 (3H, t, *J* = 7.1 Hz, H_3_-6′), 0.99 (1H, t, *J* = 10.0 Hz, H-6), 1.07 (1H, m, H-9α), 1.20 (1H, m, H-8α), 1.25 (4H, m, H_2_-5′, H_2_-4′), 1.30 (1H, m, H-7), 1.51 (2H, p, *J* = 7.4 Hz, H_2_-3′), 1.62 (1H, dd, *J* = 10.0, 3.2 Hz, H-1), 1.99 (1H, m, H-9β), 2.00 (3H, s, H_3_-14b), 2.00 (1H, overlapped, H-8β), 2.03 (3H, s, H_3_-12b), 2.11 (3H, s, H_3_-2b), 2.27 (1H, dd, *J* = 9.7, 3.6 Hz, H-5), 2.28 (2H, td, *J* = 7.4, 2.2 Hz, H_2_-2′), 2.40 (2H, AB, H-11), 3.74 (1H, d, *J* = 11.4 Hz, H-15″), 4.05 (1H, d, *J* = 11.4 Hz, H-15′), 4.21 (2H, AB, H_2_-14), 4.42 (1H, d, *J* = 12.4 Hz, H-12″), 4.56 (1H, dd, *J* = 12.4, 1.4 Hz, H-12′), 6.46 (1H, s, H-3), 6.67 (1H, d, *J* = 10.2 Hz, H-2); ^13^C NMR (DMSO-*d*_6_, 151 MHz) δ 13.8 (CH_3_-6′), 20.6 (CH_3_-14b), 20.7 (CH_2_-8), 20.8 (CH_3_-2b, CH_3_-12b), 21.7 (CH_2_-5′), 24.1 (CH_2_-3′), 20.9 (CH_3_-2b), 24.3 (CH-7), 25.7 (C-13), 27.5 (CH-6), 29.7 (CH-5), 30.5 (CH_2_-4′), 33.4 (CH_2_-2′), 33.5 (CH_2_-9), 49.1 (CH-1), 51.2 (CH_2_-11), 59.2 (C-10), 60.9 (CH_2_-14), 62.3 (CH_2_-12), 68.6 (CH_2_-15), 91.1 (CH-2), 116.0 (C-4), 139.6 (CH-3), 169.2 (C-2a), 170.5 (C-12a), 170.6 (C-14a), 172.8 (C-1′); HR-ESI/MS, see **Supplementary Table S2**.

Pp_5: diplophyllin (Ohta et al., 1977): HR-ESI/MS, see **Supplementary Table S2**; ^1^H and ^13^C NMR data, see **Supplementary Material S1**.

Four compounds were obtained from *P. epiphylla* (the yields for each compound are the sum of the compound isolated from the two extracts) in DCM extract from 60 mg: Pe_1 (15.2 mg), Pe_2 (4.8 mg), Pe_3 (3.3 mg), and Pp_4 (2.3 mg).

Pe_1: 10-hydroxyperrottetin E (Cullmann et al., 1997), HR-ESI/MS, see **Supplementary Table S2**; ^1^H and ^13^C NMR data, see **Supplementary Material S1**.

Pe_2: perrottetin E (Cullmann et al., 1997), HR-ESI/MS, see **Supplementary Table S2**; ^1^H and ^13^C NMR data, see **Supplementary Material S1**.

Pe_3: 10-hydroxy-11-methoxy-perrottetin E, HR-ESI/MS, see **Supplementary Table S2**; ^1^H and ^13^C NMR data, see **Supplementary Material S1**.

Pe_4: 11-methoxy-perrottetin E, HR-ESI/MS, see **Supplementary Table S2**; ^1^H and ^13^C NMR data, see **Supplementary Material S1**.

Four compounds were obtained from *D. scoparium* (the yields for each compound are the sum of the compound isolated from the two extracts) in DCM extract from 40 mg: Ds_1 (0.5 mg), Ds_2 (0.7 mg), Ds_3 (0.5 mg), and Ds_4 (0.5 mg).

Ds_1: apigenin 7-*O*-[2,4-di-*O*-(α-l-rhamnopyranosyl)]-β-d-glucopyranoside (Becker et al., 1986), HR-ESI/MS, see **Supplementary Table S2**; ^1^H and ^13^C NMR data, see **Supplementary Material S1**.

Ds_2: 7-[(*O*-6-deoxy-α-l-mannopyranosyl-(1→2)-*O*-[6-deoxy-α-l-mannopyranosyl-(1→4)]-β-d-glucopyranosyl)oxy]-5-hydroxy-2-(3-hydroxy-4-methoxyphenyl)-4*H*-1-benzopyran-4-one (Osterdahl, 1978), HR-ESI/MS, see **Supplementary Table S2**; ^1^H and ^13^C NMR data, see **Supplementary Material S1**.

Ds_3: tiliroside = kaempferol-3-β-d-(6-*O*-*trans*-*p*-coumaroyl)glucopyranoside (Tsukamoto et al., 2004), HR-ESI/MS: see **Supplementary Table S2**, ^1^H and ^13^C NMR data, see **Supplementary Table S2**.

Ds_4: 7-hydroxy-2,4,10-trimethoxyphenanthrene ^1^H NMR (DMSO-*d*_6_, 600 MHz) δ 3.89 (3H, s, 2OCH_3_), 4.02 (3H, s, 10OCH_3_), 4.04 (3H, s, 4OCH_3_), 6.87 (1H, d, *J* = 2.6 Hz, H-3), 6.93 (1H, dd, *J* = 9.2, 2.7 Hz, H-6), 7.06 (1H, s, H-9), 7.12 (1H, d, *J* = 2.7 Hz, H-8), 7.29 (1H, d, *J* = 2.6 Hz, H-1), 9.17 (1H, d, *J* = 9.2 Hz, H-5), 9.54 (1H, s, 7OH); ^13^C NMR (DMSO-*d*_6_, 151 MHz) δ 55.1 (2OCH_3_), 55.5 (10OCH_3_), 55.8 (4OCH_3_), 95.0 (CH-1), 99.8 (CH-3), 103.4 (CH-9), 110.5 (CH-8), 114.3 (CH-6), 116.0 (C-12), 118.9 (C-13), 127.5 (C-11), 128.6 (CH-5), 133.7 (C-14), 152.2 (C-10), 154.9 (C-7), 157.1 (C-2), 158.7 (C-4); HR-ESI/MS, see **Supplementary Table S2**.

One compound was obtained from *A. curtipendula* (the yields for each compound are the sum of the compound isolated from the two extracts) in MeOH extract from 100 mg: Ac_01 (0.2 mg).

Ac_01: 6,7-dihydroxy-2-methoxyphenanthrene ^1^H NMR (DMSO-*d*_6_, 600 MHz) δ 3.88 (3H, s, 2OCH_3_), 7.18 (1H, s, H-8), 7.20 (1H, dd, *J* = 9.0, 2.8 Hz, H-3), 7.33 (1H, d, *J* = 2.8 Hz, H-1), 7.49 (1H, d, *J* = 8.8 Hz, H-10), 7.55 (1H, d, *J* = 8.8 Hz, H-9), 7.89 (1H, s, H-5), 8.33 (1H, d, *J* = 9.0 Hz, H-4); ^1^H NMR (DMSO-*d*_6_, 151 MHz) δ 55.1 (2OCH_3_), 106.6 (CH-5), 108.3 (CH-1), 112.1 (CH-8), 116.5 (CH-3), 123.3 (CH-10), 123.7 (CH-4), 124.3 (C-13), 125.2 (C-14), 126.5 (CH-9), 131.9 (C-11), 145.9 (C-7), 147.0 (C-6), 156.9 (C-2); HR-ESI/MS, see **Supplementary Table S2**.

### 4.4 Statistical and diversity analyses

Statistical analyses were carried out using R 4.0.2 and the following additional packages: dplyr, ComplexHeatmap, and circlize. The statistical analyses, peak, and classification tables were normalized by species. Missing values were imputed with zeros. The data obtained are summarized in the PI and NI heatmaps (**Figure 2**). The color gradient from red to yellow in PI and dark blue to light blue in NI is expressed horizontally and corresponds to the abundance of compound classes by species. Species are divided into two phyla (liverworts and mosses) and ordered according to their taxonomic proximity. Species marked with an asterisk have been selected and isolated to a validated cluster in MN. The barplot shows the number of compounds annotated (in light gray) versus the number of compounds not annotated (in dark gray).

## Data Availability

The datasets presented in this study can be found in online repositories. The names of the repository/repositories and accession number(s) can be found in the article/**Supplementary Material**.

## References

[B1] Acebey-Castellon I. L. Voutquenne-Nazabadioko L. Doan Thi Mai H. Roseau N. Bouthagane N. Muhammad D.. (2011). Triterpenoid saponins from *symplocos lancifolia* . J. Nat. Prod. 74, 163–168. doi: 10.1021/np100502y 21288041

[B2] Allard P.-M. Gaudry A. Quirós-Guerrero L.-M. Rutz A. Dounoue-Kubo M. Walker T. W. N.. (2023). Open and reusable annotated mass spectrometry dataset of a chemodiverse collection of 1,600 plant extracts. GigaScience 12, giac124. doi: 10.1093/gigascience/giac124 PMC9845059 36649739

[B3] Allard P.-M. Péresse T. Bisson J. Gindro K. Marcourt L. Pham V. C.. (2016). Integration of molecular networking and in-Silico MS/MS Fragmentation for natural products dereplication. Anal. Chem. 88, 3317–3323. doi: 10.1021/acs.analchem.5b04804 26882108

[B4] Asakawa Y. (1982). “Chemical constituents of the hepaticae,” in Fortschritte der Chemie organischer Naturstoffe / Progress in the Chemistry of Organic Natural Products (Springer, Vienna), 1–285. doi: 10.1007/978-3-7091-8677-0_1

[B5] Asakawa Y. (1995). “Chemical constituents of the bryophytes,” in Progress in the Chemistry of Organic Natural Products (Springer, Vienna), 1–562. doi: 10.1007/978-3-7091-6896-7_1 23556317

[B6] Asakawa Y. (2012). Bio- and chemical diversity of bryophytes: chemical structures and bioactivity of scents, and related compounds. Aroma Res. 13, 270–278.

[B7] Asakawa Y. Ludwiczuk A. (2013). “Bryophytes: Liverworts, Mosses, and Hornworts: extraction and isolation procedures,” in Metabolomics Tools for Natural Product Discovery (Humana Press, Totowa, NJ), 1–20. doi: 10.1007/978-1-62703-577-4_1 23963899

[B8] Asakawa Y. Ludwiczuk A. Nagashima F. (2013a). Phytochemical and biological studies of bryophytes. Phytochemistry 91, 52–80. doi: 10.1016/j.phytochem.2012.04.012 22652242

[B9] Asakawa Y. Ludwiczuk D. A. Nagashima D. F. (2013b). “Chemical diversity of bryophytes,” in Chemical Constituents of Bryophytes (Springer, Vienna), 21–24. doi: 10.1007/978-3-7091-1084-3_3 23556317

[B10] Asakawa Y. Ludwiczuk A. Novakovic M. Bukvicki D. Anchang K. Y. (2021). Bis-bibenzyls, bibenzyls, and terpenoids in 33 genera of the marchantiophyta (Liverworts): structures, synthesis, and bioactivity. J. Nat. Prod. 85 (3), 729–762. doi: 10.1021/acs.jnatprod.1c00302 34783552

[B11] Asakawa Y. Matsuda R. Toyota M. Hattori S. Ourisson G. (1981). Terpenoids and bibenzyls of 25 liverwort *Frullania* species. Phytochemistry 20, 2187–2194. doi: 10.1016/0031-9422(81)80111-2

[B12] Asakawa Y. Toyota M. Takemoto T. Suire C. (1979). lagiochilines C, D, E and F, four novel secoaromadendrane-type sesquiterpene hemiacetals from *Plagiochila asplenioides* and *Plagiochila semidecurrens* . Phytochemistry 18, 1355–1357. doi: 10.1016/0031-9422(79)83021-6

[B13] Augier J. (1966). Flore des bryophytes (Paris: P. Lechevalier).

[B14] Becker R. Mues R. Zinsmeister H. D. Herzog F. Geiger H. (1986). A new biflavone and further flavonoids from the moss *Hylocomium splendens* . Z. für Naturforschung C 41, 507–510. doi: 10.1515/znc-1986-5-602

[B15] Chou Y. Liao C. (2013). First asymmetric total syntheses and determination of absolute configurations of (+)-Eudesmadiene-12,6-olide and (+)-Frullanolide. Organic letter 15, 1584–1587. doi: 10.1021/ol4003724 23484913

[B16] Christenhusz M. J. M. Byng J. W. (2016). The number of known plants species in the world and its annual increase. Phytotaxa 261, 201–217. doi: 10.11646/phytotaxa.261.3.1

[B17] Cullmann F. Becker H. Pandolfi E. Roeckner E. Eicher T. (1997). Bibenzyl derivatives from *Pellia epiphylla* . Phytochemistry 45, 1235–1247. doi: 10.1016/S0031-9422(97)00118-0

[B18] Dührkop K. Fleischauer M. Ludwig M. Aksenov A. A. Melnik A. V. Meusel M.. (2019). SIRIUS 4: a rapid tool for turning tandem mass spectra into metabolite structure information. Nat. Methods 16, 299–302. doi: 10.1038/s41592-019-0344-8 30886413

[B19] Dührkop K. Nothias L.-F. Fleischauer M. Reher R. Ludwig M. Hoffmann M. A.. (2021). Systematic classification of unknown metabolites using high-resolution fragmentation mass spectra. Nat. Biotechnol. 39, 462–471. doi: 10.1038/s41587-020-0740-8 33230292

[B20] Dührkop K. Shen H. Meusel M. Rousu J. Böcker S. (2015). Searching molecular structure databases with tandem mass spectra using CSI : FingerID. Proc. Natl. Acad. Sci. 112, 12580–12585. doi: 10.1073/pnas.1509788112 26392543 PMC4611636

[B21] Fan G.-X. Dong L.-L. Li H.-H. Li Z.-Y. Zhang Z.-X. Fei D.-Q. (2016). Sesquiterpenoids and other chemical components from the roots of *dolomiaea souliei* . Chem. Nat. Compd 52, 754–757. doi: 10.1007/s10600-016-1766-5

[B22] Guillarme D. Nguyen D. T. T. Rudaz S. Veuthey J.-L. (2008). Method transfer for fast liquid chromatography in pharmaceutical analysis: Application to short columns packed with small particle. Part II: Gradient experiments. Eur. J. Pharmaceutics Biopharmaceutics 68, 430–440. doi: 10.1016/j.ejpb.2007.06.018 17703929

[B23] Hoffmann M. A. Nothias L.-F. Ludwig M. Fleischauer M. Gentry E. C. Witting M.. (2022). High-confidence structural annotation of metabolites absent from spectral libraries. Nat. Biotechnol. 40, 411–421. doi: 10.1038/s41587-021-01045-9 34650271 PMC8926923

[B24] Horn A. Pascal A. Lončarević I. Volpatto Marques R. Lu Y. Miguel S.. (2021). Natural products from bryophytes: from basic biology to biotechnological applications. Crit. Rev. Plant Sci. 40, 191–217. doi: 10.1080/07352689.2021.1911034

[B25] Kraut L. Mues R. Sim-Sim M. (1994). Sesquiterpene lactones and 3-benzylphthalides from *Frullania muscicola* . Phytochemistry 37, 1337–1346. doi: 10.1016/S0031-9422(00)90409-6

[B26] Lu Y. Eiriksson F. F. Thorsteinsdottir M. Simonsen H. T. (2019). Valuable fatty acids in bryophytes—production, biosynthesis, analysis and applications. Plants 8, 524. doi: 10.3390/plants8110524 31752421 PMC6918284

[B27] Ludwig M. Nothias L.-F. Dührkop K. Koester I. Fleischauer M. Hoffmann M. A.. (2020). Database-independent molecular formula annotation using Gibbs sampling through ZODIAC. Nat. Mach. Intell. 2, 629–641. doi: 10.1038/s42256-020-00234-6

[B28] Martini U. Zapp J. Becker H. (1998). Chlorinated macrocyclic bisbibenzyls from the liverwort *Bazzania trilobata* . Phytochemistry 47, 89–96. doi: 10.1016/S0031-9422(97)00495-0

[B29] Nadgouda S. A. Trivedi G. K. Bhattacharyya S. C. (1978). Sensitized photo oxygenation of alpha cyclo costunolide and di hydro alpha cyclo costunolide a biogenetic type transformation of costunolide to santonin. Indian J. Chem. Sect. B 16, 16–19.

[B30] Nagashima F. Tanaka H. Toyota M. Hashimoto T. Kan Y. Takaoka S.. (1994). Sesqui- and diterpenoids from *Plagiochila* species. Phytochemistry 36, 1425–1430. doi: 10.1016/S0031-9422(00)89735-6

[B31] Nothias L.-F. Petras D. Schmid R. Dührkop K. Rainer J. Sarvepalli A.. (2020). Feature-based molecular networking in the GNPS analysis environment. Nat. Methods 17, 905–908. doi: 10.1038/s41592-020-0933-6 32839597 PMC7885687

[B32] Novaković M. Ludwiczuk A. Bukvički D. Asakawa Y. (2021). Phytochemicals from bryophytes: Structures and biological activity. J. Serbian Chem. Soc. 86, 1139–1175. doi: 10.2298/JSC211027100N

[B33] Ohta Y. Andersen N. H. Liu C.-B. (1977). Sesquiterpene constituents of two liverworts of genus *diplophyllum*: Novel eudesmanolides and cytotoxicity studies for enantiomeric methylene lactones. Tetrahedron 33, 617–628. doi: 10.1016/0040-4020(77)80301-3

[B34] Osterdahl B. (1978). Chemical studies on bryophytes.20. New branched flavonoid-omicron-triglycoside from *dicranum-scoparium* . Acta Chemica Scandinavica Ser. B-Organic Chem. Biochem. 32, 714–716. doi: 10.3891/acta.chem.scand.32b-0714

[B35] Pannequin A. (2019). Caractérisation chimique des bryophytes de Corse et propriétés biologiques (Université Pascal Paoli). Available online at: https://tel.archives-ouvertes.fr/tel-03482569 (Accessed March 10, 2022).

[B36] Pannequin A. Laurini E. Giordano L. Muselli A. Pricl S. Tintaru A. (2020). Caution: chemical instability of natural biomolecules during routine analysis. Molecules 25, 3292. doi: 10.3390/molecules25143292 32698478 PMC7397321

[B37] Pannequin A. Quetin-Leclercq J. Costa J. Tintaru A. Muselli A. (2023). First phytochemical profiling and *in-vitro* antiprotozoal activity of essential oil and extract of *plagiochila porelloides* . Molecules 28, 616. doi: 10.3390/molecules28020616 36677674 PMC9860869

[B38] Pannequin A. Tintaru A. Desjobert J.-M. Costa J. Muselli A. (2017). New advances in the volatile metabolites of *Frullania tamarisci* . Flavour Fragr J. 32:409–418. doi: 10.1002/ffj.3407

[B39] Peters K. Balcke G. Kleinenkuhnen N. Treutler H. Neumann S. (2021). Untargeted in silico compound classification—A novel metabolomics method to assess the chemodiversity in bryophytes. Int. J. Mol. Sci. 22, 3251. doi: 10.3390/ijms22063251 33806786 PMC8005083

[B40] Peters K. Treutler H. Döll S. Kindt A. S. D. Hankemeier T. Neumann S. (2019). Chemical diversity and classification of secondary metabolites in nine bryophyte species. Metabolites 9, 222. doi: 10.3390/metabo9100222 31614655 PMC6835487

[B41] Queiroz E. F. Alfattani A. Afzan A. Marcourt L. Guillarme D. Wolfender J.-L. (2019). Utility of dry load injection for an efficient natural products isolation at the semi-preparative chromatographic scale. J. Chromatogr. A 1598, 85–91. doi: 10.1016/j.chroma.2019.03.042 30926257

[B42] Ramírez M. Kamiya N. Popich S. Asakawa Y. Bardón A. (2017). Constituents of the Argentine Liverwort *Plagiochila diversifolia* and their insecticidal activities. Chem. Biodiversity 14, e1700229. doi: 10.1002/cbdv.201700229 29024401

[B43] Rutz A. Dounoue-Kubo M. Ollivier S. Bisson J. Bagheri M. Saesong T.. (2019). Taxonomically informed scoring enhances confidence in natural products annotation. Front. Plant Sci. 10. doi: 10.3389/fpls.2019.01329 PMC6824209 31708947

[B44] Rutz A. Sorokina M. Galgonek J. Mietchen D. Willighagen E. Gaudry A.. (2022). The LOTUS initiative for open knowledge management in natural products research. eLife 11, e70780. doi: 10.7554/eLife.70780 35616633 PMC9135406

[B45] Sangsopha W. Lekphrom R. Kanokmedhakul S. Kanokmedhakul K. (2016). Cytotoxic and antimalarial constituents from aerial parts of *Sphaeranthus indicus* . Phytochem. Lett. 17, 278–281. doi: 10.1016/j.phytol.2016.08.001

[B46] Sartori S. K. Diaz M. A. N. Diaz-Muñoz G. (2021). Lactones: Classification, synthesis, biological activities, and industrial applications. Tetrahedron 84, 132001. doi: 10.1016/j.tet.2021.132001

[B47] Scher J. M. Zapp J. Schmidt A. Becker H. (2003). Bazzanins L–R, chlorinated macrocyclic bisbibenzyls from the liverwort *Lepidozia incurvata* . Phytochemistry 64, 791–796. doi: 10.1016/S0031-9422(03)00382-0 13679103

[B48] Schmid R. Heuckeroth S. Korf A. Smirnov A. Myers O. Dyrlund T. S.. (2023). Integrative analysis of multimodal mass spectrometry data in MZmine 3. Nat. Biotechnol. 41, 447–449. doi: 10.1038/s41587-023-01690-2 36859716 PMC10496610

[B49] Shen S. Zhan C. Yang C. Fernie A. R. Luo J. (2023). Metabolomics-centered mining of plant metabolic diversity and function: Past decade and future perspectives. Mol. Plant 16, 43–63. doi: 10.1016/j.molp.2022.09.007 36114669

[B50] Smoot M. E. Ono K. Ruscheinski J. Wang P.-L. Ideker T. (2011). Cytoscape 2.8: new features for data integration and network visualization. Bioinformatics 27, 431–432. doi: 10.1093/bioinformatics/btq675 21149340 PMC3031041

[B51] Sotiaux A. Pioli A. Royaud A. Schumacker R. Vanderpoorten A. (2007). A checklist of the bryophytes of Corsica (France): new records and a review of the literature. J. Bryology 29, 41–53. doi: 10.1179/174328207X171872

[B52] Sotiaux A. Sotiaux O. Vanderpoorten A. (2008). Additions to the bryophyte flora of Corsica. Cryoptogamie Bryologie 29, 267–274.

[B53] Spörle J. Becker H. Allen N. S. Gupta M. P. (1991). Spiroterpenoids from plagiochila moritziana. Phytochemistry 30, 3043–3047. doi: 10.1016/S0031-9422(00)98249-9

[B54] Toyota M. Nakamura I. Huneck S. Asakawa Y. (1994). Sesquiterpene esters from the liverwort *Plagiochila porelloides* . Phytochemistry 37, 1091–1093. doi: 10.1016/S0031-9422(00)89535-7

[B55] Toyota M. Nishimoto C. Asakawa Y. (1998). Eudesmane-type sesquiterpenoids from Japanese liverwort. Frullania tamarisci subsp. obscura. 46, 542–544.

[B56] Tsukamoto S. Tomise K. Aburatani M. Onuki H. Hirorta H. Ishiharajima E.. (2004). Isolation of cytochrome P450 inhibitors from strawberry fruit, *Fragaria ananassa* . J. Nat. Prod 67, 1839–1841. doi: 10.1021/np0400104 15568772

[B57] Vergoten G. Bailly C. (2023). The Plagiochilins from *Plagiochila* Liverworts: binding to α-Tubulin and drug design perspectives. AppliedChem 3, 217–228. doi: 10.3390/appliedchem3020014

[B58] von Reuß S. H. König W. A. (2004). Corsifurans A–C, 2-arylbenzofurans of presumed stilbenoid origin from *Corsinia coriandrina* (Hepaticae). Phytochemistry 65, 3113–3118. doi: 10.1016/j.phytochem.2004.10.002 15541739

[B59] von Reuß S. H. König W. A. (2005). Olefinic isothiocyanates and iminodithiocarbonates from the liverwort corsinia coriandrina. Eur. J. Organic Chem. 2005, 1184–1188. doi: 10.1002/ejoc.200400586

[B60] Wolfender J.-L. Nuzillard J.-M. van der Hooft J. J. J. Renault J.-H. Bertrand S. (2019). Accelerating metabolite identification in natural product research: toward an ideal combination of liquid chromatography–high-resolution tandem mass spectrometry and NMR profiling, in silico databases, and chemometrics. Anal. Chem. 91, 704–742. doi: 10.1021/acs.analchem.8b05112 30453740

[B61] Zhang Z. Li Y. Sun Y. Wang W. Song X. Zhang D. (2023). Chemical diversity and biological activities of marine-derived sulphur containing alkaloids: A comprehensive update. Arabian J. Chem. 16, 105011. doi: 10.1016/j.arabjc.2023.105011

