# Supplementary material for: Comprehensive comparative metabolome study of a large collection of Corsican bryophytes
Source: Front Plant Sci. 2025 Jan 7;15:1470307. doi: 10.3389/fpls.2024.1470307 (PMC11745878; doi:10.3389/fpls.2024.1470307)
Supplement: Supplementary file 3 [file DataSheet1.pdf]

Supplementary

Figure S1: Taxonomic organization of bryophyte species in France obtained from TAXAREF data. The dots correspond to the liverworts (green) and mosses (blue) present in our collection. 3

Figure S2: Box plots of (A) extraction yields and (B) sample clean-up yields. Dotted points show all samples, solid points show outliers: a) *Marchantia paleacea* c) *Grimmia ramondii* b) *Grimmia pulvinata* d) *Frullania tamarisci* e) *Porella obtusata* f) *Porella arboris-vitae* g) *Pterigynandrum filiforme* h) *Bartramia pomiformis* i) *Orthotrichum rupestre* j) *Tortula muralis* k) *Pterigynandrum filiforme*. Red crosses correspond to the mean value. (C) The minimum (Min), 1st quartile (1st Qu), median, mean, 3rd quartile (3rd Qu), maximum (Max) and standard deviation (St)of extract and sample clean-up yields used to analyze the efficient process........ 4

Figure S3: A) Sample preparation workflow applied to the bryophyte extract collection. B) Table of extract yields after sample preparation on 5 selected species. C) ELSD profile of *Dicranum scoparium* and *Plagiochila porelloides* MeOH extracts after sample clean-up and sample enrichment........ 5

Figure S4: Nodes of species colour coding........ 6

Figure S5: Fractionation details 7

Figure S6: Structures of isolated compounds (Ft_ : *Frullania tamarisci*, Pp_ : *Plagiochila porelloides*, Ac_ : *Antitrichia curtipendula*, Pe_ : *Pellia epiphylla* and Ds_ : *Dicranum scoparium*) and standard compounds (St_) added in our MN. * Corresponds to stilbenoids in NPClassifier. 8

Figure S7: ^{1}H NMR spectrum of compound **Ft_1** in $CDCl_3$ at 600 MHz 9

Figure S8: COSY NMR spectrum of compound **Ft_1** in $CDCl_3$........ 9

Figure S9: ^{13}C-DEPTQ NMR spectrum of compound **Ft_1** in $CDCl_3$ at 151 MHz 10

Figure S10: Edited HSQC NMR spectrum of compound **Ft_1** in $CDCl_3$ 10

Figure S11: HMBC NMR spectrum of compound **Ft_1** in $CDCl_3$........ 11

Figure S12: ROESY NMR spectrum of compound **Ft_1** in $CDCl_3$ 11

Figure S13: ^{1}H NMR spectrum of compound **Ft_5** in $CDCl_3$ at 600 MHz 12

Figure S14: COSY NMR spectrum of compound **Ft_5** in $CDCl_3$........ 12

Figure S15: ^{13}C-DEPTQ NMR spectrum of compound **Ft_5** in $CDCl_3$ at 151 MHz........ 13

Figure S16: Edited HSQC NMR spectrum of compound **Ft_5** in $CDCl_3$ 13

Figure S17: HMBC NMR spectrum of compound **Ft_5** in $CDCl_3$........ 14

Figure S18: NOESY NMR spectrum of compound **Ft_5** in $CDCl_3$ 14

Figure S19: ^{1}H NMR spectrum of compound **Pp_4** in DMSO-d_6 at 600 MHz 15
Figure S20: COSY NMR spectrum of compound **Pp_4** in DMSO-d_6 15
Figure S21: Edited HSQC NMR spectrum of compound **Pp_4** in DMSO-d_6 16
Figure S22: HMBC NMR spectrum of compound **Pp_4** in DMSO-d_6 16
Figure S23: ROESY NMR spectrum of compound **Pp_4** in DMSO-d_6 17
Figure S24: ^{1}H NMR spectrum of compound **Ds_4** in DMSO-d_6 at 600 MHz 17
Figure S25: COSY NMR spectrum of compound **Ds_4** in DMSO-d_6 18
Figure S26: Edited HSQC NMR spectrum of compound **Ds_4** in DMSO-d_6 18
Figure S27: HMBC NMR spectrum of compound **Ds_4** in DMSO-d_6 19
Figure S28: ROESY NMR spectrum of compound **Ds_4** in DMSO-d_6 19
Figure S29: ^{1}H NMR spectrum of compound **Ac_01** in DMSO-d_6 at 600 MHz 20
Figure S30: COSY NMR spectrum of compound **Ac_01** in DMSO-d_6 20
Figure S31: Edited HSQC NMR spectrum of compound **Ac_01** in DMSO-d_6 21
Figure S32: HMBC NMR spectrum of compound **Ac_01** in DMSO-d_6 21
Figure S33: ROESY NMR spectrum of compound **Ac_01** in DMSO-d_6 22
Supplementary data 1: NMR data of isolated compounds previously reported in the literature

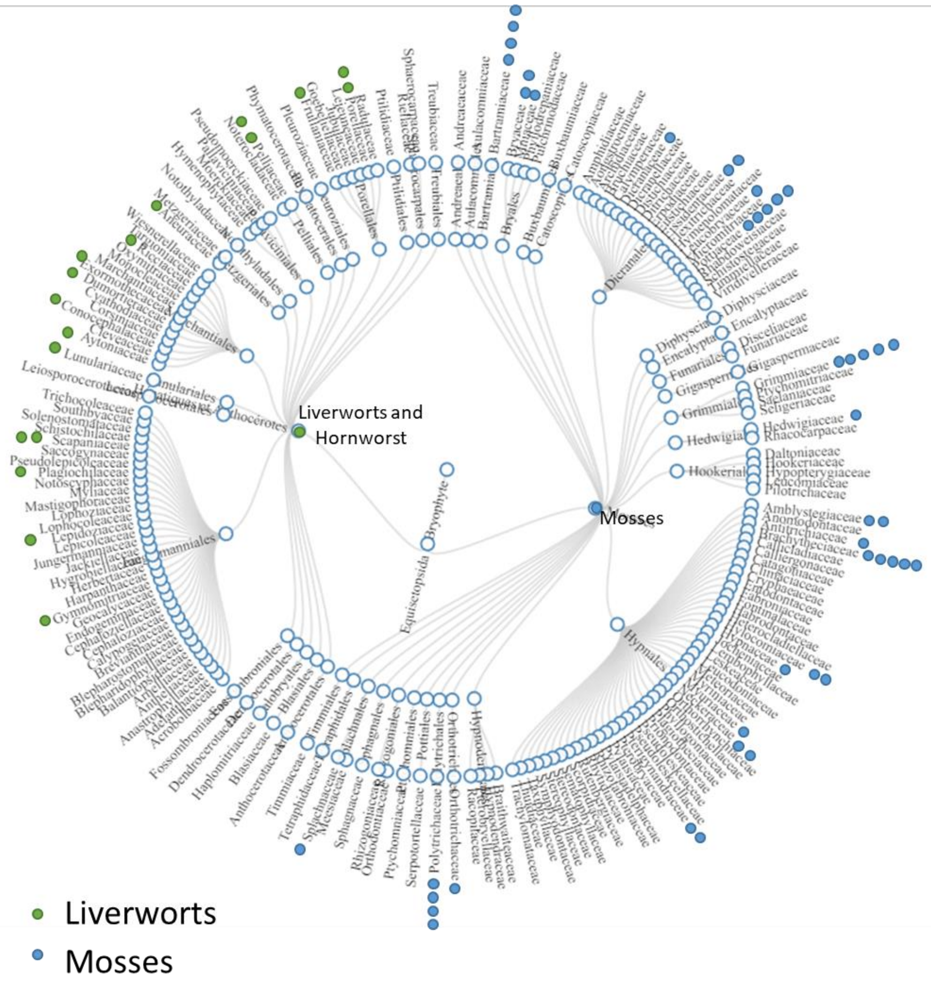

Figure S1: Taxonomic organization of bryophyte species in France obtained from TAXAREF data. The dots correspond to the liverworts (green) and mosses (blue) present in our collection.

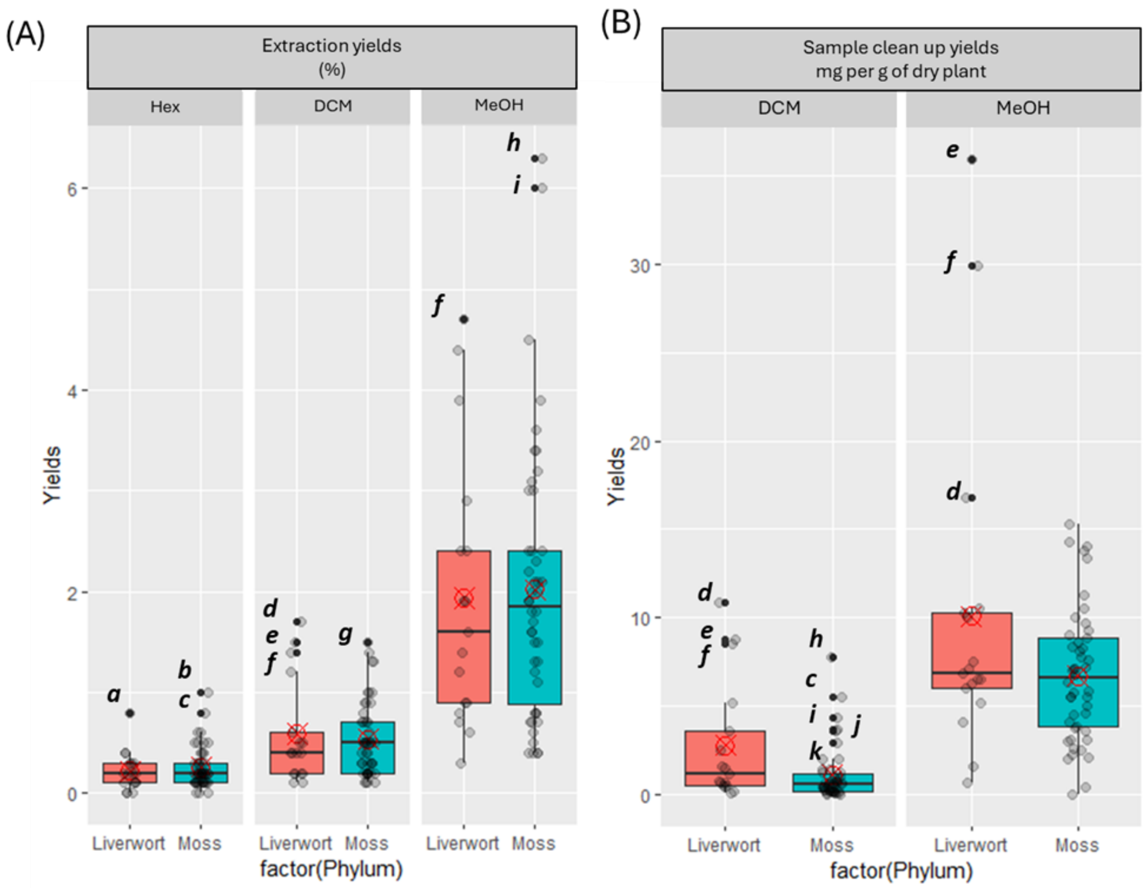

(C)

	Extraction yields (%)						sample clean-up yields (mg per g of dry plant)			
	Hex		DCM		MeOH		DCM		MeOH	
	Liverwort	Moss	Liverwort	Moss	Liverwort	Moss	Liverwort	Moss	Liverwort	Moss
Min	0,00	0,00	0,10	0,10	0,30	0,40	0,10	0,00	0,70	0,00
1st Qu	0,10	0,10	0,20	0,20	0,90	0,88	0,50	0,60	6,00	3,83
Median	0,20	0,20	0,40	0,50	1,60	1,85	1,20	0,60	6,90	6,60
Mean	0,21	0,25	0,58	0,53	1,93	2,01	2,81	1,12	10,11	6,71
3rd Qu	0,30	0,30	0,60	0,70	2,40	2,40	3,60	1,23	10,30	8,83
Max	0,80	1,00	1,70	1,50	4,70	6,30	10,90	7,80	35,90	15,30
sd	0,19	0,22	0,52	0,37	1,35	1,36	3,44	1,57	9,37	3,77

Figure S2: Box plots of (A) extraction yields and (B) sample clean-up yields. Dotted points show all samples, solid points show outliers: a) *Marchantia paleacea* c) *Grimmia ramondii* b) *Grimmia pulvinata* d) *Frullania tamarisci* e) *Porella obtusata* f) *Porella arboris-vitae* g) *Pterigynandrum filiforme* h) *Bartramia pomiformis* i) *Orthotrichum rupestre* j) *Tortula muralis* k) *Pterigynandrum filiforme*. Red crosses correspond to the mean value. (C) The minimum (Min), 1st quartile (1st Qu), median, mean, 3rd quartile (3rd Qu), maximum (Max) and standard deviation (St)of extract and sample clean-up yields used to analyze the efficient process.

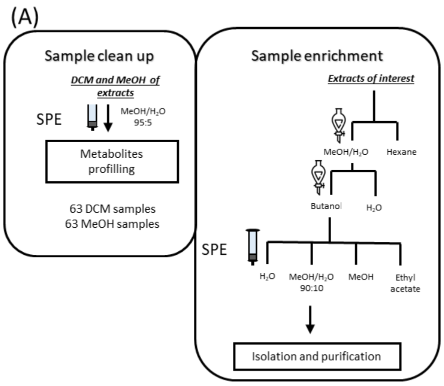

(B) Table : extract yields after sample preparation

	Sample clean up	Sample enrichment
Antitrichia curtipendula	68.0%	1.0%
Dicranium scoparium	35.4%	5.0%
Frullania tamarisci	58.3%	50.0 %
Pellia epiphylla	70.2 %	80.0%
Plagiochila porelloides	42.0%	53.0%

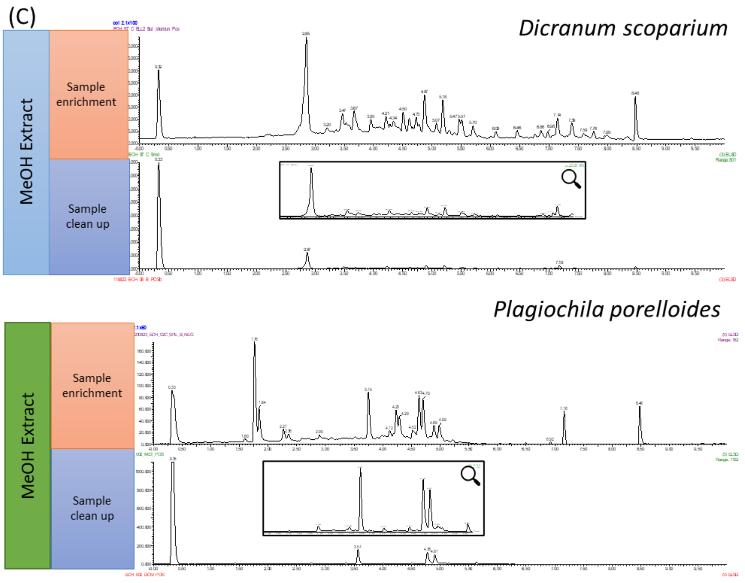

Figure S3: A) Sample preparation workflow applied to the bryophyte extract collection. B) Table of extract yields after sample preparation on 5 selected species. C) ELSD profile of *Dicranum scoparium* and *Plagiochila porelloides* MeOH extracts after sample clean-up and sample enrichment.

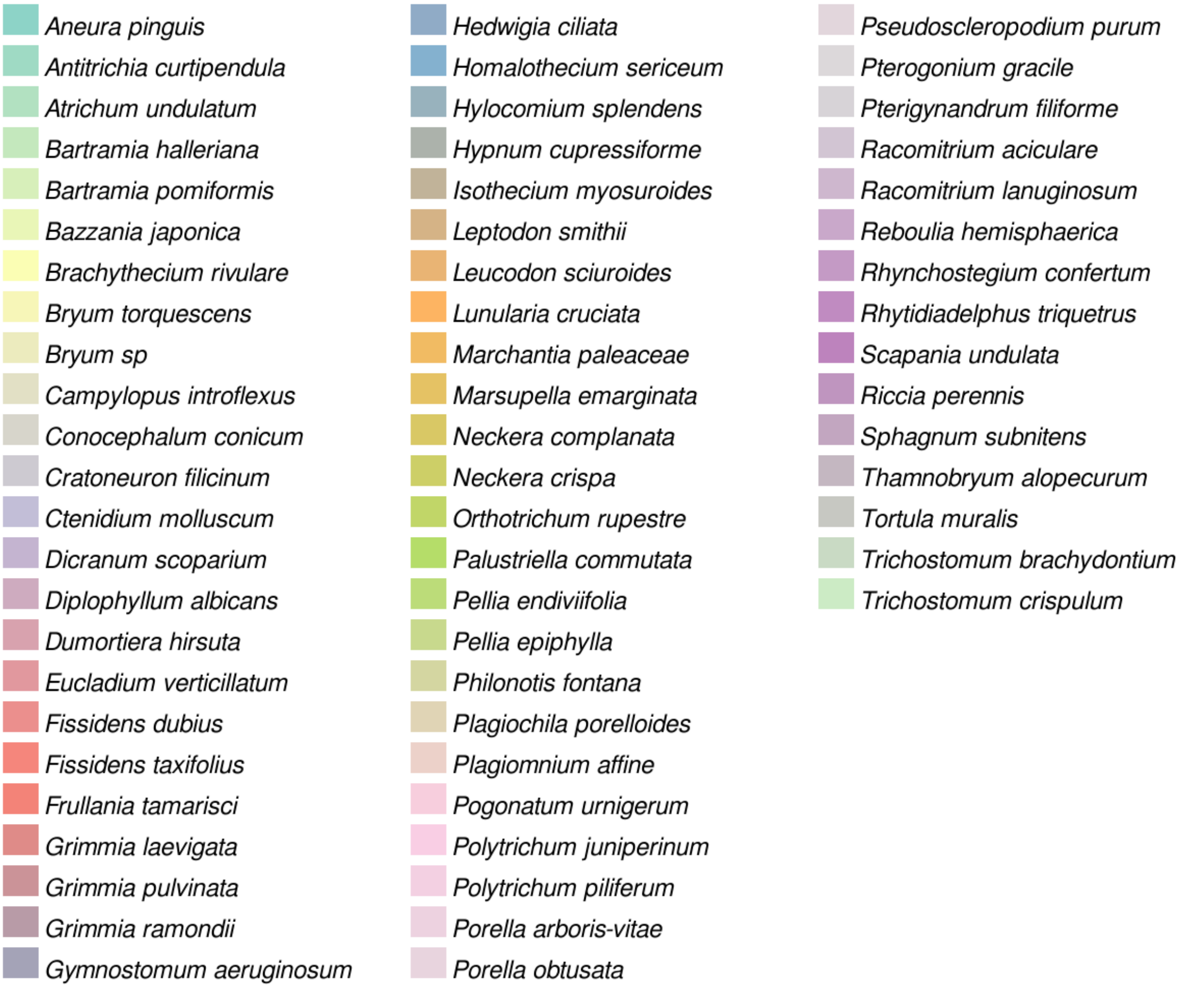

Figure S4: Nodes of species colour coding

		1	2	3	4	5	6	7	8	9	10	11	12	13	14	15	16	17	18
Frullania tamarisci DCM 50mg	Sub-fraction n°	1-8	9-11	12-14	15-19	20-24	25-28	29-33	34-43	44-48	49	50	51-53	54-56	57-60	61-68	69-73	14-77	78-96
	Mass	2,0	1,7	1,1	2,5	4,1	1,6	2,1	2,8	1,9	6,1	3,7	1,6	1,4	6,2	3,9	2,0	1,5	3,6
	Compound identity								**Ft_2**		**Ft_3**				**Ft_4**	**Ft_5**			

		1	2	3	4	5	6	7	8	9	10	11	12	13
Frullania tamarisci DCM 20mg	Sub-fraction n°	1-10	11-18	19-20	21-24	25-28	29-35	36-37	38-47	48-54	55-65	66-70	71-79	80-96
	Mass	3,0	1,0	0,6	0,9	1,0	2,5	1,2	1,1	1,7	1,5	1,8	2,0	1,0
	Compound identity					**Ft_1**							**Ft_6**	

		1	2	3	4	5	6	7	8	9	10	11	12	13	14	15	16	17	18	19	20	21	22	23	24	25	26	27	28	29	30
Plagiochila porelloides MeOH 100mg	Sub-fraction n°	1-3	4	5-6	7-8	9-10	11	12-13	14-15	16-17	18-19	20-21	22-24	25-26	27	28	29	30-31	32	33	34	35-36	37-38	39-40	41-43	44-45	46-48	49	50-51	52	53-54
	Mass	6,8	0,9	1,2	1,0	1,2	3,5	1,4	1,1	3,3	4,0	3,8	1,4	1,3	1,0	1,1	1,7	2,5	1,0	1,0	1,0	1,3	1,5	3,4	1,4	1,0	1,6	1,6	1,2	3,6	2,0
	Compound identity										**Pp_1**													**Pp_2**						**Pp_3**	

		31	32	33	34	35	36	37	38	39	40	41	42	43	44	45	46	47	48	49	50	51	52
Plagiochila porelloides MeOH 100mg (continued)	Sub-fraction n°	55-56	57-58	59-60	61-62	62-65	66	67-68	69-71	72-73	74	75-76	77-78	79-80	81	82	83	84	85-86	87-88	89-90	91-92	93-96
	Mass	1,8	3,6	1,7	0,9	1,3	0,2	0,8	0,9	0,9	0,9	1,0	1,1	0,7	0,6	0,5	2,7	0,8	1,6	3,0	0,9	3,4	0,8
	Compound identity		**Pp_4**																				

		1	2	3	4	5	6	7	8	9	10	11	12	13	14	15	16
Pellia epiphylla DCM 60mg	Sub-fraction n°	25-26	34	35	36-37	38	40-41	42-43	47-48	49-50	51-52	68	69	70-72	73-81	82-83	84-96
	Mass	2,0	3,0	2,7	15,2	1,9	4,8	2,7	3,3	2,1	2,3	1,9	1,7	2,1	3,7	3,1	5,1
	Compound identity				**Pe_1**		**Pe_2**		**Pe_3**		**Pe_4**						

		1	2	3	4	5	6	7	8	9	10	11
Dicranum scoparium MeOH 40mg	fractions	3	24	33	36	60	64	65	67	71-72	73	75
	masses	2,5	0,5	0,7	0,5	3,0	0,5	0,6	0,6	0,4	0,1	0,9
	compounds		**Ds_1**	**Ds_2**	**Ds_3**		**Ds_4**					

		1	2	3	4	5	6	7	8	9	10
Antrichia curtipendula MeOH 100mg	fractions	1-3	4-6	7-21	22	23-28	29-30	31-34	35-41	42-48	69-72
	masses	35,6	1,7	1,8	0,2	0,9	0,0	0,8	0,6	1,3	2,6
	compounds				**Ac_1**						

Figure S5: Fractionation details

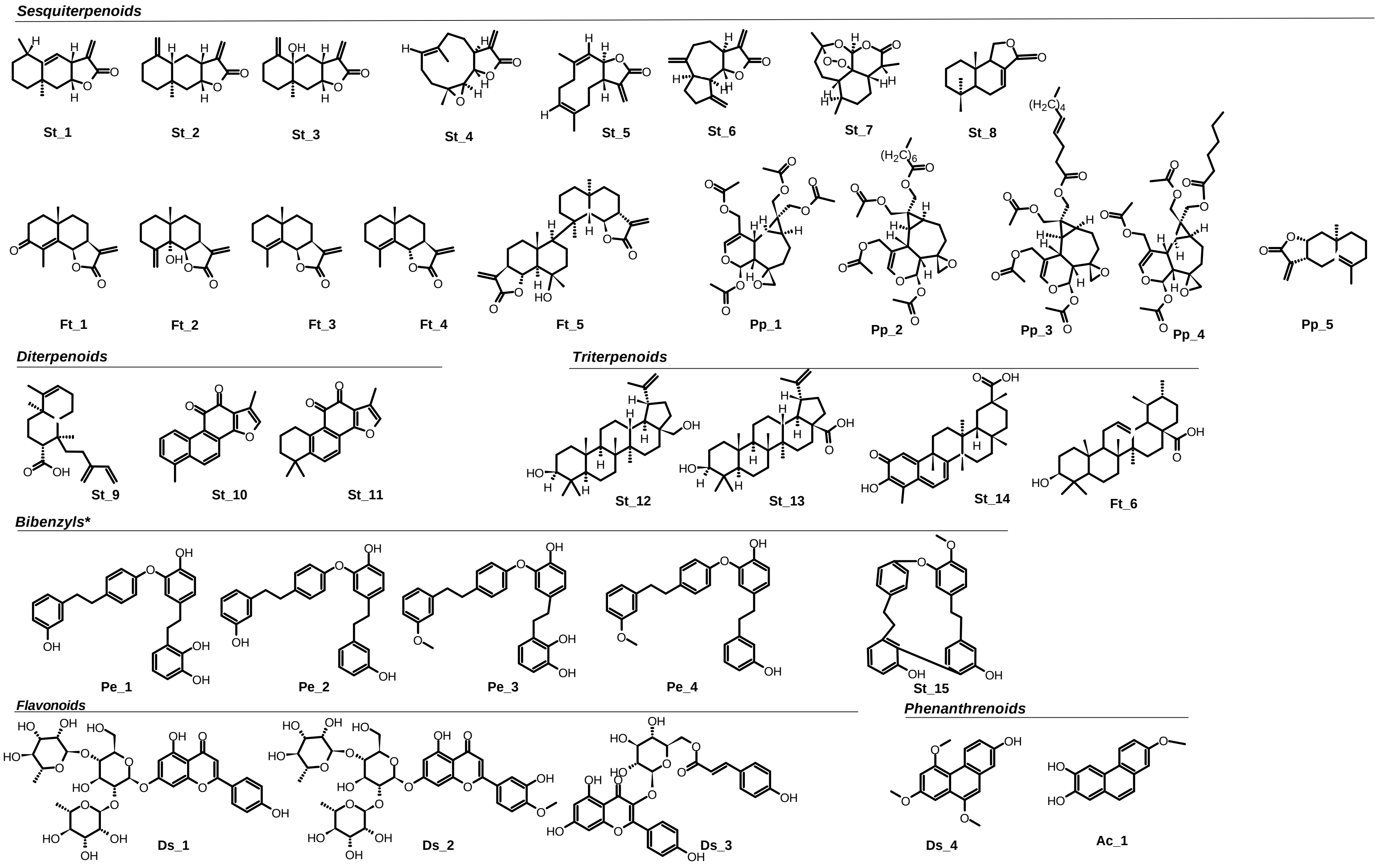

Figure S6: Structures of isolated compounds (Ft_ : *Frullania tamarisci*, Pp_ : *Plagiochila porelloides*, Ac_ : *Antitrichia curtipendula*, Pe_ : *Pellia epiphylla* and Ds_ : *Dicranum scoparium*) and standard compounds (St_) added in our MN. * Corresponds to stilbenoids in NPClassifier.

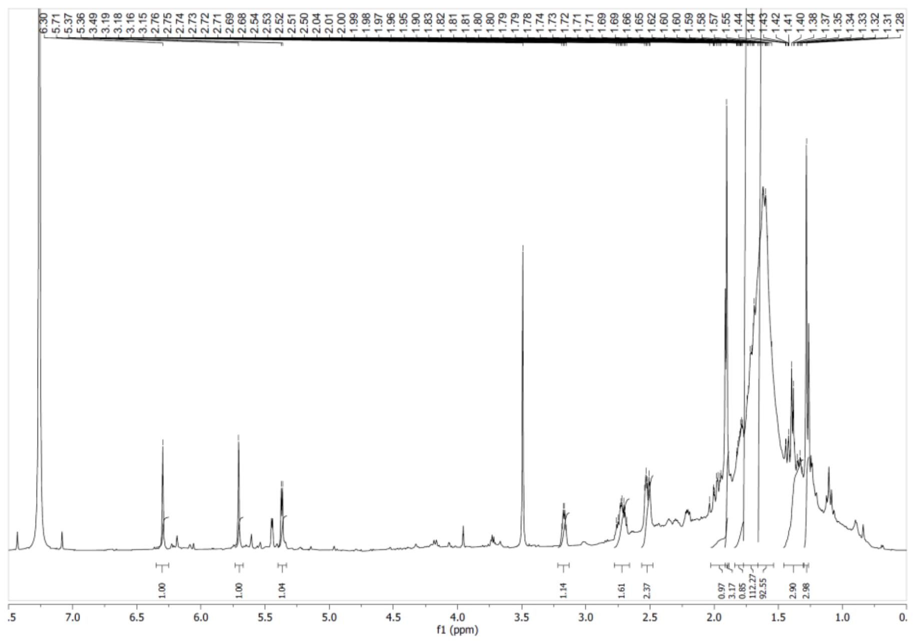

Figure S7: ^{1}H NMR spectrum of compound **Ft_1** in $CDCl_3$ at 600 MHz

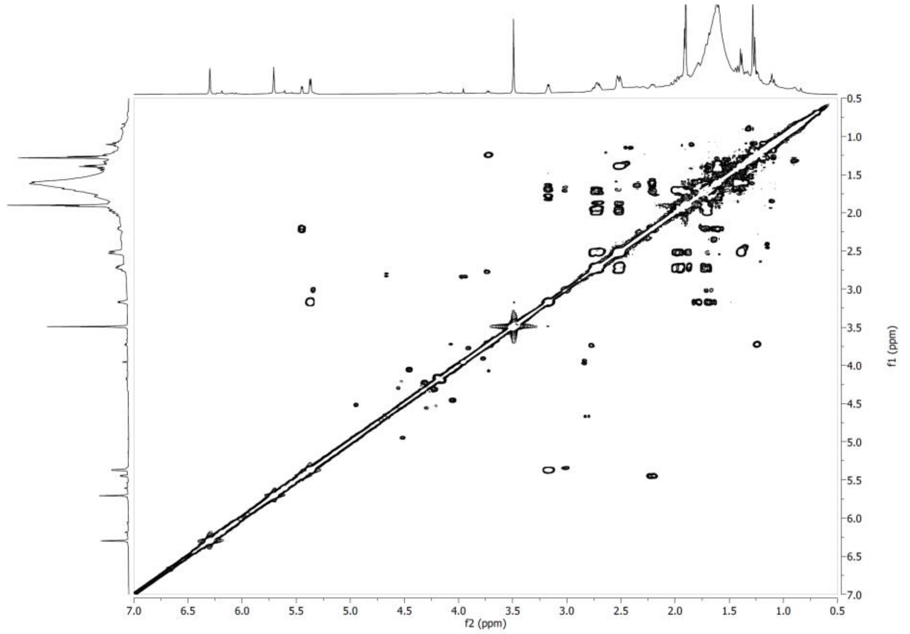

Figure S8: COSY NMR spectrum of compound **Ft_1** in $CDCl_3$

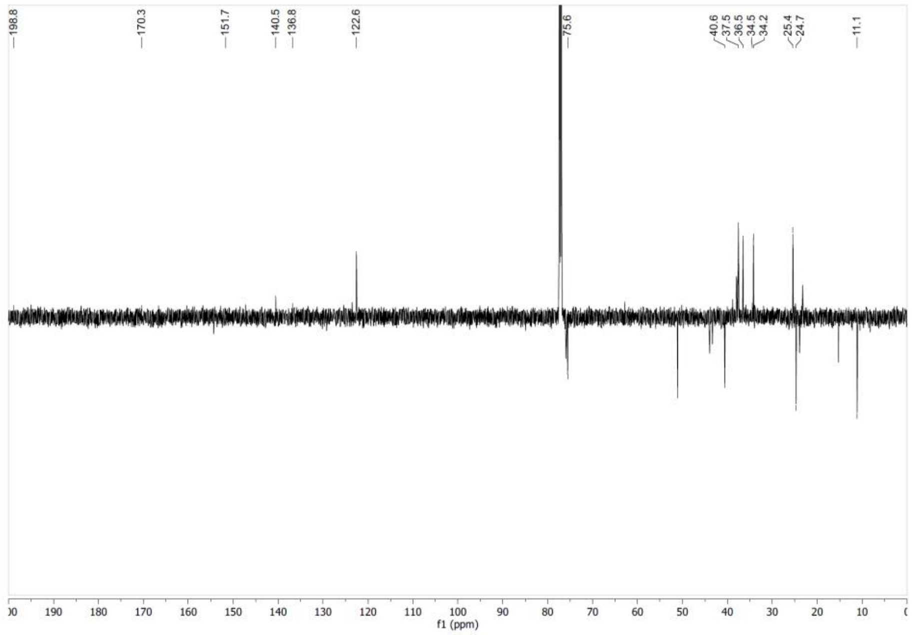

Figure S9: ^{13}C-DEPTQ NMR spectrum of compound **Ft_1** in $CDCl_3$ at 151 MHz

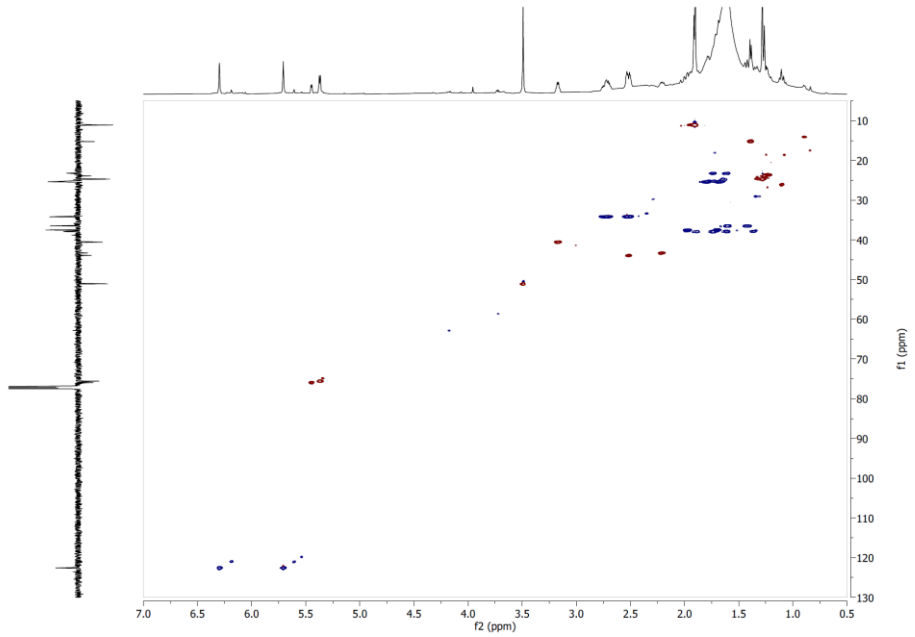

Figure S10: Edited HSQC NMR spectrum of compound **Ft_1** in $CDCl_3$

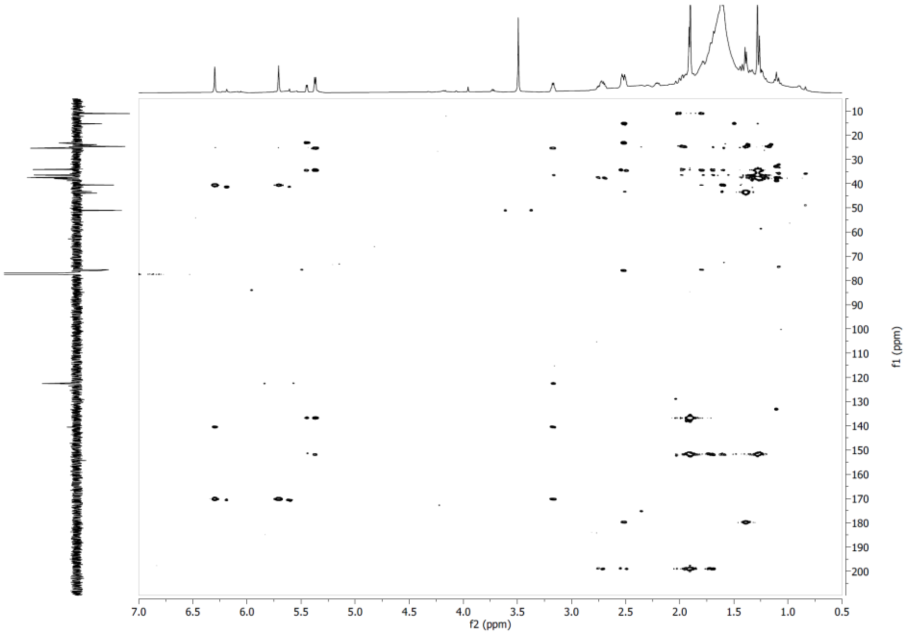

Figure S11: HMBC NMR spectrum of compound **Ft_1** in $CDCl_3$

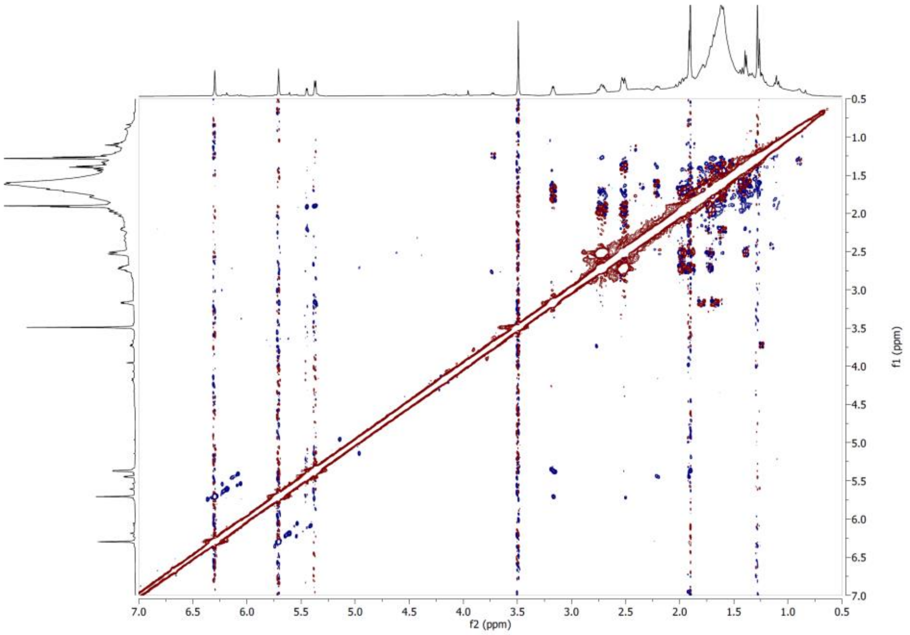

Figure S12: ROESY NMR spectrum of compound **Ft_1** in $CDCl_3$

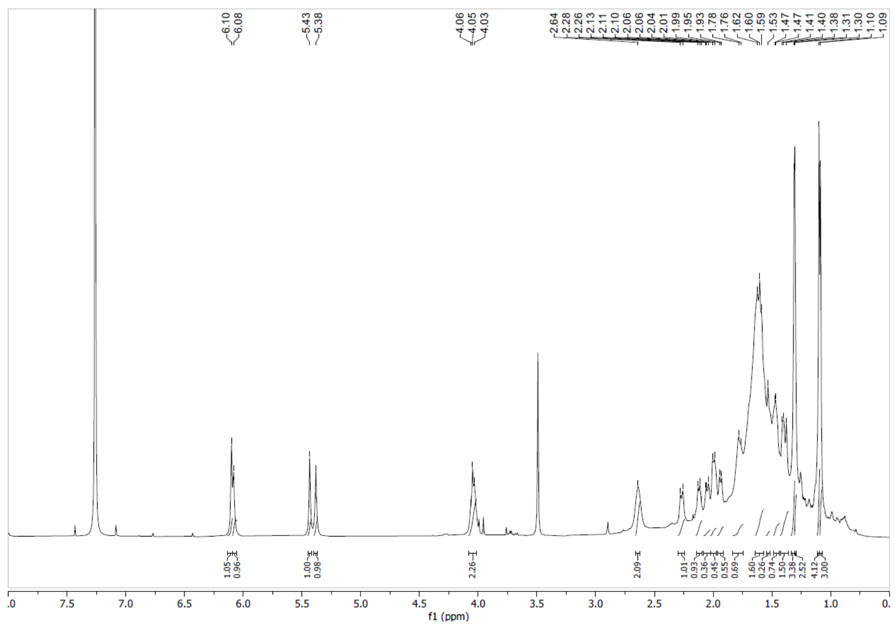

Figure S13: ^{1}H NMR spectrum of compound **Ft_5** in $CDCl_3$ at 600 MHz

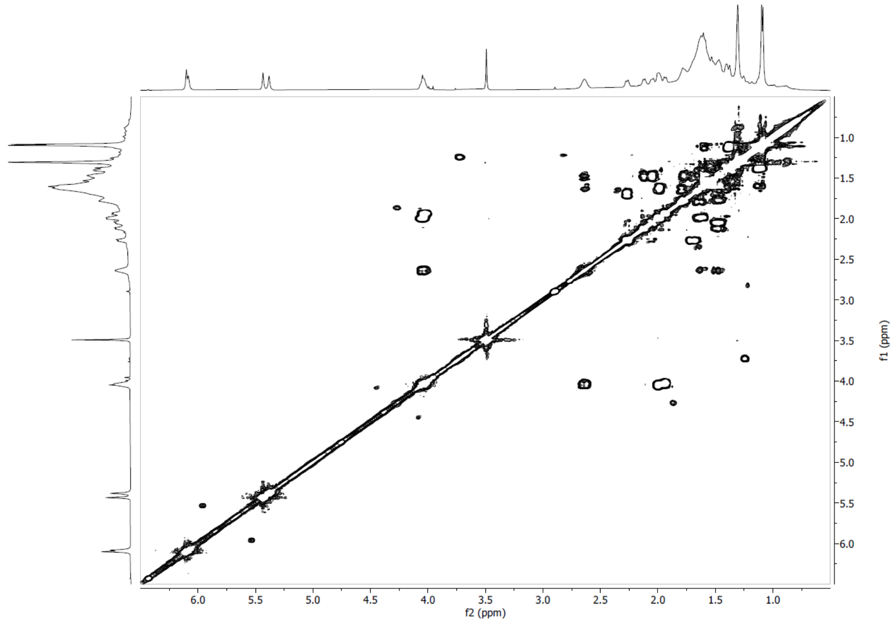

Figure S14: COSY NMR spectrum of compound **Ft_5** in $CDCl_3$

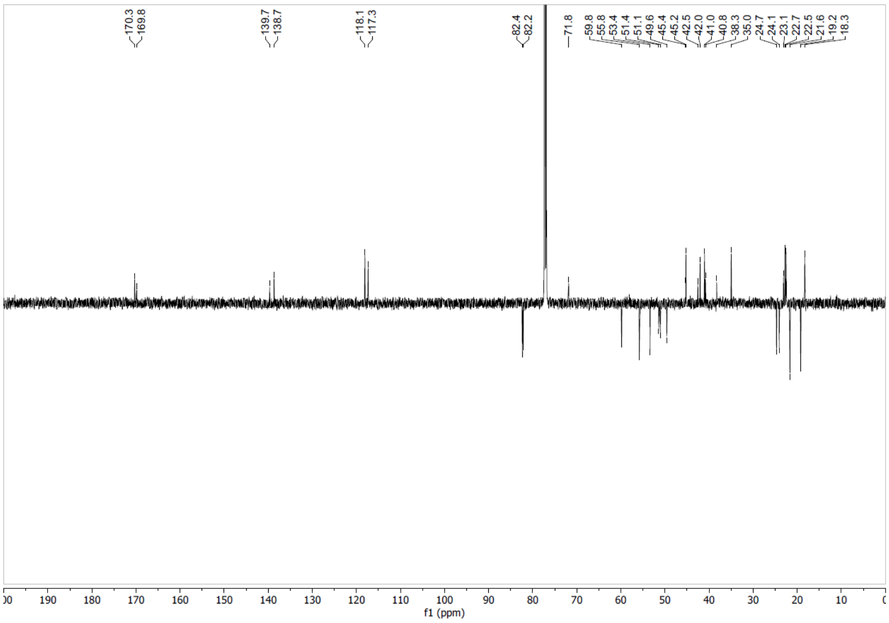

Figure S15: ^{13}C-DEPTQ NMR spectrum of compound **Ft_5** in $CDCl_3$ at 151 MHz

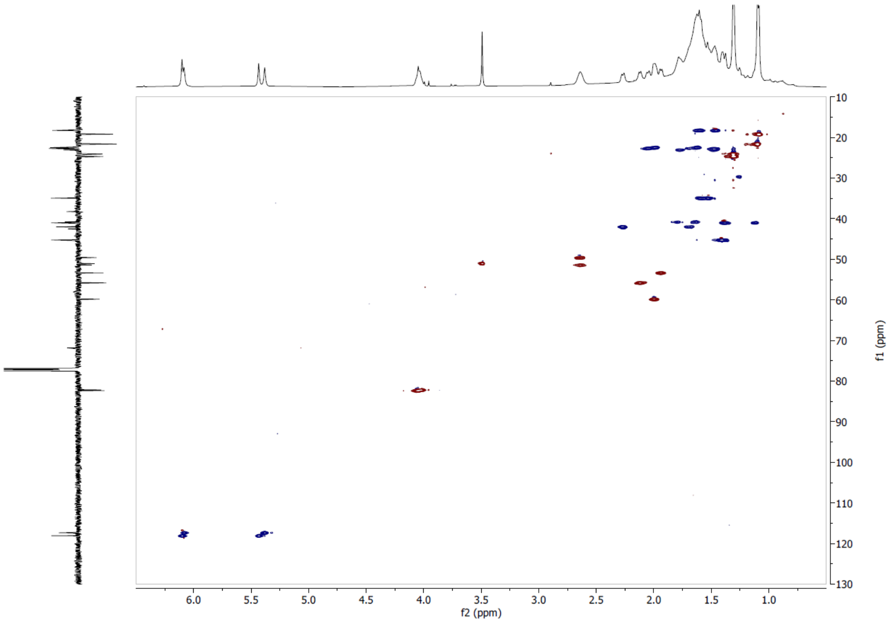

Figure S16: Edited HSQC NMR spectrum of compound **Ft_5** in $CDCl_3$

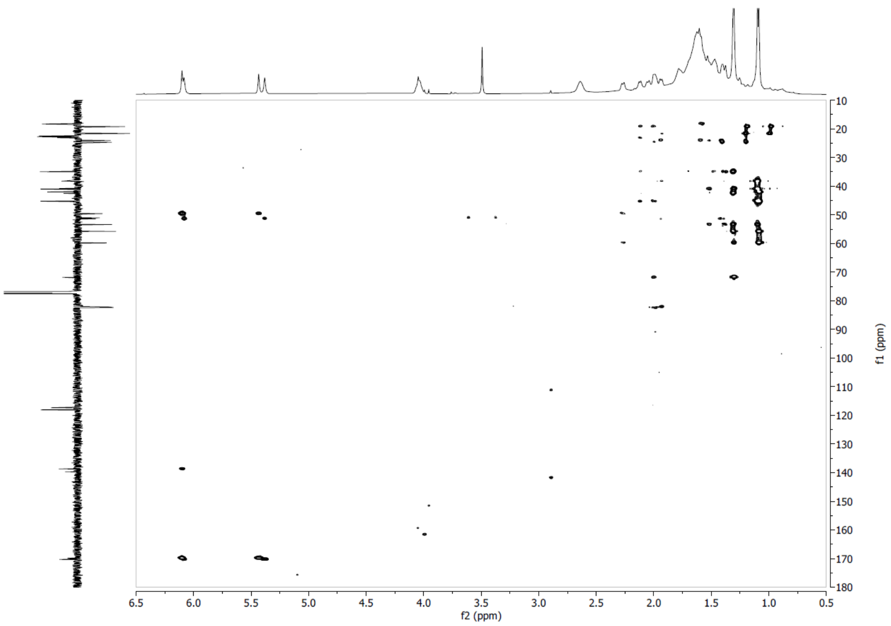

Figure S17: HMBC NMR spectrum of compound **Ft_5** in $CDCl_3$

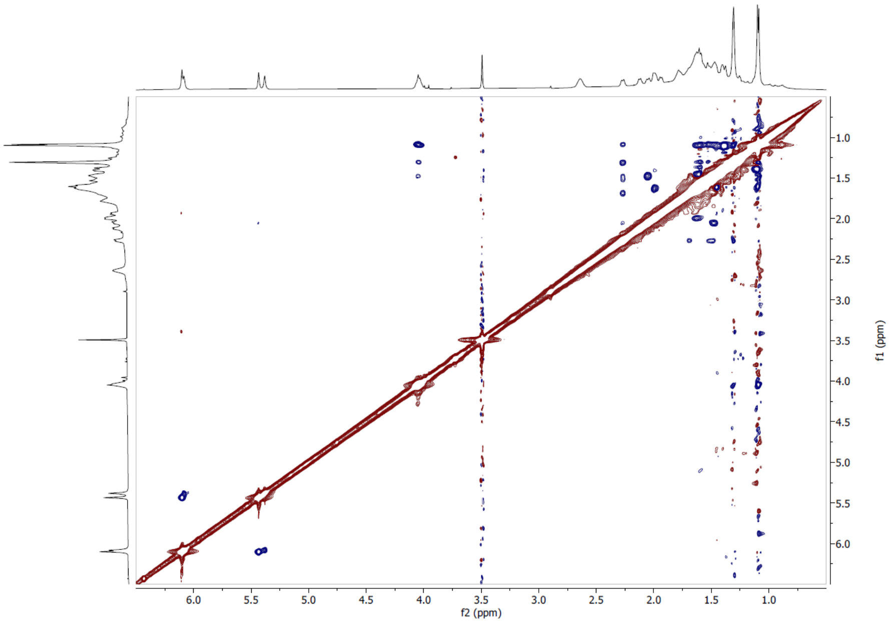

Figure S18: NOESY NMR spectrum of compound **Ft_5** in $CDCl_3$

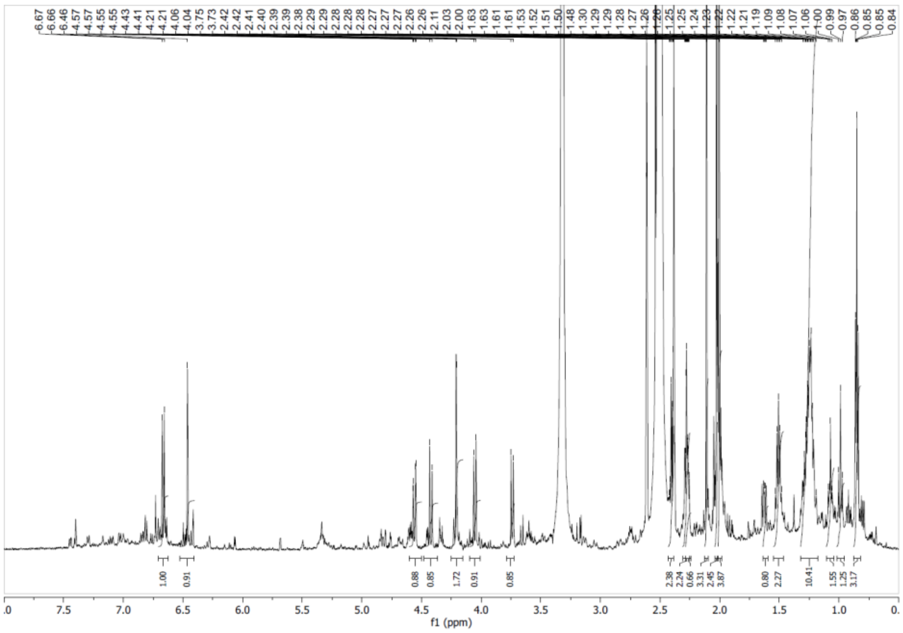

Figure S19: ^{1}H NMR spectrum of compound **Pp_4** in DMSO-d_6 at 600 MHz

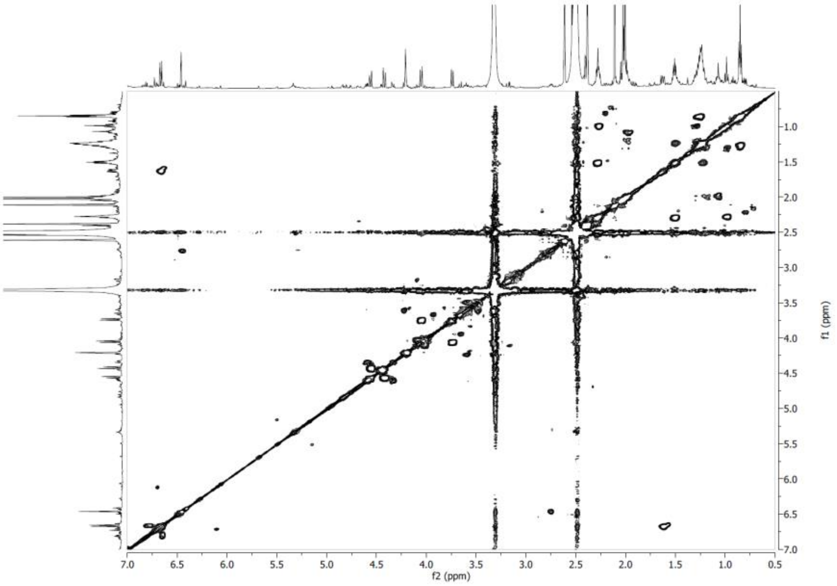

Figure S20: COSY NMR spectrum of compound **Pp_4** in DMSO-d_6

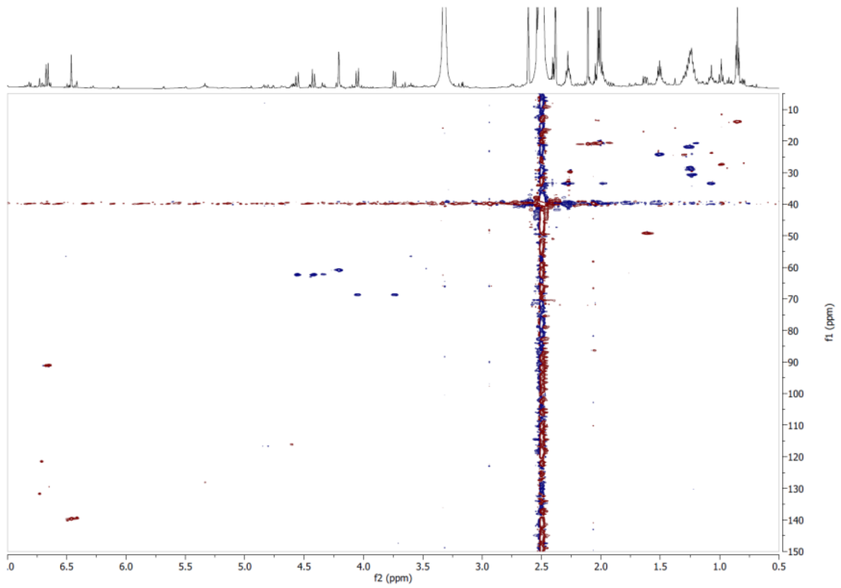

Figure S21: Edited HSQC NMR spectrum of compound **Pp_4** in DMSO-d_6

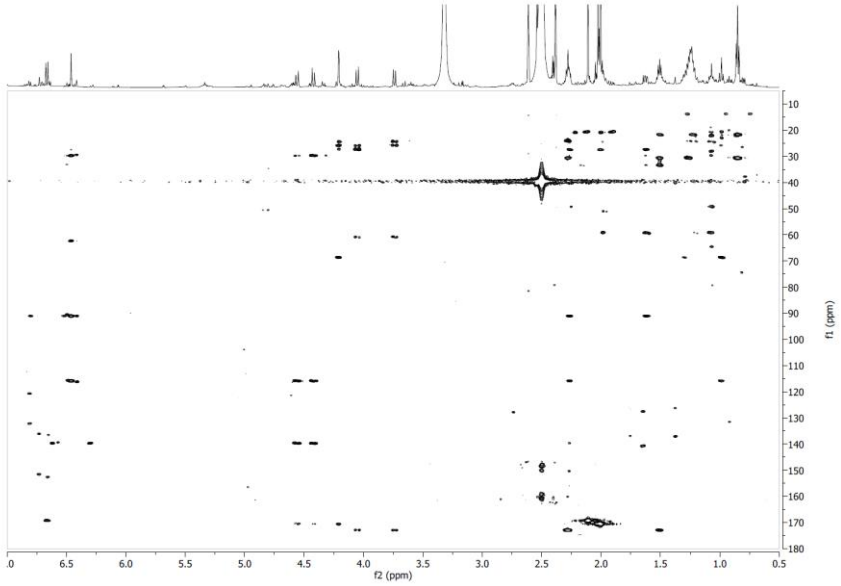

Figure S22: HMBC NMR spectrum of compound **Pp_4** in DMSO-d_6

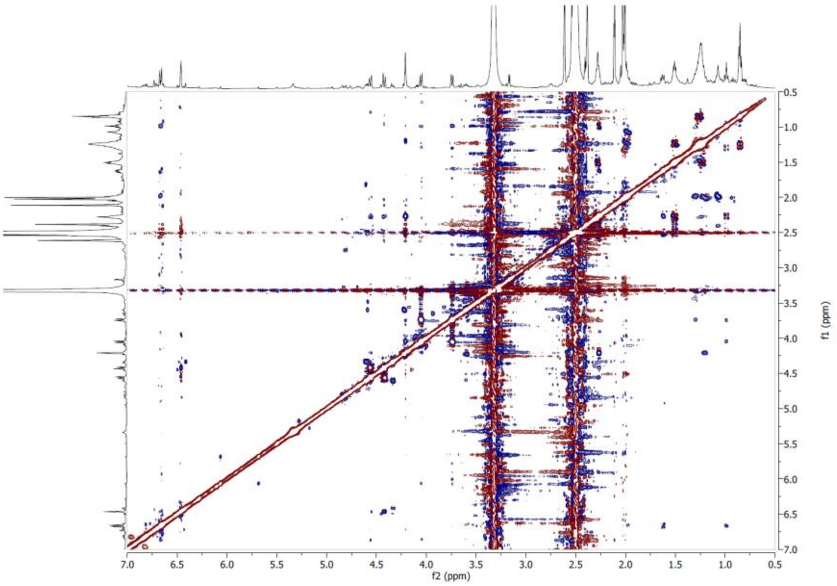

Figure S23: ROESY NMR spectrum of compound **Pp_4** in DMSO-d_6

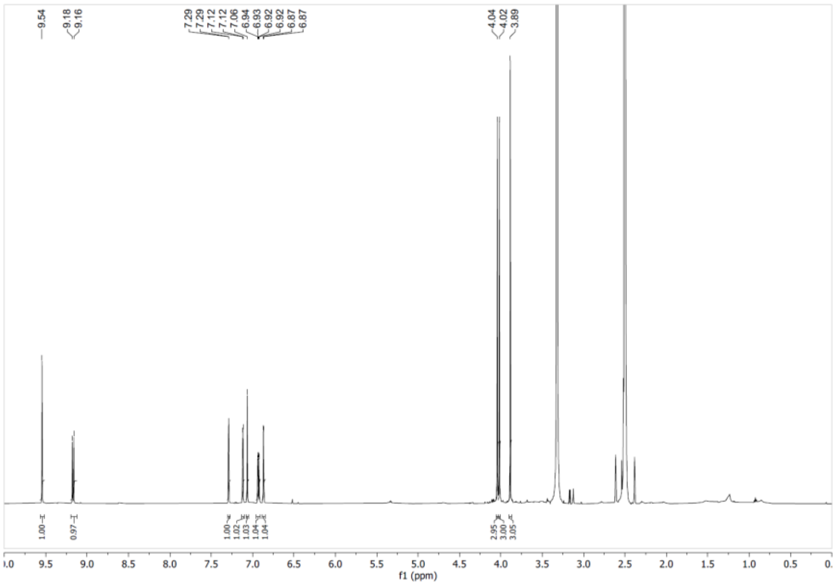

Figure S24: ^{1}H NMR spectrum of compound **Ds_4** in DMSO-d_6 at 600 MHz

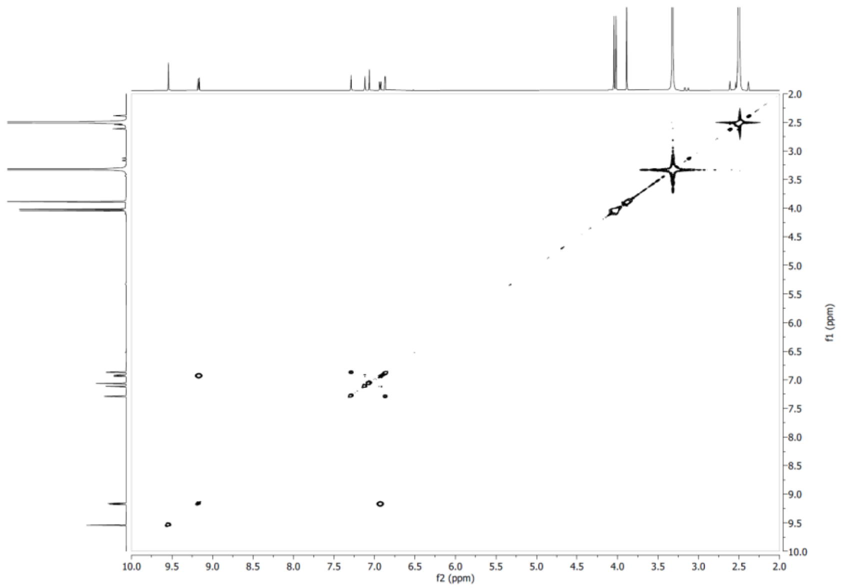

Figure S25: COSY NMR spectrum of compound **Ds_4** in DMSO-d_6

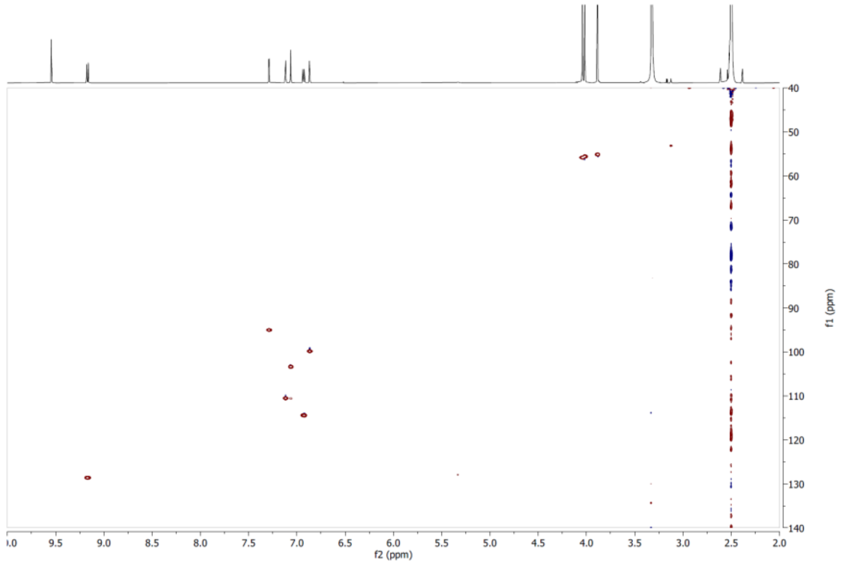

Figure S26: Edited HSQC NMR spectrum of compound **Ds_4** in DMSO-d_6

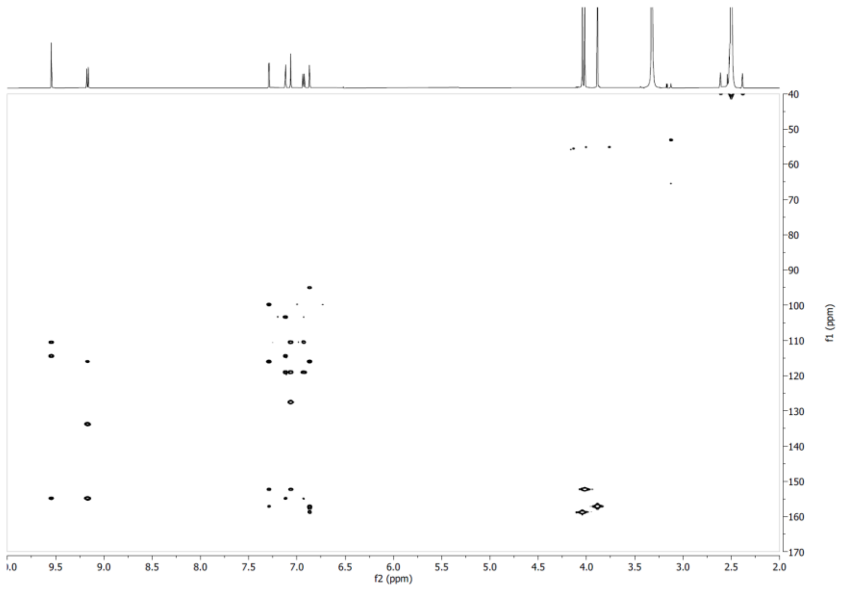

Figure S27: HMBC NMR spectrum of compound **Ds_4** in DMSO-d_6

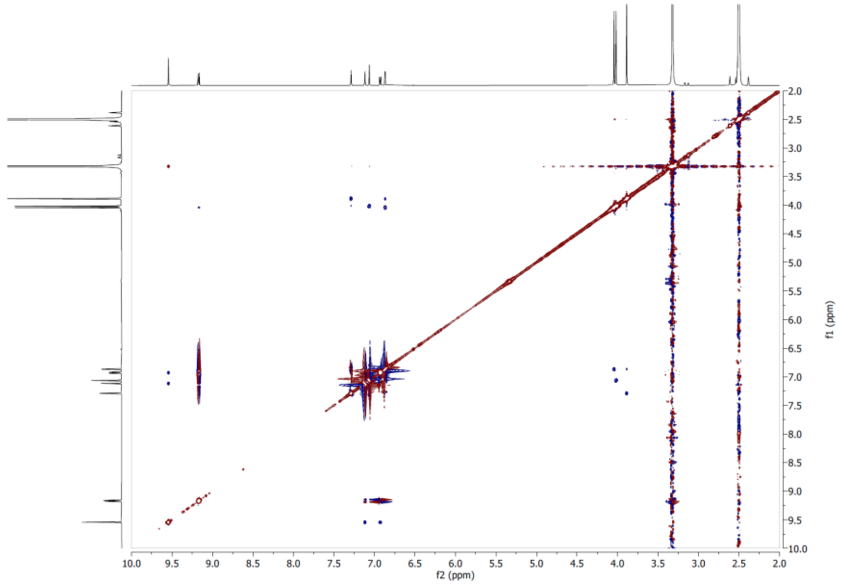

Figure S28: ROESY NMR spectrum of compound **Ds_4** in DMSO-d_6

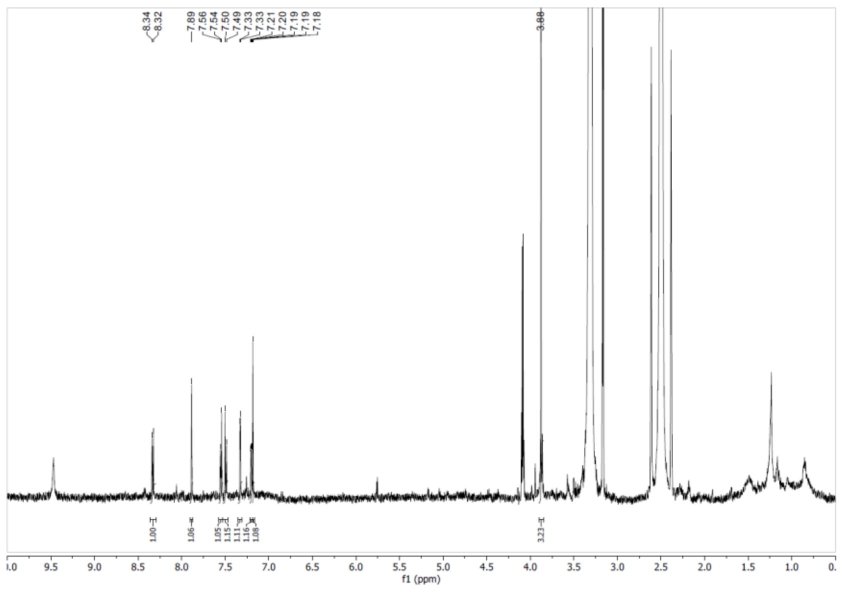

Figure S29: ^{1}H NMR spectrum of compound **Ac_01** in DMSO-d_6 at 600 MHz

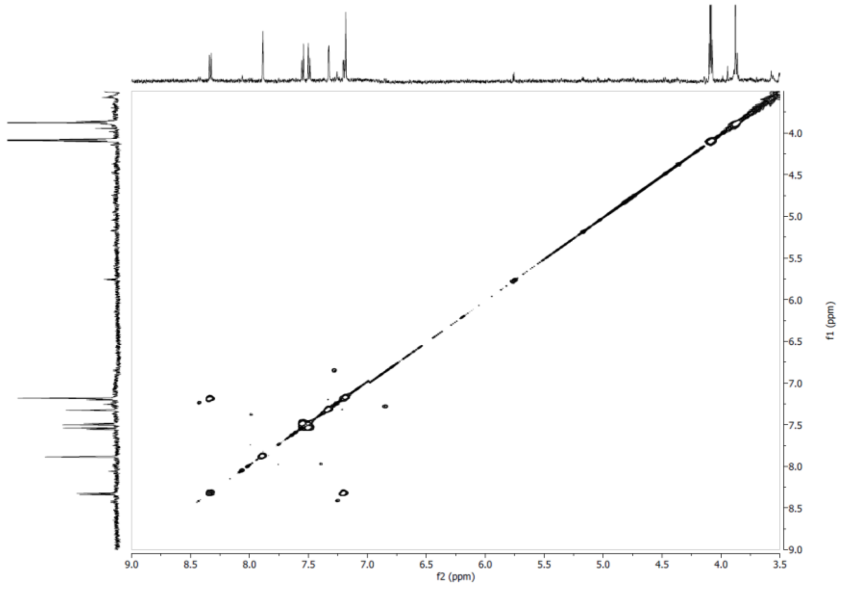

Figure S30: COSY NMR spectrum of compound **Ac_01** in DMSO-d_6

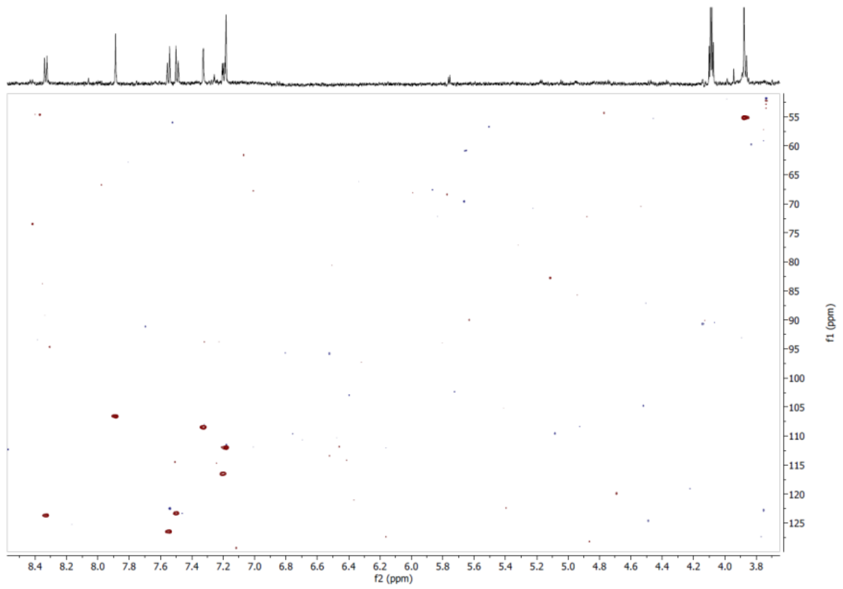

Figure S31: Edited HSQC NMR spectrum of compound **Ac_01** in DMSO-d_6

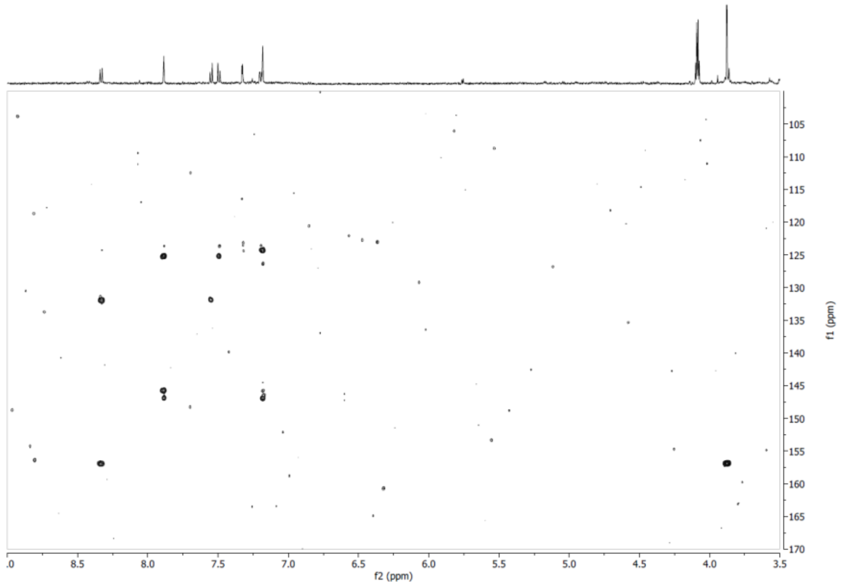

Figure S32: HMBC NMR spectrum of compound **Ac_01** in DMSO-d_6

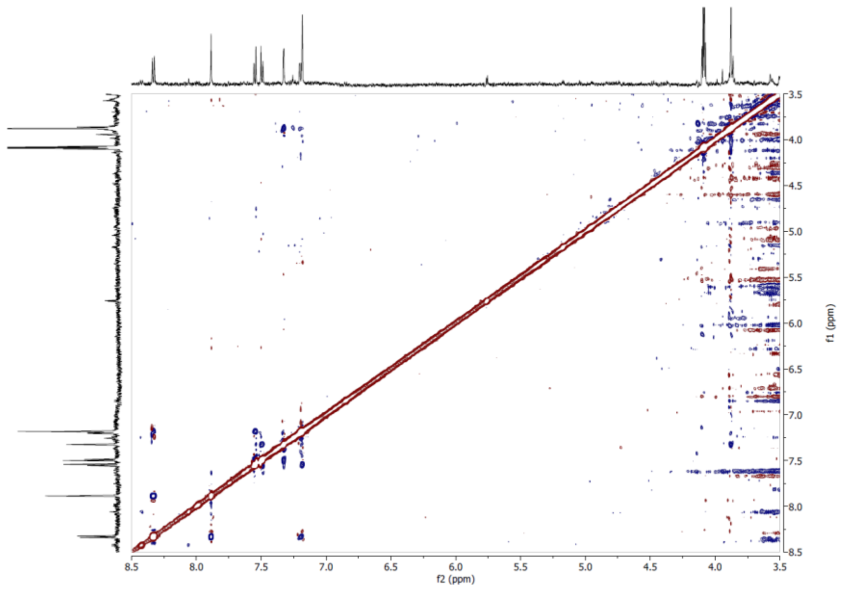

Figure S33: ROESY NMR spectrum of compound **Ac_01** in DMSO-d_6

Supplementary data 1 : NMR data of isolated compounds previously reported in the literature

Ft_2: oxy-frullanolide (Sangsopha et al., 2016) ^{1}H NMR ($CDCl_3$, 600 MHz) δ 0.98 (3H, s, H_3-14), 1.09 (1H, m, H-1eq), 1.11 (1H, m, H-9eq), 1.54 (1H, m, H-8ax), 1.60 (1H, m, H-2eq), 1.69 (1H, m, H-8eq), 1.72 (1H, m, H-2ax), 1.88 (2H, m, H-1ax, H-9ax), 2.20 (1H, dd, *J* = 14.3, 5.0 Hz, H-3eq), 2.54 (1H, td, *J* = 14.3, 5.0 Hz, H-3ax), 3.27 (1H, td, *J* = 12.1, 5.8 Hz, H-7), 4.26 (1H, d, *J* = 4.7 Hz, H-6), 5.02 (1H, s, H-15"), 5.31 (1H, s, H-15'), 5.55 (1H, s, H-13"), 6.09 (1H, s, H-13'); ^{13}C NMR ($CDCl_3$, 151 MHz) δ 18.9 (CH_3-14), 21.9 (CH_2-2), 24.4 (CH_2-8), 31.8 (CH_2-9), 32.2 (CH_2-3), 36.6 (CH_2-1), 37.1 (C-10), 39.1 (CH-7), 74.9 (C-5), 80.2 (CH-6), 111.9 (CH_2-15), 119.6 (CH_2-13), 141.7 (C-11), 147.1 (C-4), 170.8 (C-12).

Ft_3: frullanolide (Chou and Liao, 2013) ^{1}H NMR ($CDCl_3$, 600 MHz) δ 1.07 (3H, s, H_3-14), 1.27 (1H, td, *J* = 12.8, 3.9 Hz, H-9ax), 1.35 (1H, td, *J* = 13.7, 3.6 Hz, H-1ax), 1.43 (1H, dt, *J* = 12.8, 3.6 Hz, H-1eq), 1.48 (1H, ddd, *J* = 13.4, 4.8, 3.4 Hz, H-9eq), 1.64 (2H, m, H-2eq, H-8ax), 1.70 (1H, m, H-8eq), 1.75 (3H, s, H_3-15), 1.82 (1H, tddd, *J* = 13.8, 10.5, 7.0, 3.4 Hz, H-2ax), 2.09 (2H, m, H_2-3), 2.94 (1H, dtt, *J* = 11.9, 6.6, 1.3 Hz, H-7), 5.26 (1H, d, *J* = 5.8 Hz, H-6), 5.57 (1H, d, *J* = 1.3 Hz, H-13"), 6.15 (1H, d, *J* = 1.3 Hz, H-13'); ^{13}C NMR ($CDCl_3$, 151 MHz) δ 18.3 (CH_2-2), 19.5 (CH_3-15), 25.2 (CH_2-8), 26.0 (CH_3-14), 32.8 (C-10), 33.3 (CH_2-3), 38.1 (CH_2-9), 39.3 (CH_2-1), 41.4 (CH-7), 76.1 (CH-6), 120.3 (CH_2-13), 128.7 (C-5), 138.7 (C-4), 142.5 (C-11), 171.1 (C-12).

Ft_4: γ-cyclocostunolide (Kraut et al., 1994) ^{1}H NMR ($CDCl_3$, 600 MHz) δ 1.11 (3H, s, H_3-14), 1.41 (2H, m, H-1ax, H-9ax), 1.59 (5H, m, H-1eq, H-9eq), 1.86 (3H, s, H_3-15), 1.92 (1H, m), 2.03 (2H, m), 2.58 (1H, ddq, *J* = 14.7, 11.6, 3.3 Hz, H-7), 4.53 (1H, d, *J* = 11.6 Hz, H-6), 5.44 (1H, d, *J* = 3.1 Hz, H-13"), 6.13 (1H, d, *J* = 3.2 Hz, H-13').

Ft_6: ursolic acid (Acebey-Castellon et al., 2011): ^{1}H NMR ($CDCl_3$, 600 MHz) δ 0.72 (1H, dd, *J* = 11.8, 1.8 Hz, H-5), 0.78 (3H, s, H_3-23), 0.79 (3H, s, H_3-26), 0.86 (3H, d, *J* = 6.5 Hz, H_3-29), 0.93 (3H, s, H_3-25), 0.95 (3H, d, *J* = 6.3 Hz, H_3-30), 0.99 (3H, s, H_3-24), 1.00 (1H, m, H-1ax), 1.01 (1H, m, H-20), 1.09 (1H, m, 15eq), H_3-27), 1.33 (3H, m, H-7eq, H-19, H-21ax), 1.35 (1H, m H-6ax), 1.48 (1H, m, H-7ax), 1.50 (2H, m, H-9, H-21eq), 1.53 (1H, m, H-6eq), 1.56 (1H, m, H-2’’), 1.63 (1H, m, H-2'), 1.64 (1H, m, H-1eq), 1.65 (1H, m, , H-16eq), 1.66 (1H, m, H-22eq), 1.74 (1H, dt, *J* = 13.1, 3.5 Hz, H-22ax), 1.86 (1H, td, *J* = 13.6, 4.5 Hz, H-15ax), 1.92 (2H, dd, *J* = 8.9, 3.7 Hz, H-11), 2.02 (1H, td, *J* = 13.5, 4.4 Hz, H-16ax), 2.19 (1H, dd, *J* = 11.5, 2.0 Hz, H-18), 3.22 (1H, dd, *J* = 11.3, 4.7 Hz, H-3), 5.26 (1H, t, *J* = 3.7 Hz, H-12); ^{13}C NMR

($CDCl_3$, 151 MHz) δ 15.6 (C-25), 15.7 (C-23), 17.1, 18.4 (C-6), 21.3 (C-30), 23.4 (C-11), 23.7 (C-27), 24.3, 27.4 (C-2), 28.1 (C-15), 28.3 (C-24), 30.7 (C-21), 33.1 (C-7), 36.8 (C-22), 37.1 (C-10), 38.7 (C-1), 38.9 (C-4), 39.0 (C-20), 39.2 (C-19), 39.6 (C-8), 42.2 (C-14), 47.7 (C-9), 52.8 (C-18), 55.4 (C-5), 79.2 (C-3), 126.0 (C-12), 138.1 (C-13), 181.1 (C-28).

Pp_1: Plagiochine D (Asakawa et al., 1979): ^{1}H NMR (DMSO-d_6, 600 MHz) δ 0.99 (1H, t, *J* = 9.7 Hz, H-6), 1.07 (1H, dd, *J* = 13.7, 6.7 Hz, H-9α), 1.20 (1H, m, H-8α), 1.31 (1H, ddd, *J* = 10.3, 9.7, 6.7 Hz, H-7), 1.62 (1H, dd, *J* = 10.2, 3.3 Hz, H-1), 1.99 (1H, overlapped, H-9β), 2.00 (3H, s, H_3-2'), 2.01 (3H, s, H_3-12b), 2.01 (1H, overlapped, H-8β), 2.03 (3H, s, H_3-14b), 2.11 (3H, s, H_3-2b), 2.26 (1H, dd, *J* = 9.7, 3.3 Hz, H-5), 2.40 (2H, AB, H_2-11), 3.74 (1H, d, *J* = 11.4 Hz, H-15''), 4.04 (1H, d, *J* = 11.4 Hz, H-15'), 4.19 (1H, d, *J* = 12.3 Hz, H-14''), 4.22 (1H, d, *J* = 12.3 Hz, H-14'), 4.42 (1H, d, *J* = 12.3 Hz, H-12''), 4.57 (1H, dd, *J* = 12.3, 1.3 Hz, H-12'), 6.46 (1H, s, H-3), 6.66 (1H, d, *J* = 10.2 Hz, H-2); ^{13}C NMR (DMSO-d_6, 151 MHz) δ 20.6 (CH_3-12b), 20.6 (CH_3-2'), 20.7 (CH_2-8), 20.7 (CH_3-14b), 20.9 (CH_3-2b), 24.4 (CH-7), 25.9 (C-13), 27.4 (CH-6), 29.7 (CH-5), 33.4 (CH_2-9), 49.1 (CH-1), 59.2 (C-10), 60.9 (CH_2-14), 62.3 (CH_2-12), 68.8 (CH_2-15), 91.1 (CH-2), 115.8 (C-4), 139.6 (CH-3), 169.2 (C-2a), 170.3 (C-1'), 170.5 (C-12a), 170.5 (C-14a).

Pp_2: Plagiochiline R-15-yl octanoate (Toyota et al., 1994; Ramírez et al., 2017) ^{1}H NMR (DMSO-d_6, 600 MHz) δ 0.85 (3H, t, *J* = 7.0 Hz, H_3-8'), 0.99 (1H, t, *J* = 9.6 Hz, H-6), 1.07 (1H, dd, *J* = 14.0, 6.7 Hz, H-9α), 1.20 (1H, m, H-8α), 1.25 (8H, m, H_2-4', H_2-5', H_2-6', H_2-7'), 1.30 (1H, m, H-7), 1.50 (2H, p, *J* = 7.0 Hz, H_2-3'), 1.62 (1H, dd, *J* = 10.2, 3.2 Hz, H-1), 1.98 (1H, m, H-9β), 2.00 (3H, s, H_3-14b), 2.00 (1H, overlapped, H-8β), 2.02 (3H, s, H_3-12b), 2.11 (3H, s, H_2-2b), 2.27 (1H, dd, *J* = 9.6, 3.2 Hz, H-5), 2.28 (2H, td, *J* = 7.4, 2.2 Hz, H_2-2'), 2.40 (1H, AB, H-11), 3.74 (1H, d, *J* = 11.4 Hz, H-15''), 4.05 (1H, d, *J* = 11.4 Hz, H-15'), 4.21 (2H, AB, H_2-14), 4.42 (1H, d, *J* = 12.4 Hz, H-12''), 4.56 (1H, d, *J* = 12.3 Hz, H-12'), 6.46 (1H, s, H-3), 6.67 (1H, d, *J* = 10.2 Hz, H-2); ^{13}C NMR (DMSO-d_6, 151 MHz) δ 14.0 (CH_3-8'), 20.5 (CH_3-14b), 20.6 (CH_2-8), 20.7 (CH_3-12b), 20.9 (CH_3-2b,), 22.1 (CH_2-7'), 24.3 (CH-7), 24.5 (CH_2-3'), 25.7 (C-13), 27.3 (CH-6), 28.4 (CH_2-4', CH_2-5'), 29.8 (CH-5), 31.2 (CH_2-6'), 33.2 (CH_2-9), 33.5 (CH_2-2'), 49.1 (CH-1), 51.2 (CH_2-11), 59.2 (C-10), 60.9 (CH_2-14), 62.3 (CH_2-12), 68.6 (CH_2-15), 91.1 (CH-2), 115.8 (C-4), 139.7 (CH-3), 169.2 (C-2a), 170.4 (C-12a), 170.6 (C-14a), 172.8 (C-1').

Pp_3: Plagiochiline R-15-yl dec-4-enoate (Toyota et al., 1994; Ramírez et al., 2017) ^{1}H NMR (DMSO-d_6, 600 MHz) δ 0.85 (3H, t, *J* = 7.0 Hz, H_3-10'), 0.99 (1H, t, *J* = 9.7 Hz, H-6), 1.07 (1H, dd, *J* = 14.5, 6.9 Hz, H-9α), 1.23 (2H, m, H_2-8'), 1.27 (4H, m, H_2-7', H_2- 9'), 1.30 (1H, m,

H-7), 1.62 (1H, dd, *J* = 10.3, 2.9 Hz, H-1), 1.98 (3H, m, H_2-6', H-9β), 2.00 (3H, s, H_3-14b), 2.00 (1H, overlapped, H-8β), 2.02 (3H, s, H_3-12b), 2.11 (3H, s, H_3-2b), 2.24 (2H, m, H_2-3'), 2.27 (1H, dd, *J* = 9.7, 2.9 Hz, H-5), 2.32 (2H, m, H_2-2'), 2.40 (2H, AB, H_2-11), 3.74 (1H, d, *J* = 11.4 Hz, H-15"), 4.05 (1H, d, *J* = 11.4 Hz, H-15'), 4.21 (2H, AB, H_2-14), 4.42 (1H, d, *J* = 12.4 Hz, H-12"), 4.56 (1H, d, *J* = 12.4 Hz, H-12'), 5.29 (1H, dt, *J* = 10.5, 7.3 Hz, 4'), 5.36 (1H, dt, *J* = 10.5, 7.3 Hz, 5'), 6.46 (1H, s, H-3), 6.66 (1H, d, *J* = 10.3 Hz, H-2); ^{13}C NMR (DMSO-d_6, 151 MHz) δ 14.0 (CH_3-10’), 20.6 (CH_3-14b, CH_2-8), 20.7 (CH_3-12b), 20.9 (CH_3-2b,), 21.8 (CH_2-9’), 22.4 (CH_2-3’), 24.3 (CH-7), 25.7 (C-13), 26.4 (CH_2-6’), 27.3 (CH-6), 28.6 (CH_2-7’), 29.8 (CH-5), 31.0 (CH_2-8’), 33.3 (CH_2-9), 33.4 (CH_2-2’), 49.1 (CH-1), 50.9 (CH_2-11), 59.2 (C-10), 60.9 (CH_2-14), 62.3 (CH_2-12), 68.8 (CH_2-15), 91.1 (CH-2), 115.8 (C-4), 127.7 (CH-4’), 130.9 (CH-5’), 139.7 (CH-3), 169.2 (C-2a), 170.4 (C-12a), 170.6 (C-14a), 172.4 (C-1’).

Pp_5: Diplophylin (Ohta et al., 1977) ^{1}H NMR (DMSO-d_6, 600 MHz) δ 1.02 (3H, s, H_3-14), 1.40 (1H, dt, *J* = 12.6, 4.0 Hz, H-1β), 1.45 (1H, td, *J* = 12.6, 4.0 Hz, H-1α), 1.55 (1H, m, H-2"), 1.63 (3H, s, H_3-15), 1.63 (1H, overlapped, H-2’), 1.66 (2H, m, H_2-9), 1.83 (1H, dd, *J* = 13.5, 12.5 Hz, H-6β), 1.92 (2H, m, H_2-3), 2.83 (1H, dd, *J* = 13.5, 7.4 Hz, H-6α), 3.10 (1H, ddd, *J* = 12.5, 7.4, 6.9 Hz, H-7), 4.52 (1H, q, *J* = 6.9 Hz, H-8), 5.77 (1H, d, *J* = 2.4 Hz, H-13"), 6.07 (1H, d, *J* = 2.4 Hz, H-13') ; ^{13}C NMR (DMSO-d_6, 151 MHz) δ 18.3 (CH_2-2), 19.0 (CH_3-15), 26.5 (CH_3-14), 27.2 (CH_2-6), 31.4 (CH_2-3), 33.0 (C-10), 36.5 (CH_2-1), 40.2 (CH-7), 42.1 (CH_2-9), 75.8 (CH-8), 121.5 (CH_2-13), 126.1 (C-4), 131.5 (C-5), 140.4 (C-11), 170.0 (C-12).

Pe_1: 10-hydroxyperrottetin E (Cullmann et al., 1997) ^{1}H NMR (DMSO-d_6, 600 MHz) δ 2.68 (2H, m, H_2-7'), 2.72 (1H, m, H_2-8'), 2.76 (2H, m, H_2-8), 2.79 (2H, m, H_2-7), 6.45 (1H, dd, *J* = 7.6, 1.7 Hz, H-14'), 6.49 (1H, t, *J* = 7.6 Hz, H-13'), 6.57 (1H, dd, *J* = 8.0, 2.2 Hz, H-12), 6.60 (1H, dd, *J* = 7.6, 1.7 Hz, H-12'), 6.63 (1H, d, *J* = 2.2 Hz, H-10), 6.64 (1H, d, *J* = 8.0 Hz, H-14), 6.72 (2H, d, *J* = 8.3 Hz, H-2, H-6), 6.73 (1H, d, *J* = 2.3 Hz, H-3'), 6.84 (1H, d, *J* = 8.4 Hz, H-6'), 6.86 (1H, dd, *J* = 8.4, 2.3 Hz, H-5'), 7.05 (1H, t, *J* = 8.0 Hz, H-13), 7.14 (2H, d, *J* = 8.3 Hz, H-3, H-5), 8.08 (1H, s, 10'OH), 9.11 (1H, s, 11'OH), 9.18 (1H, s, 1'OH), 9.22 (1H, s, 11OH); ^{13}C NMR (DMSO-d_6, 151 MHz) δ 32.1 (CH_2-8'), 34.5 (CH_2-7'), 36.2 (CH_2-7), 37.3 (CH_2-8), 112.8 (CH-12), 113.1 (CH-12'), 115.2 (CH-10), 116.0 (CH-2, CH-6), 117.0 (CH-6'), 118.5 (CH-13'), 119.0 (CH-14), 120.4 (CH-14'), 121.4 (CH-3'), 124.8 (CH-5'), 128.3 (C-9'), 129.1 (CH-13), 129.3 (CH-3, CH-5), 133.5 (C-4'), 135.0 (C-4), 142.3 (C-2'), 143.0 (C-9), 143.0 (C-10'), 144.8 (C-11'), 147.0 (C-1'), 156.1 (C-1), 157.3 (C-11).

Pe_2: Perrottetin E (Cullmann et al., 1997) ^{1}H NMR (DMSO-d_6, 600 MHz) δ 2.71 (4H, s, H_2-7', H_2-8'), 2.75 (2H, m, H_2-8), 2.78 (2H, m, H_2-7), 6.54 (1H, d, *J* = 1.4 Hz, H-10'), 6.57 (3H, m,

H-12, H-12', H-14'), 6.63 (1H, t, *J* = 2.4 Hz, H-10), 6.64 (1H, d, *J* = 7.8 Hz, H-14), 6.70 (2H, d, *J* = 8.6 Hz, H-2, H-6), 6.73 (1H, d, *J* = 1.9 Hz, H-3'), 6.84 (1H, d, *J* = 8.2 Hz, H-6'), 6.86 (1H, dd, *J* = 8.2, 1.9 Hz, H-5'), 7.01 (1H, dd, *J* = 8.8, 7.5 Hz, H-13'), 7.05 (1H, t, *J* = 7.8 Hz, H-13), 7.13 (2H, d, *J* = 8.6 Hz, H-3, H-5), 9.18 (1H, s, 11'OH), 9.19 (1H, s, 1'OH), 9.21 (1H, s, 11OH); ^{13}C NMR (DMSO-d_6, 151 MHz) δ 36.0 (CH_2-7'), 36.2 (CH_2-7), 37.3 (CH_2-8'), 37.3 (CH_2-8), 112.7 (CH-12), 112.8 (CH-12'), 115.2 (CH-10), 115.3 (CH-10'), 116.0 (CH-2, CH-6), 117.0 (CH-6'), 119.0 (CH-14), 119.1 (CH-14'), 121.6 (CH-3'), 124.9 (CH-5'), 129.0 (CH-13'), 129.1 (CH-13), 129.3 (CH-3, CH-5), 132.9 (C-4'), 134.9 (C-4), 142.3 (C-2'), 142.8 (C-9'), 143.0 (C-9), 147.1 (C-1'), 156.1 (C-1), 157.2 (C-11'), 157.3 (C-11).

Pe_3: 10-hydroxy-11-methoxy-perrottetin E (Cullmann et al., 1997) ^{1}H NMR (DMSO-d_6, 600 MHz) δ 2.67 (2H, m, H_2-7'), 2.71 (2H, m, H_2-8'), 2.81 (4H, s, H_2-7, H_2-8), 3.71 (3H, s, 11OCH$_3$), 6.45 (1H, dd, *J* = 7.6, 1.7 Hz, H-14'), 6.49 (1H, t, *J* = 7.8 Hz, H-13'), 6.60 (1H, dd, *J* = 7.8, 1.7 Hz, H-12'), 6.72 (4H, m, H-2, H-3', H-6, H-12), 6.78 (1H, t, *J* = 1.2 Hz, H-10), 6.80 (2H, dt, *J* = 7.7, 1.2 Hz, H-14), 6.84 (1H, d, *J* = 8.2 Hz, H-6'), 6.86 (1H, dd, *J* = 8.2, 1.9 Hz, H-5'), 7.14 (2H, d, *J* = 8.5 Hz, H-3, H-5), 7.17 (1H, t, *J* = 7.8 Hz, H-13), 8.08 (1H, s, 10'OH), 9.11 (1H, s, 11'OH), 9.18 (1H, s, 1'OH); ^{13}C NMR (DMSO-d_6, 151 MHz) δ 32.0 (CH_2-8'), 34.5 (CH_2-7'), 36.2 (CH_2-7), 37.3 (CH_2-8), 54.9, 111.3 (CH-12), 113.1 (CH-12'), 114.0 (CH-10), 116.0 (CH-2, CH-6), 117.0 (CH-6'), 118.5 (CH-13'), 120.3 (CH-14'), 120.6 (CH-14), 121.4 (CH-3'), 124.8 (CH-5'), 128.3 (C-9'), 129.2 (CH-13), 129.3 (CH-3, CH-5), 133.5 (C-4'), 134.9 (C-4), 142.3 (C-2'), 143.0 (C-10'), 143.2 (C-9), 144.8 (C-11'), 147.0 (C-1'), 156.1 (C-1), 159.2 (C-11).

Pe_4: 11-methoxy-perrottetin E (Cullmann et al., 1997) ^{1}H NMR (DMSO-d_6, 600 MHz) δ 2.70 (4H, s, H_2-7', H_2-8'), 2.81 (4H, s, H_2-7, H_2-8), 3.71 (3H, s, 11OCH$_3$), 6.55 (2H, m, H-10', H-12'), 6.57 (1H, dt, *J* = 7.4, 1.4 Hz, H-14'), 6.70 (4H, d, *J* = 8.6 Hz, H-2, H-6), 6.72 (1H, d, *J* = 2.0 Hz, H-3'), 6.73 (1H, dd, *J* = 7.8, 2.0 Hz, H-12), 6.78 (1H, t, *J* = 2.0 Hz, H-10), 6.80 (1H, d, *J* = 7.8 Hz, H-14), 6.84 (1H, d, *J* = 8.3 Hz, H-6'), 6.86 (1H, dd, *J* = 8.3, 1.9 Hz, H-5'), 7.01 (1H, dd, *J* = 8.8, 7.4 Hz, H-13'), 7.14 (2H, d, *J* = 8.6 Hz, H-3, H-5), 7.18 (1H, t, *J* = 7.8 Hz, H-13), 9.18 (1H, s, 11'OH), 9.19 (1H, s, 1'OH); ^{13}C NMR (DMSO-d_6, 151 MHz) δ 32.0 (CH_2-8'), 36.1 (CH_2-7, CH_2-7'), 37.1 (CH_2-8'), 37.3 (CH_2-8), 54.9 (11OCH$_3$), 111.3 (CH-12), 112.7 (CH-12'), 114.0 (CH-10), 115.4 (CH-10'), 116.1 (CH-2, CH-6), 116.9 (CH-6'), 119.1 (CH-14'), 120.7 (CH-14), 121.7 (CH-3'), 124.9 (CH-5'), 129.1 (CH-13'), 129.2 (CH-13), 129.4 (CH-3, CH-5), 132.8 (C-4'), 134.9 (C-4), 142.3 (C-1'), 142.9 (C-9'), 143.2 (C-9), 147.2 (C-2'), 156.2 (C-1), 157.3 (C-11'), 159.3 (C-11).

Ds_1: Apigenin 7-*O*-[2,4-di-*O*-(α-L-rhamnopyranosyl)]-β-D-glucopyranoside (Becker et al., 1986) ^{1}H NMR (CD_3OD, 600 MHz) δ 1.29 (3H, d, *J* = 6.2 Hz, Rha4-6'), 1.33 (3H, d, *J* = 6.2 Hz, Rha2-6), 3.41 (1H, t, *J* = 9.7 Hz, Rha2-4'), 3.43 (1H, t, *J* = 9.5 Hz, Rha4-4'), 3.60 (1H, dd, *J* = 9.6, 3.4 Hz, Rha2-3'), 3.65 (1H, m, Rha4-3'), 3.64 (1H, m, Glc-5'), 3.66 (1H, m, Glc-4'), 3.70 (1H, dd, *J* = 12.5, 8.7 Hz, Glc-6'b), 3.74 (1H, m, Glc-2'), 3.76 (1H, m, Glc-3'), 3.87 (1H, m, Rha4-2'), 3.88 (1H, dd, *J* = 12.5, 1.8 Hz, Glc-6'a), 3.92 (1H, dq, *J* = 9.7, 6.2 Hz, Rha2-5'), 3.96 (1H, dd, *J* = 3.4, 1.8 Hz, Rha2-2'), 3.99 (1H, dq, *J* = 9.5, 6.2 Hz, Rha4-5'), 4.89 (1H, d, *J* = 1.7 Hz, Rha4-1'), 5.25 (1H, d, *J* = 6.8 Hz, Glc-1'), 5.30 (1H, d, *J* = 1.8 Hz, Rha2-1'), 6.47 (1H, d, *J* = 2.1 Hz, H-6), 6.67 (1H, s, H-3), 6.79 (1H, d, *J* = 2.1 Hz, H-8), 6.94 (2H, d, *J* = 8.4 Hz, H-3', H-5'), 7.89 (2H, d, *J* = 8.4 Hz, H-2', H-6'); ^{13}C NMR (CD_3OD, 151 MHz) δ 17.9 (Rha4-6'), 18.3 (Rha2-6'), 61.6 (Glc-6'), 70.0 (Rha2-5'), 70.7 (Rha4-5'), 72.2 (Rha2-3', Rha4-3'), 72.2 (Rha2-2'), 72.4 (Rha4-2'), 73.7 (Rha4-4'), 73.9 (Rha2-4), 77.1 (Glc-5'), 77.6 (Glc-3'), 79.2 (Glc-4'), 79.4 (Glc-2'), 95.9 (CH-8), 99.5 (Glc-1'), 100.9 (CH-6), 102.7 (Rha2-1'), 103.0 (Rha4-1'), 104.1 (CH-3), 107.1 (C-10), 117.1 (C-3', C-5'), 122.9 (C-1'), 129.7 (C-2', C-6'), 159.0 (C-9), 163.0 (C-5), 163.3 (C-4'), 164.3 (C-7), 166.9 (C-2), 184.1 (C-4).

Ds_2: 7-[(*O*-6-Deoxy-α-L-mannopyranosyl-(1→2)-*O*-[6-deoxy-α-L-mannopyranosyl-(1→4)]-β-D-glucopyranosyl)oxy]-5-hydroxy-2-(3-hydroxy-4-methoxyphenyl)-4*H*-1-benzopyran-4-one (Osterdahl, 1978) ^{1}H NMR (DMSO-d_6, 600 MHz) δ 1.13 (3H, d, *J* = 6.1 Hz, Rha4-6'), 1.20 (3H, d, *J* = 6.3 Hz, Rha2-6), 3.21 (2H, m, Rha2-4', Rha4-4'), 3.33 (1H, overlapped, Rha2-3'), 3.44 (1H, m, Rha4-3'), 3.48 (1H, m, Glc-6'b), 3.63-3.53 (3H, m, Glc-2', Glc-3', Glc-4', Glc-5'), 3.64 (1H, m, Rha4-2'), 3.66 (11H, m, Glc-6'a), 3.71 (1H, m, Rha2-5'), 3.72 (Rha2-2'), 3.88 (3H, s, 4'OCH_3), 3.91 (1H, m, Rha4-5'), 4.46 (1H, d, *J* = 6.0 Hz, OHRha2-3'), 4.51 (1H, d, *J* = 5.9 Hz, OHRha4-3'), 4.68 (each, 1H, d, *J* = 4.9 Hz, OHRha2-2', OHRha4-4'), 4.70 (1H, d, *J* = 4.9 Hz, OHRha2-4'), 4.72 (1H, d, *J* = 4.3 Hz, OHRha4-2'), 4.73 (1H, d, *J* = 1.7 Hz, Rha4-1'), 4.87 (1H, dd, *J* = 6.5, 4.9 Hz, OHGlc-6'), 5.11 (1H, d, *J* = 1.7 Hz, Rha2-1'), 5.18 (1H, d, *J* = 5.0 Hz, OHGlc-3'), 5.32 (1H, d, *J* = 7.2 Hz, Glc-1'), 6.38 (1H, d, *J* = 2.2 Hz, H-6), 6.75 (1H, d, *J* = 2.2 Hz, H-8), 6.85 (1H, s, H-3), 7.11 (1H, d, *J* = 8.6 Hz, H-5'), 7.44 (1H, d, *J* = 2.3 Hz, H-2'), 7.57 (1H, dd, *J* = 8.6, 2.3 Hz, H-6'), 9.48 (1H, s, 3'OH), 12.96 (1H, s, 5OH); ^{13}C NMR (DMSO-d_6, 151 MHz) δ 17.8 (Rha4-6'), 18.2 (Rha2-6'), 55.8 (OCH_3), 59.7 (Glc-6'), 68.5 (Rha2-5'), 68.6 (Rha4-5'), 70.4 (Rha2-2', Rha2-3'), 70.6 (Rha4-3'), 70.8 (Rha4-2'), 72.0 (Rha2-4', Rha4-4'), 75.6 (Glc-4', Glc-5'), 77.1 (Glc-2', Glc-3'), 94.3 (CH-8), 97.5 (Glc-1'), 99.2 (CH-6), 100.5 (Rha4-1'), 100.8 (Rha2-1'), 104.0 (CH-3), 105.4 (C-10), 112.2 (CH-5'), 113.1

(CH-2'), 118.9 (CH-6'), 123.0 (C-1'), 146.8 (C-3'), 151.3 (C-4'), 157.1 (C-9), 161.6 (C-5), 162.5 (C-7), 164.1 (C-2), 182.0 (C-4).

Ds_3: Tiliroside = kaempferol-3-β-D-(6-O-trans-p-coumaroyl)glucopyranoside (Tsukamoto et al., 2004, 450) ^{1}H NMR (CD_3OD, 600 MHz) δ 3.45 (3H, m, H-2'', H-3'', H-5''), 4.19 (1H, dd, *J* = 11.8, 6.6 Hz, H-6''b), 4.29 (1H, dd, *J* = 11.8, 2.3 Hz, H-6''a), 5.25 (1H, d, *J* = 7.2 Hz, H-1''), 6.08 (1H, d, J = 15.9 Hz, H-8''), 6.14 (1H, d, *J* = 2.1 Hz, H-6), 6.33 (1H, d, *J* = 2.1 Hz, H-8), 6.80 (2H, d, *J* = 8.7 Hz, H-12'', H-14''), 6.83 (2H, d, *J* = 9.0 Hz, H-3', H-5'), 7.32 (2H, d, *J* = 8.7 Hz, H-11'', H-15''), 7.40 (1H, d, *J* = 15.9 Hz, H-9''), 8.00 (2H, d, *J* = 9.0 Hz, H-2', H-6'); 13C NMR (CD_3OD, 151 MHz) δ 64.2 (CH_2-6''), 75.8 (CH-3'', CH-5''), 78.1 (CH-2''), 94.8 (CH-8), 100.0 (CH-6), 103.9 (CH-1''), 105.5 (C-10), 114.8 (CH-8''), 116.0 (CH-3', CH-5'), 116.9 (CH-12'', CH-14''), 122.8 (C-1'), 127.1 (C-10''), 131.1 (CH-11'', CH-15''), 132.2 (CH-2', CH-6'), 146.6 (CH-9''), 158.5 (C-9), 159.4 (C-2), 161.2 (C-13''), 161.4 (C-4'), 162.8 (C-5), 165.9 (C-7), 168.8 (C-7'').
